# Supplementary material for: Elucidating the assembly of gas vesicles by systematic protein-protein interaction analysis
Source: EMBO J. 2024 Sep 3;43(19):4156–72. doi: 10.1038/s44318-024-00178-2 (PMC11445434; doi:10.1038/s44318-024-00178-2)
Supplement: Supplementary file 1 — Appendix [file 44318_2024_178_MOESM1_ESM.pdf]

Appendix

Elucidating the Assembly of Gas Vesicles by Systematic Protein-Protein Interaction Analysis

Authors: Manuel Iburg[1,3], Andrew P. Anderson[1,3], Vivian T. Wong[1], Erica D. Anton[1], Art He[1], George J. Lu[1,2,*]

Affiliations:

[1] Department of Bioengineering, Rice University, Houston, TX 77005, USA

[2] Department of BioSciences, Rice University, Houston, TX 77005, USA

[3] These authors contributed equally

* Correspondence: george.lu@rice.edu

Table of contents

3 Appendix Figure S1

4 Appendix Figure S2

5 Appendix Figure S3

7 Appendix Figure S4

9 Appendix Figure S5

10 Appendix Results

13 Appendix Discussion

17 Appendix References

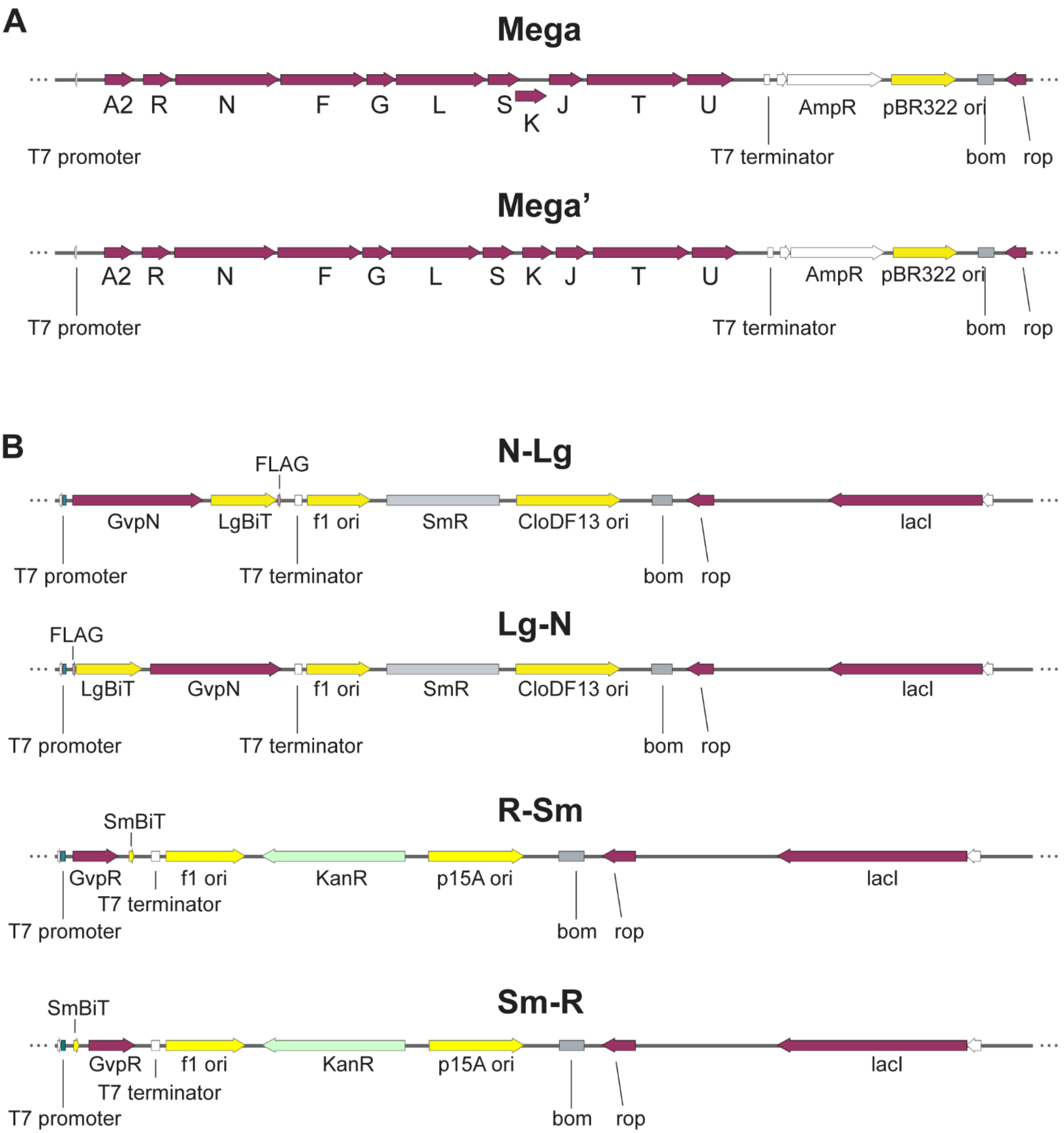

Appendix Figure S1. Overview of plasmid designs used in this study. Representative plasmid designs are shown in a linear form for ease of view. **(A)** The plasmid pST39-pNL29 ("Mega") encodes the proteins GvpA2, GvpR, GvpN, GvpF, GvpG, GvpL, GvpS, GvpK, GvpJ, GvpT, and GvpU (indicated by single letters) which can be expressed to generate recombinant GVs in *E. coli*. The plasmid contains a resistance marker for ampicillin (AmpR) and a pBR322 origin of replication. Mega' is identical to pSR39-pNL29, but the overlap between GvpS and GvpK has been resolved by molecular cloning. All

knockouts of one, two, or three GV proteins used in this study were derived from pST39-pNL29 or Mega'. **(B)** The plasmid N-Lg encodes GvpN-Linker-NanoLuc Large BiT-FLAG tag inducible by IPTG. The plasmid contains a resistance marker for Spectinomycin (SmR) and the CloDF13 origin of replication which is not in the same compatibility group as the pBR322 origin. Lg-N is identical to N-Lg but encodes FLAG-tag-NanoLuc Large BiT-Linker-GvpN and all other plasmids X-Lg or Lg-X are constructed analogously. The plasmid R-Sm encodes GvpR-Linker-NanoLuc Small BiT inducible by IPTG, and the FLAG-tag was not included. The plasmid contains a resistance gene for Kanamycin (KanR) and the p15A origin of replication which is not in the same compatibility group as the pBR322 and CloDF13 origins. Sm-R is identical to R-Sm but encodes NanoLuc Small BiT-Linker-GvpR and all other plasmids X-Sm or Sm-X are constructed analogously.

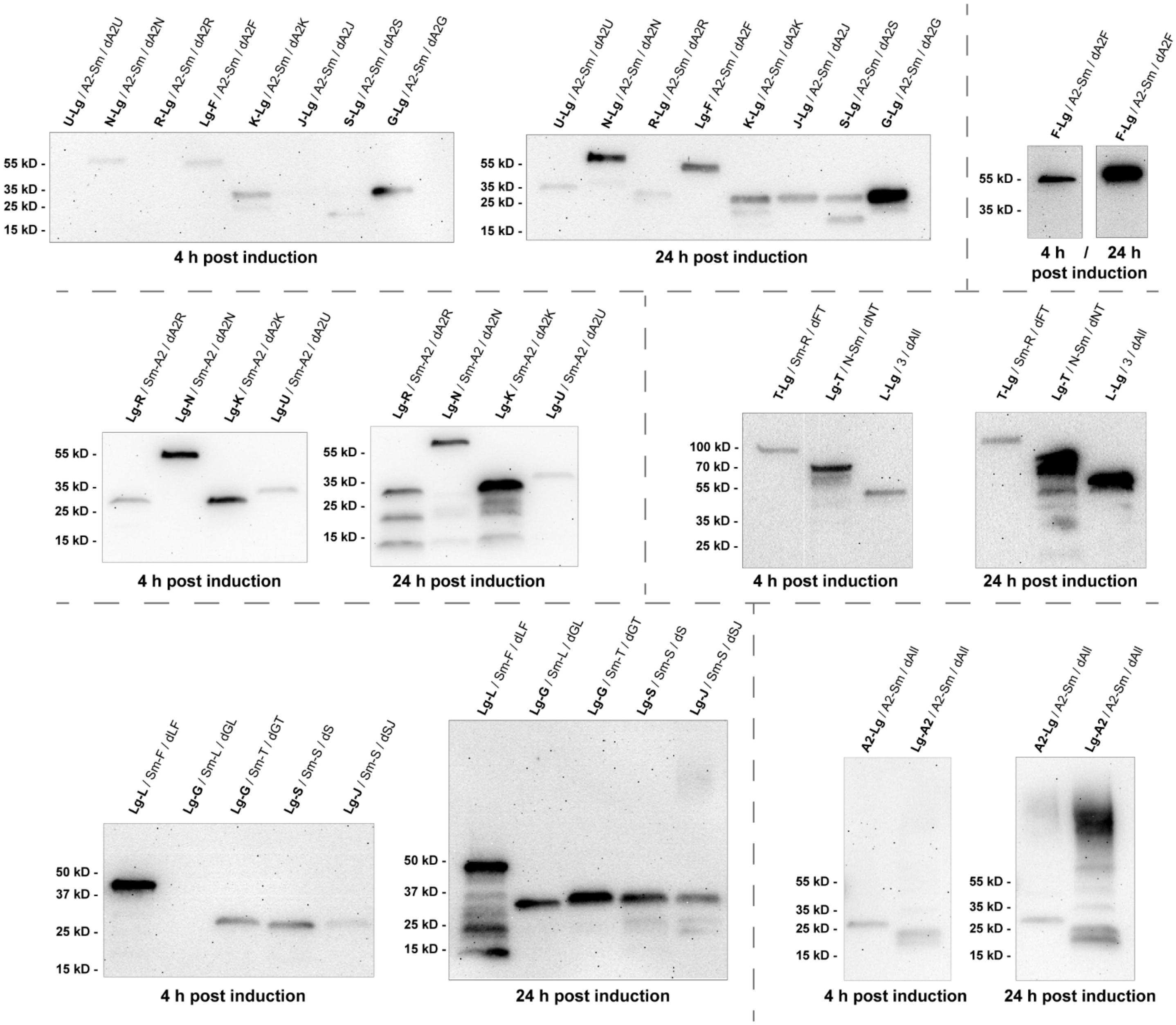

Appendix Figure S2. Western blot data to confirm expression of Gvp fusion proteins. FLAG tags were always included at the distal end of the Large BiT to allow for the detection of the expression of the fusion protein by western blotting (Figure S1B). We sampled a subset of the samples (indicated in Dataset EV1) to confirm the correct expression of our fusion proteins and to exclude the possibility that an absence of signal is due to an absence of protein expression. Each GvpX-Lg and Lg-GvpX was sampled in at least three independent instances and at least one instance of each is sampled for both t_4 and t_{24}, and the figure shows a representative example out of three for each fusion protein analyzed in this way.

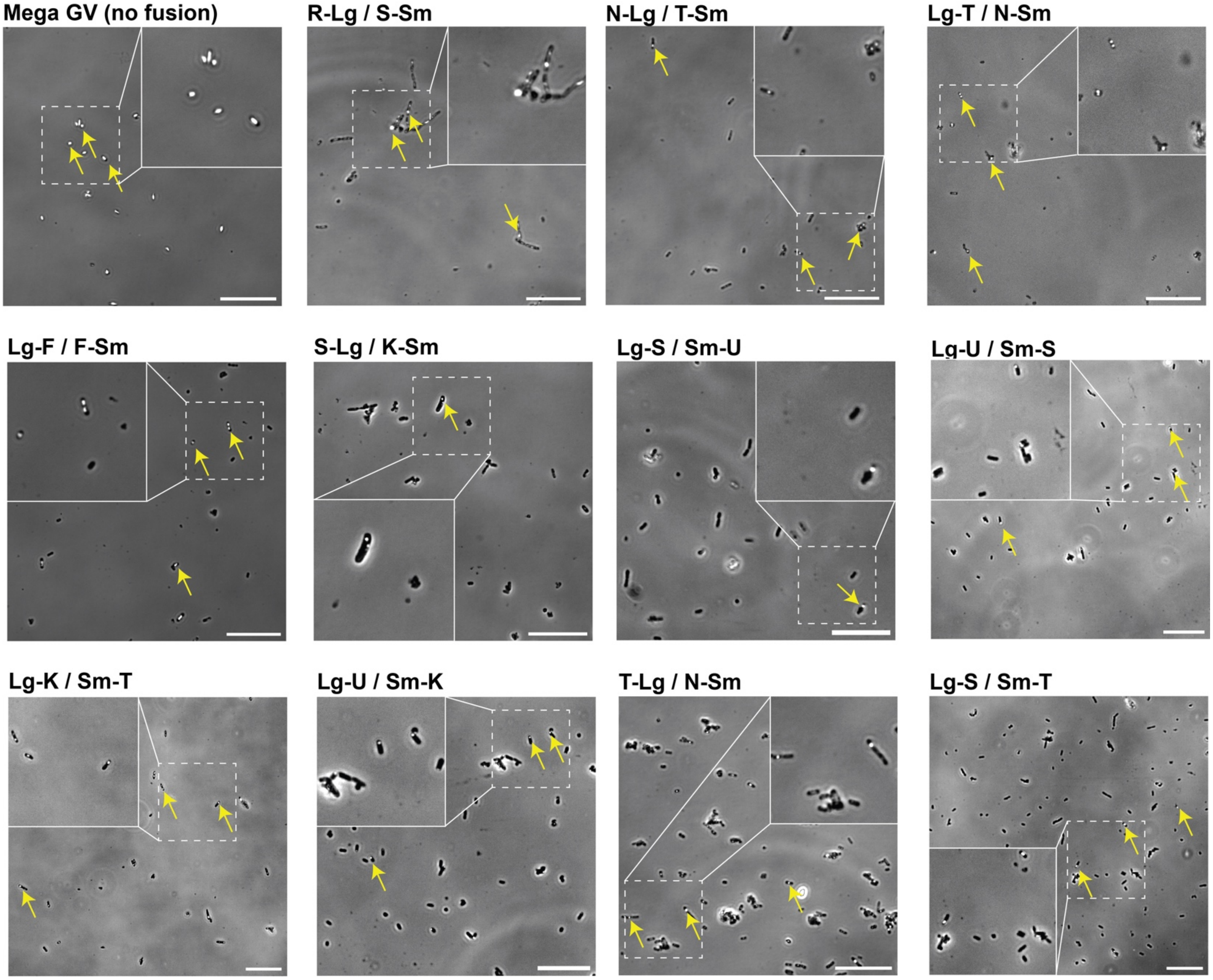

Appendix Figure S3. Phase-contrast images to examine the tolerance of the fusion partners to GV assembly factor proteins. For each fusion protein, at least three separate samples were taken for phase-contrast microscopy and analyzed for the existence of intact, successfully assembled GVs. We show the expression of GVs from the unmodified pNL29 operon and at least one for each GV fusion protein that gave rise to successfully assembled GVs (Figure 6C) as representative data. The scale bars represent 20 μm, and the white boxes mark a zoom-in view of the selected regions. Arrows indicate the representative GV-containing cells.

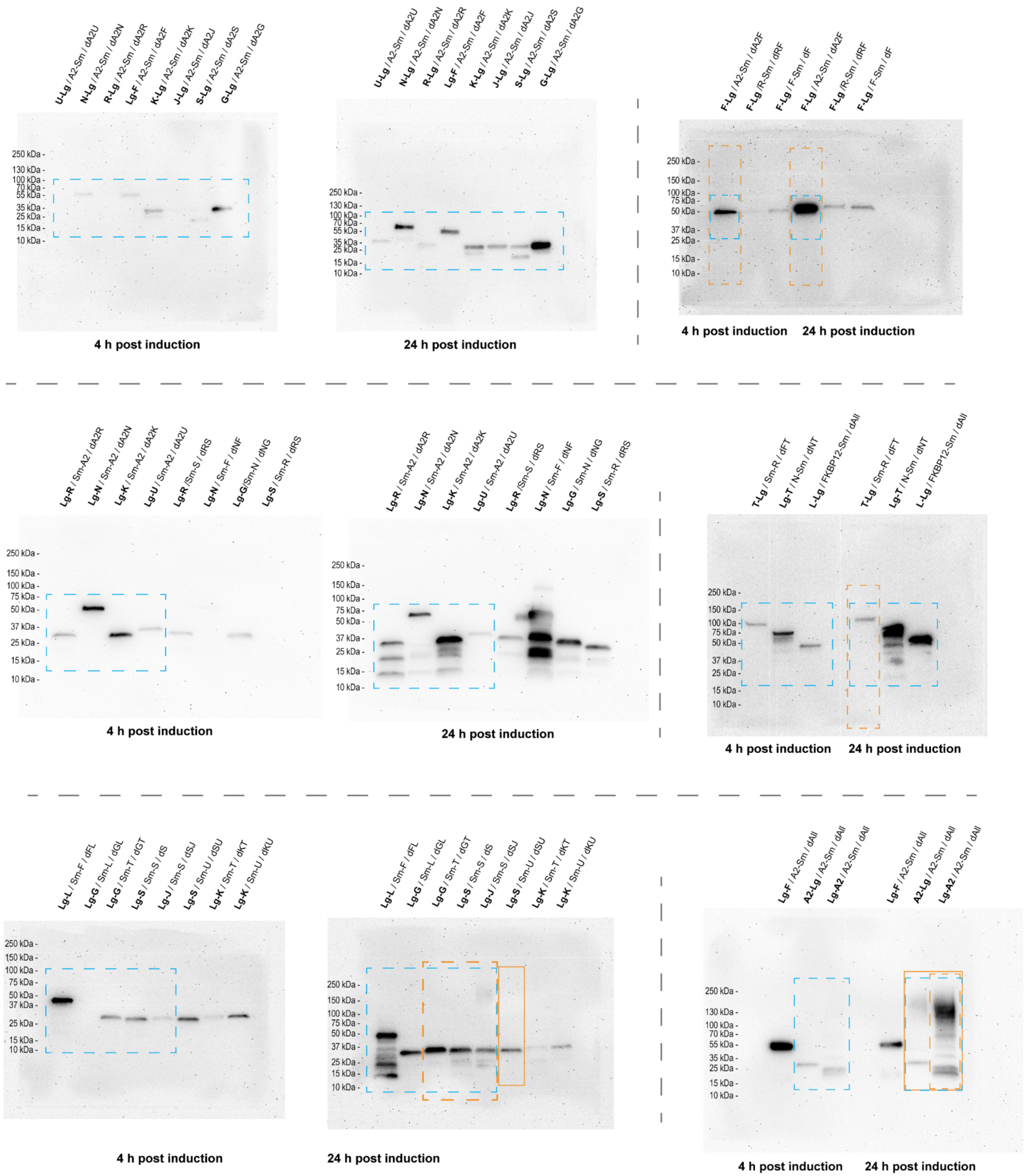

Appendix Figure S4. Uncropped western blot data to confirm expression of Gvp fusion proteins. FLAG tags were always included at the distal end of the Large BiT to allow for the detection of the

expression of the fusion protein by western blotting (**Appendix Figure S1B**). We sampled a subset of the samples to confirm the correct expression of our fusion proteins and to exclude the possibility that an absence of signal is due to an absence of protein expression. Each GvpX-Lg and Lg-GvpX was sampled in at least three independent instances and at least one instance of each is sampled for both t_4 and t_{24}, and the figure shows a representative example out of three for each fusion protein analyzed in this way. **Appendix Figure S4** depicts western blots arranged roughly in the same manner as in **Appendix Figure S2**, but uncropped. Orange boxes indicate the cropped area used for Figure 2F, dashed orange lines indicate the cropped area used for Figure 6DEF, and dashed blue lines indicate the cropped area used for **Appendix Figure S3**.

A

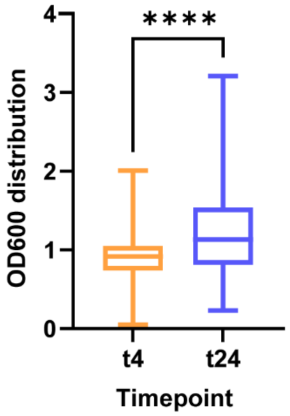

		Generic case	GV operon	Example	Visualization
B	Number of proteins	n	11	4	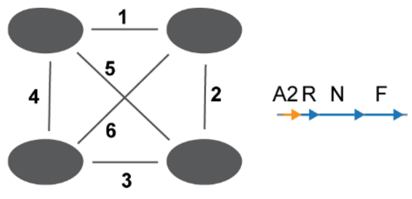
	Heterologous interaction	nC2=n!/(2*(n-2)!)	55	6	
C	Potential geometries	8	8	8	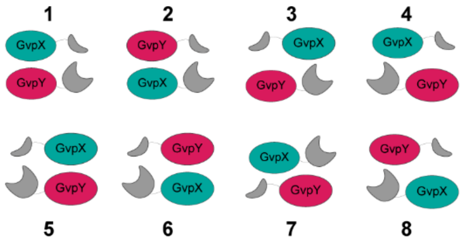
D	Potential backgrounds	n	11	4	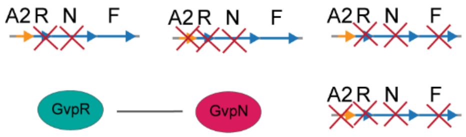
	Potential Experiments	4n!*n/(n-2)!	4840		
E	Homologous interaction		11	4	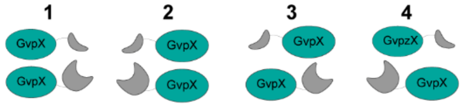
	Potential geometries	4	4	4	
F	Potential backgrounds	n+1	12	5	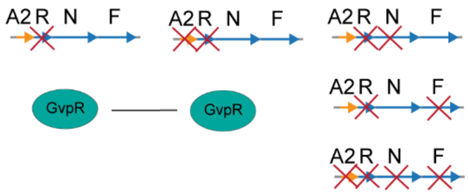
	Potential experiments	$4n^2+4n$	528		
	Total experiments	$4n^3+4n$	5368		

Appendix Figure S5. Overview of bacterial growth during measurements and in-depth consideration of all experimental conditions to assay

(**A**) A box and whiskers plot indicating the distribution of OD_{600} values of all samples measured at t_4 and t_{24} respectively, as described in the Methods section. Data is based on n = 1452 individual measurements per time point and the asterisks indicate a p-value of 0.0001. (**B-F**) A mathematical consideration of the theoretical number of protein-protein interaction experiments needed to screen all interactions in the GV operon with the split-luciferase complementation assay, as discussed in the results section and the Appendix Results section. A visual representation is provided for a smaller n number to illustrate. (**B**) Considering how many 1-on-1 interactions have to be assayed, we present an example for an n = 4 operon, resulting in 6 potential interactions to be assayed. (**C**) Due to the nature of the split-Nanoluciferase complementation assay, N- and C-terminal fusion proteins of the Large and Small BiTs may be tested on each of the two proteins under assay, so for each of the 1-on-1 assays, a total of 8 configurations exist. (**D**) For each interaction assayed, this can be done in the presence or absence of the other Gvps, or in a mixed case where some Gvps are included, and some are excluded. A visual example is given how the GvpR-GvpN interaction could be assayed in the presence of different backgrounds, resulting in a maximum of 4 conditions for each of the abovementioned. (**E**) For homologous interactions (GvpR-GvpR), a total of 4 could be screened in our example and each only results in 4 different geometries of fusion proteins as illustrated. (**F**) As before, each of the homologous interactions can be screened in the presence of all, some, or none of the other Gvps as visualized for an n = 4 operon. A generic case and a case for the operon discussed here (n = 11) are calculated in parallel.

Appendix to Results

Considerations for designing a modular plasmid framework to minimize cloning workload

Having decided to investigate all investigations in the *B. megaterium*-derived GV operon, we considered the number of conditions we would need to probe (**Figure 1D**). The pNL29 operon contains 11 genes (GvpA2, GvpR, GvpN, GvpF, GvpG, GvpL, GvpS, GvpK, GvpJ, GvpT, GvpU), and thus there is a total of 55 potential heterologous protein-protein interactions (consider: GvpA2 to GvpR; GvpA2 to GvpN; GvpA2 to GvpF;…GvpR to GvpN; GvpR to GvpF…etc.) and another 11 self-interactions (GvpA2 to GvpA2, GvpR to GvpR…etc.). The NanoLuc based split-luciferase complementation assay ("NanoBiT") requires a systematic toggling between the N- and C-terminal fusion and between the fusion of the larger or smaller fragments of the split luciferase to a given protein of interest, because we would not know *a priori* the orientation of the two binding partners, and placing the split luciferase

fragments on the wrong termini of the binding partners may sterically hinder the interaction and lead to false negatives. Also, adding a fusion partner may interfere with the native folding of the protein under assay, and thus it is desirable to screen both the large and small fragments for a given protein. For heterologous interaction pairs, this systematic toggling would multiply the number of conditions by 8 (consider: Lg-B to R-Sm, Lg-B to Sm-R, Lg-R to B-Sm etc…), and for self-interaction pairs, by 4, which brought the total number of conditions to 484. Thirdly, a critical parameter to assess for GV assembly is the potential dependence of an interaction pair on a third protein, which will aid in identifying protein complexes and constructing a roadmap of the assembly pathway. As the first step to probe the existence of such interdependence relations, we would assess the interaction of a pair of proteins under two conditions: without any background GV proteins and in the presence of all of them. If an interdependence condition exists, we would expect to see the former condition shows a negative result while the latter shows a positive. Adding this step would double the number of conditions, bringing the total number to 968. Lastly, if an interdependence condition is identified, it would be beneficial to probe the interdependence on individual GV proteins, which could add another 9 to 10 conditions to test for each specific interaction pair (consider: Lg-B to R-Sm without Gvps N, F, G; without GvpS N, F, L; without Gvps N, F, S…etc.). We grouped these as "additional" conditions that would be chosen for assay on a case-by-case basis (see also **Figure 1D** for a representation of the individual stages of testing and **Appendix Figure S5** for a mathematical overview of our considerations with a visual representation).

To cope with this large number of conditions, we decided to leverage the fact that multiple plasmids could be co-transformed into *E. coli*, which allows us to minimize the number of plasmids needed to construct and instead, rely on a mix-and-match of the plasmids to cover all the conditions. Thus, a three-plasmid system was designed and tested, of which each plasmid carried a different antibiotic resistance marker and origin of replication (**Figure 1E and Appendix Figure S1**). Plasmid I and II carried individual gas vesicle proteins fused with either Large BiT (Lg) or Small BiT (Sm), which are the two fragments of the split NanoLuc luciferase(Dixon *et al*, 2016b). Most importantly, Plasmid III contained either all the other GV proteins or an empty backbone, and this plasmid provided a handle to probe whether an interdependence relationship exists for the two proteins in Plasmid I and II. 67 Plasmids III were constructed initially to cover all the single, double, and full deletions for the 968 conditions expected above, and 14 additional triple-deletion Plasmids III were constructed to probe into the subnetworks of plausible functional complexes (see the sections titled "GvpA2-GvpF-GvpG form an interdependent interaction subnetwork" and "The GvpA2-GvpL interaction is dependent on GvpS, K and J"). Lastly, as a positive control of the split NanoLuc assay, two plasmids were created that carried

FKBP12-Sm and FRB-Lg proteins, which will dimerize strongly in the presence of rapamycin(Rivera *et al.*, 1996). Overall, this strategy successfully reduced the number of cloned plasmids to 128 (**Table EV1**). We then considered how to minimize the need to probe every conceivable geometry between any two Gvps. To this end, we prioritized assaying the constructs in which Large BiT was fused with the larger protein of the two interaction partners and Small BiT with the smaller one. We reasoned that the larger GV protein would be less likely to undergo changes in folding or solubility by a larger, globular fusion protein partner such as the LargeBiT. The major shell protein, GvpA2, was set as the exception, for which we assayed all configurations, because GvpA2 is central in the assembly process. For simplicity, the initial testing set included the split NanoLuc all fused to the C-terminus of GV proteins before carrying out additional screening of the fusion at N/C, C/N, or N/N terminus of GV proteins. We reasoned that if a positive interaction is observed under any of the initial C/C fusion conditions, we would no longer need to test the additional fusion constructs.

Considerations for focusing exclusively on *in vivo* protein-protein interaction analysis

In a previous study (Volkner *et al*, 2020), an *in vivo* protein-protein interaction screen of gas vesicle proteins from *H. salinarum* was supplemented by *in vitro* column-based pull-down interaction measurements of select interaction partners. This experiment is the most direct measurement of protein interaction and reduces the possibility that any measured interaction is dependent on an unknown third protein (that could be derived from the heterologous expression host). In our screening, we focused solely on *in vivo* protein-protein interactions following these considerations:

Since we assay about 1000 protein interactions in approximately 3000 experiments, we would have to add a substantial amount of *in vitro* pull-down assays or reduce the breadth of our screen to match all *in vivo* experiments with *in vitro* confirmation. Additionally, we consider the *in vivo* experiment more relevant to the actual process of GV assembly (*in vitro* assembly of GVs has not been reported so far). Finally, while *in vitro* protein-protein interaction studies are a strong complement to *in vivo* studies, we anticipate that co-purification of host proteins or denaturing of proteins during cell lysis could likewise introduce artifact measurements to our study. To conclude, we prioritized completeness of screening over depth for this study.

Appendix to Discussion

Notable consistencies / diverging observations with studies on the *H. salinarum* GV operon protein-protein interaction network

It is important to put our findings in the context of recent studies of the GV proteins in the halophile archaeon *H. salinarum* that were carried out *in vitro* by means of pull-down and *in* vivo with a split-GFP reporter using *H. volcanii* as a host organism. Firstly, we note that in these studies (Jost & Pfeifer, 2022;

Tavlaridou *et al*, 2014; Volkner *et al.*, 2020; Winter *et al*, 2018) interactions were screened in the absence of the remaining *H. salinarum* GV proteins in a manner matching the data found in Figure 4 C&D of this study. The primary interaction between GvpA2 and GvpF (Figure 4D) matches the interaction of GvpA and GvpF (Volkner *et al.*, 2020). Conversely, the same study did not observe strong interactions between GvpA and GvpL or GvpG (Volkner *et al.*, 2020), which we saw for GvpB (Figure 4D). This is not in contradiction to our findings, as we found that GvpB and GvpG would only interact in the presence of GvpF (Figure 5B) whereas the GvpB to GvpL interaction depends on GvpS, K and J (Figure 5D), conditions that were not part of the screening by Völkner *et al.*

Further corroborating our findings, *H. salinarum* GvpM, K and J are homologs of GvpS, K and J (Pfeifer, 2012) and both groups of GV proteins were observed to be interaction partners of GvpL. Moreover, GvpL was indeed hypothesized to be a scaffold of GV assembly which supports our interpretation ((Volkner *et al.*, 2020); Figure 3C). The strongest interaction reported by Völkner *et al* is GvpL to GvpG – which is notably absent in our analysis, although we do find a strong interaction between GvpL and GvpF (Figure 3C, Figure 4D). Likewise, we do not observe the interaction between GvpM and GvpJ ((Tavlaridou *et al.*, 2014); GvpS and GvpJ in this study) (Figure 4D). Lastly, *H. salinarum* GvpO is a homolog of GvpR (Pfeifer, 2012) and interacts with *H. salinarum* GvpA (homolog of GvpA2), as well as itself, GvpL and GvpN (Jost & Pfeifer, 2022), which matches our observations, albeit only in the presence of the remaining GV proteins (Figure 3C). In conclusion we confirmed several conserved mechanisms in GV assembly, e.g. the need for dedicated binders of shell proteins (such as GvpF) and the initiation of GV assembly mediated by GvpK, L and homologs of the major shell protein. Disparities in the observed interactions hint that some of the functions of homologous proteins may be distributed differently across domains (e.g. between GvpF and GvpL). In some cases, proteins are unique to the GV operon of *B. megaterium* (GvpU, GvpT) or *H. salinarum* (GvpH, GvpI) and these proteins might mediate the cell-specific organization of GVs or fulfill analogous roles without being protein homologs.

Granular observations on the interactions of all Gvps in the context of published data

To make the dense nature of data presented in Figures 3, 4 and Dataset EV1 more accessible, we summarize both our findings and previously published results on the role of each Gvp in the following: **GvpA2** is the major constituent of the GV shell (Dutka *et al*, 2023; Huber *et al*, 2023; Sivertsen *et al*, 2010) and its hydrophobic properties lead to protein aggregation and cytotoxicity upon overexpression ((Jung *et al*, 2021; Pfeifer, 2022); Figures 4E & 6D), while fusion proteins of **GvpA2** prevent the assembly of GVs (Figure 6C). The primary interaction partner and hypothetical chaperone of **GvpA2** is **GvpF** (Figure 4C; (Volkner *et al.*, 2020)), which transfers it to **GvpG** as a secondary interaction partner (Figure 5B). After binding **GvpG**, **GvpA2** might access growing GVs *via* an interaction with **GvpL** which

increases over the course of GV assembly (Figure 3 C&F, Table S2). This interaction is dependent on the presence of **GvpS, K and J**, indicating that these proteins precede the introduction of **GvpB** to the GV, possibly as part of a seeding complex (Figure 5D, (Volkner *et al.*, 2020)).

GvpR interacts with itself and **GvpN, G, L, J** (Figure 3C), interactions which are partly diminished in the absence of the GV operon (Figure 4C & D). The interaction with **GvpL** is dependent on **GvpS and J** as much as the interaction with **GvpJ** is dependent on **GvpL**, indicating an involvement in the **SKJL** interaction subnetwork. Notably, **GvpR** is non-essential for GV formation (Farhadi *et al*, 2019), but all fusion proteins except R-Lg prevent GV formation. We conclude that while **GvpR** has an auxiliary function, its central position in GV assembly can lead to a counterproductive gain of function.

GvpN is an AAA+ ATPase (Jost & Pfeifer, 2022) and interacts with itself (Figure 3C), which is in agreement with AAA+ protein specific homooligomers (Mogk *et al*, 2008). **GvpN** interacts with **GvpR, J** and most prominently, with **GvpS**. In the absence of the GV operon, an interaction with **GvpA2** emerges, and the interaction with **GvpS** is lost (Figure 4C & D). C-terminal fusions to **GvpN** do not prevent the formation of GVs (Figure 6C). The deletion of **GvpN** leads to a short-GV phenotype (Pfeifer, 2012), pointing to a role in elongating GVs by enabling the insertion of **GvpA2**.

GvpF is essential to GV formation (Offner *et al*, 2000) and only the F-Sm fusion protein does not prohibit the formation of GVs. Besides the notable interaction with **GvpA2 and G** (Figure 3C; Figure 4C & D; Figure 5A), **GvpF** interacts with **GvpN and S**, the latter being a homolog of **GvpA2** (Figure 4E; (Pfeifer, 2022)).

GvpG has an exceptionally strong interaction with **GvpA2** (Figure 3C), which is dependent on and supersedes the interaction of **GvpA2** and **GvpF** (Figure 4 C & D; Figure 5A & B). It is possible that **GvpG** stabilizes **GvpA2** before its insertion into the growing GV, as **GvpG** also interacts with **GvpS, K, J, R and F** (Figure 3C; Figure 5A). In line with its central role in binding **GvpA2**, fusion proteins of **GvpG** prevent the formation of GVs (Figure 6C) and it is essential for GV formation (Offner *et al.*, 2000).

GvpL interacts with **GvpS, K, J and R**, an interaction required for binding **GvpA2** (Figure 3C; Figure 5D; Table S2) and may act as a scaffold that coordinates the interaction of Gvps (Volkner *et al.*, 2020), making it essential to GV assembly (Offner *et al.*, 2000).

GvpS interacts with **GvpA2, L, K and J** (Figure 3C; Figure 5C) and also interacts with **GvpF, G** and most strongly **GvpN**. In the absence of the GV operon, the strong interaction with the GV elongation factor **GvpN** disappears (Figure 4 C & D; (Pfeifer, 2012)), indicating that **GvpS** is involved with the activity of **GvpN**.

GvpK is essential for the formation of GVs (Offner *et al.*, 2000), but itself not part of the structure (Chu *et al*, 2011; Shukla & DasSarma, 2004) and is also part of the **A2LSKJ** interaction subnetwork (Figure

3C; Figure 5D). **GvpK** has a strong interaction with **GvpG** but is still tolerant of fusion proteins (Figure 6C). Deletion of the GV operon removes the interaction with **GvpS** but strengthens the interaction with **GvpB, J** and itself (Figure 4C & D).

GvpJ interacts with all other Gvps (most notably itself and **GvpL**, possible exception of **GvpF**) (Figure 3C), is a homolog of **GvpA2** (Figure 4E; (Pfeifer, 2022)), that emulates the aggregation prone behavior (Figure 6D; (Pfeifer, 2022)) and no fusion proteins of **GvpJ** allow for GV formation (Figure 6C). Since **GvpJ** is a part of the **BLSKJ** interaction subnetwork (Figure 5D) and most single AA mutations in GvpJ lead to GV negative cells, **GvpJ** may be a part of the initial GV seeding (Knitsch, 2021).

GvpT interacts with itself, and Western blotting indicates that this is in the form of SDS-resistant dimers (Figure 3C, Figure 6F). It has a strong interaction with **GvpG,** which drops over time and its strong interaction with **GvpJ** and itself increases over time (Figure 3C & F) and disappears in the absence of the GV operon (Figure 4C & D). **GvpT** is non-essential to GV formation (Farhadi *et al.*, 2019) and consequently **GvpT** fusion proteins do not prevent the appearance of GVs (Figure 6C). Deletions of **GvpT** lead to a reduction in hydrodynamic radius purified GVs, indicating a potential role in their clustering (Li *et al*, 2023).

GvpU is non-essential to the initial assembly of GVs but promotes their clustering mediated by liquid-liquid phase separation (Li *et al.*, 2023). **GvpU** interacts with **GvpJ, L** and itself (Figure 3C), interactions that diminish over time (Figure 3F) or in the absence of the GV operon (Figure 4C & D). In turn, deletion of the operon leads to a strong interaction between **GvpU** and **GvpR and G** (Figure 4C & D). Despite being non-essential, C-terminal fusions of **GvpU** prevent the formation of GVs (Figure 6C), possibly because **GvpU** interacts with **GvpA2** even before GV assembly *via* the interaction with **GvpR, G, L and J** (Figure 3C; Figure 4C &D; (Li *et al.*, 2023)).

REFERENCE:

Bourdeau RW, Lee-Gosselin A, Lakshmanan A, Farhadi A, Kumar SR, Nety SP, Shapiro MG (2018) Acoustic reporter genes for non-invasive imaging of microorganisms in mammalian hosts. *Nature* 553: 86-90

Chu LJ, Chen MC, Setter J, Tsai YS, Yang H, Fang X, Ting YS, Shaffer SA, Taylor GK, von Haller PD *et al* (2011) New structural proteins of Halobacterium salinarum gas vesicle revealed by comparative proteomics analysis. *J Proteome Res* 10: 1170-1178

Dixon AS, Schwinn MK, Hall MP, Zimmerman K, Otto P, Lubben TH, Butler BL, Binkowski BF, Machleidt T, Kirkland TA *et al* (2016a) NanoLuc Complementation Reporter Optimized for Accurate Measurement of Protein Interactions in Cells. *ACS Chem Biol* 11: 400-408

Dixon AS, Schwinn MK, Hall MP, Zimmerman K, Otto P, Lubben TH, Butler BL, Binkowski BF, Machleidt T, Kirkland TA *et al* (2016b) NanoLuc Complementation Reporter Optimized for Accurate Measurement of Protein Interactions in Cells. *ACS Chem Biol* 11: 400-408

Dutka P, Metskas LA, Hurt RC, Salahshoor H, Wang TY, Malounda D, Lu GJ, Chou TF, Shapiro MG, Jensen GJ (2023) Structure of Anabaena flos-aquae gas vesicles revealed by cryo-ET. *Structure* 31: 518-528 e516

Farhadi A, Ho GH, Sawyer DP, Bourdeau RW, Shapiro MG (2019) Ultrasound imaging of gene expression in mammalian cells. *Science* 365: 1469-1475

Hopp TP, Prickett KS, Price VL, Libby RT, March CJ, Pat Cerretti D, Urdal DL, Conlon PJ (1988) A Short Polypeptide Marker Sequence Useful for Recombinant Protein Identification and Purification. *Biotechnology (N Y)* 6: 1204-1210

Huber ST, Terwiel D, Evers WH, Maresca D, Jakobi AJ (2023) Cryo-EM structure of gas vesicles for buoyancy-controlled motility. *Cell* 186: 975-986 e913

Jost A, Pfeifer F (2022) Interaction of the gas vesicle proteins GvpA, GvpC, GvpN, and GvpO of Halobacterium salinarum. *Front Microbiol* 13: 971917

Jung H, Ling H, Tan YQ, Chua NH, Yew WS, Chang MW (2021) Heterologous expression of cyanobacterial gas vesicle proteins in Saccharomyces cerevisiae. *Biotechnol J* 16: e2100059

Knitsch R, 2021. Mutationen in GvpA und GvpJ –
Eine Mutationsanalyse zweier essentieller
Gasvesikelproteine, FB Biologie. TU Darmstadt, Darmstadt.

Kuhlman B, Yang HY, Boice JA, Fairman R, Raleigh DP (1997) An exceptionally stable helix from the ribosomal protein L9: implications for protein folding and stability. *J Mol Biol* 270: 640-647

Li N, Cannon MC (1998) Gas Vesicle Genes Identified in Bacillus megaterium and Functional Expression in Escherichia coli. *J Bacteriol* 180: 2450-2458

Li Z, Shen Q, Dai Y, Anderson AP, Iburg M, Lin R, Zimmer B, Meyer MD, You L, Chilkoti A *et al* (2023) Spatial Organization of Gas Vesicles is Governed
by Phase-separable GvpU.

Mogk A, Haslberger T, Tessarz P, Bukau B (2008) Common and specific mechanisms of AAA+ proteins involved in protein quality control. *Biochem Soc Trans* 36: 120-125

Offner S, Hofacker A, Wanner G, Pfeifer F (2000) Eight of fourteen gvp genes are sufficient for formation of gas vesicles in halophilic archaea. *J Bacteriol* 182: 4328-4336

Pfeifer F (2012) Distribution, formation and regulation of gas vesicles. *Nat Rev Microbiol* 10: 705-715

Pfeifer F (2022) Recent Advances in the Study of Gas Vesicle Proteins and Application of Gas Vesicles in Biomedical Research. *Life (Basel)* 12

Rivera VM, Clackson T, Natesan S, Pollock R, Amara JF, Keenan T, Magari SR, Phillips T, Courage NL, Cerasoli Jr F *et al* (1996) A humanized system for pharmacologic control of gene expression. *Nat Med* 2: 1028

Shukla HD, DasSarma S (2004) Complexity of gas vesicle biogenesis in Halobacterium sp. strain NRC-1: identification of five new proteins. *J Bacteriol* 186: 3182-3186

Sivertsen AC, Bayro MJ, Belenky M, Griffin RG, Herzfeld J (2010) Solid-state NMR characterization of gas vesicle structure. *Biophys J* 99: 1932-1939

Tavlaridou S, Winter K, Pfeifer F (2014) The accessory gas vesicle protein GvpM of haloarchaea and its interaction partners during gas vesicle formation. *Extremophiles* 18: 693-706

Volkner K, Jost A, Pfeifer F (2020) Accessory Gvp Proteins Form a Complex During Gas Vesicle Formation of Haloarchaea. *Front Microbiol* 11: 610179

Winter K, Born J, Pfeifer F (2018) Interaction of Haloarchaeal Gas Vesicle Proteins Determined by Split-GFP. *Front Microbiol* 9: 1897
