# Supplementary figures and images for: Elucidating the assembly of gas vesicles by systematic protein-protein interaction analysis
Source: EMBO J. 2024 Sep 3;43(19):4156–72. doi: 10.1038/s44318-024-00178-2 (PMC11445434; doi:10.1038/s44318-024-00178-2)

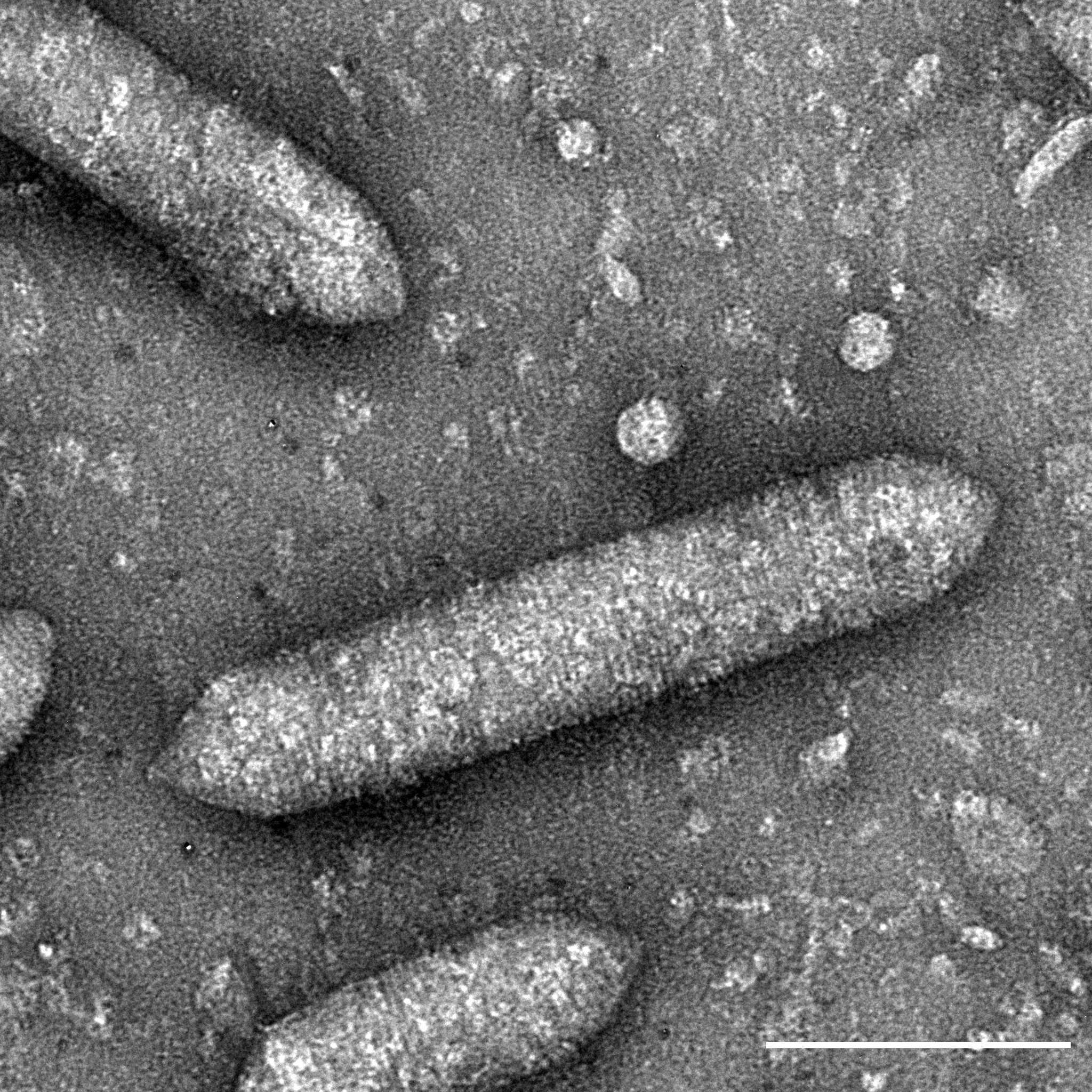

Supplement: Supplementary file 8 — Source data Fig. 1 [file 44318_2024_178_MOESM8_ESM.zip › Figure 1/1A/MI_MegaGV_2_40000x.tif]

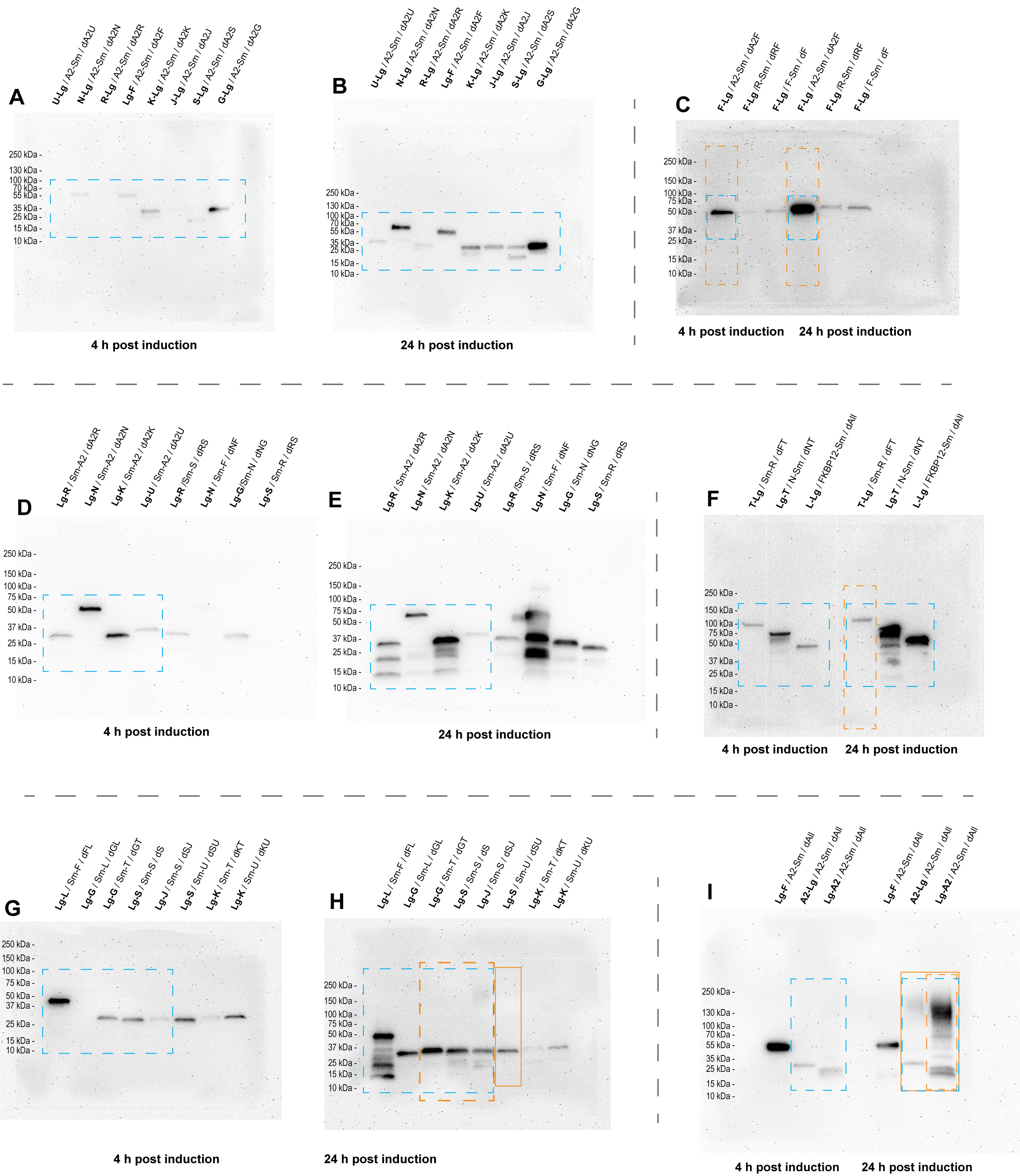

Supplement: Supplementary file 9 — Source data Fig. 2 [file 44318_2024_178_MOESM9_ESM.zip › Figure 2/2F/EMBOJ-2023-115793_AppendixFigureS4_VerA2.tif]

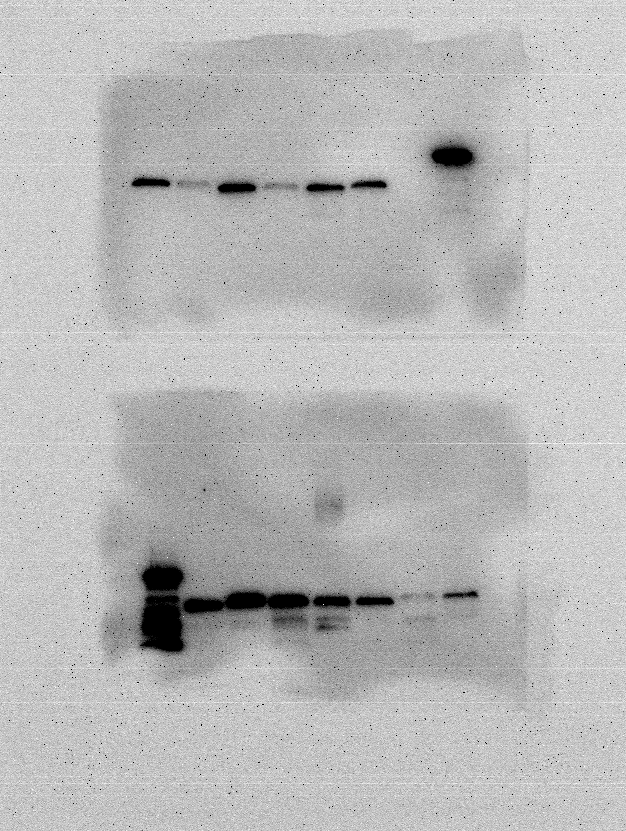

Supplement: Supplementary file 9 — Source data Fig. 2 [file 44318_2024_178_MOESM9_ESM.zip › Figure 2/2F/G, H/Anti-FLAG/2021-12-29_13-09-44_1_16bit strong.tif]

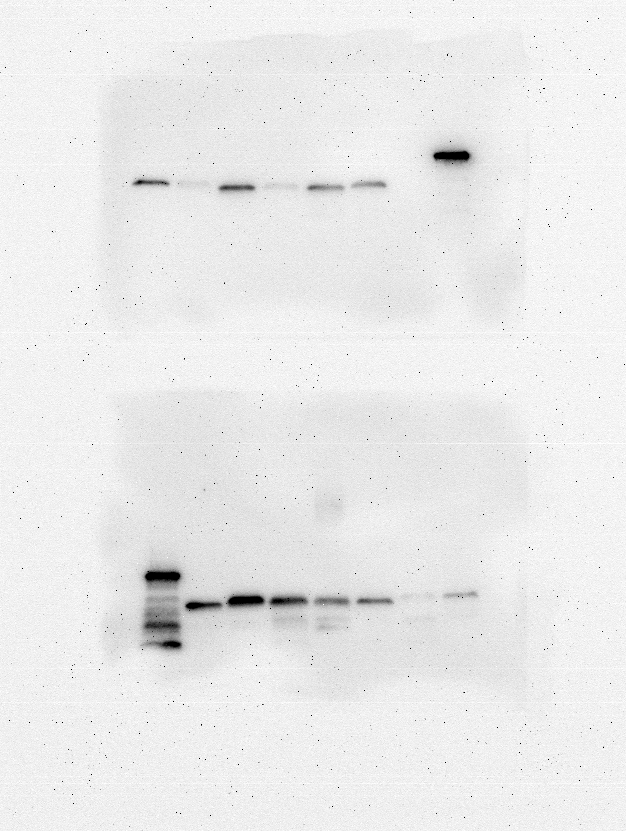

Supplement: Supplementary file 9 — Source data Fig. 2 [file 44318_2024_178_MOESM9_ESM.zip › Figure 2/2F/G, H/Anti-FLAG/2021-12-29_13-09-44_1_16bit-1.tif]

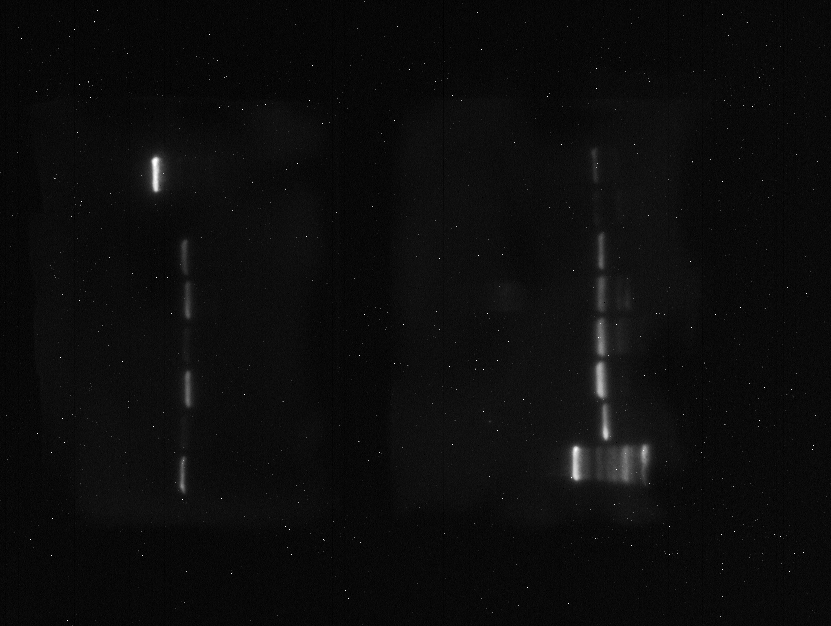

Supplement: Supplementary file 9 — Source data Fig. 2 [file 44318_2024_178_MOESM9_ESM.zip › Figure 2/2F/G, H/Anti-FLAG/2021-12-29_13-09-44_1_16bit.png]

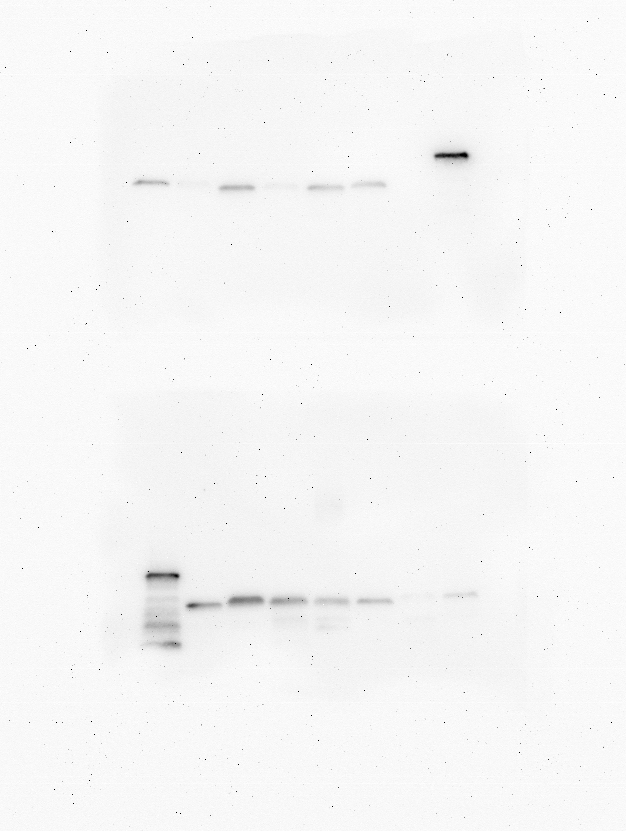

Supplement: Supplementary file 9 — Source data Fig. 2 [file 44318_2024_178_MOESM9_ESM.zip › Figure 2/2F/G, H/Anti-FLAG/2021-12-29_13-09-44_1_16bit.tif]

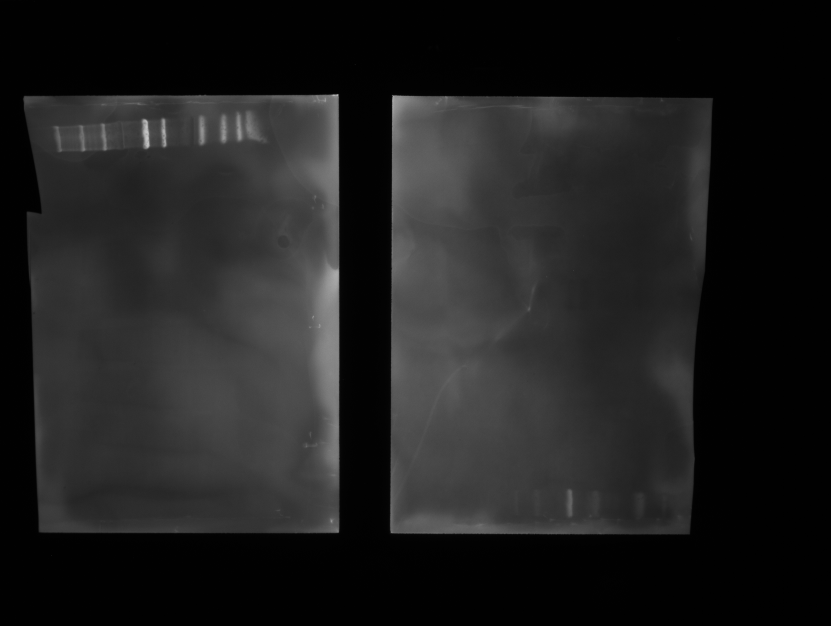

Supplement: Supplementary file 9 — Source data Fig. 2 [file 44318_2024_178_MOESM9_ESM.zip › Figure 2/2F/G, H/Anti-FLAG/2021-12-29_13-09-44_2_16bit.png]

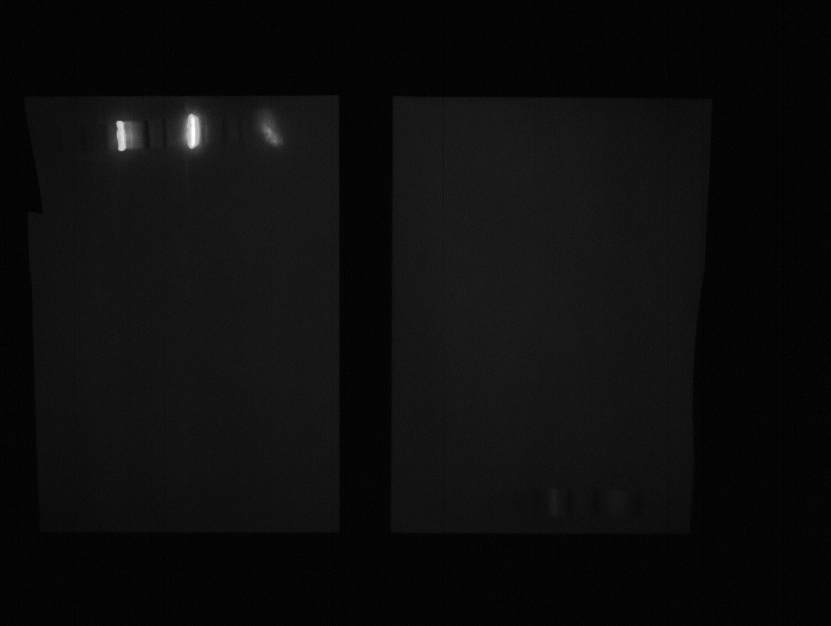

Supplement: Supplementary file 9 — Source data Fig. 2 [file 44318_2024_178_MOESM9_ESM.zip › Figure 2/2F/G, H/Anti-FLAG/2021-12-29_13-09-44_3_16bit.png]

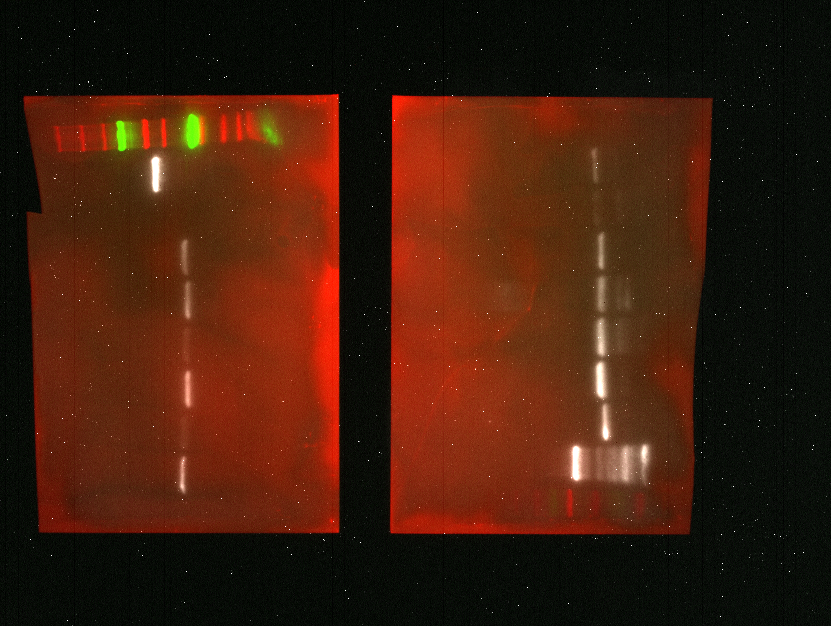

Supplement: Supplementary file 9 — Source data Fig. 2 [file 44318_2024_178_MOESM9_ESM.zip › Figure 2/2F/G, H/Anti-FLAG/2021-12-29_13-09-44_8bit.png]

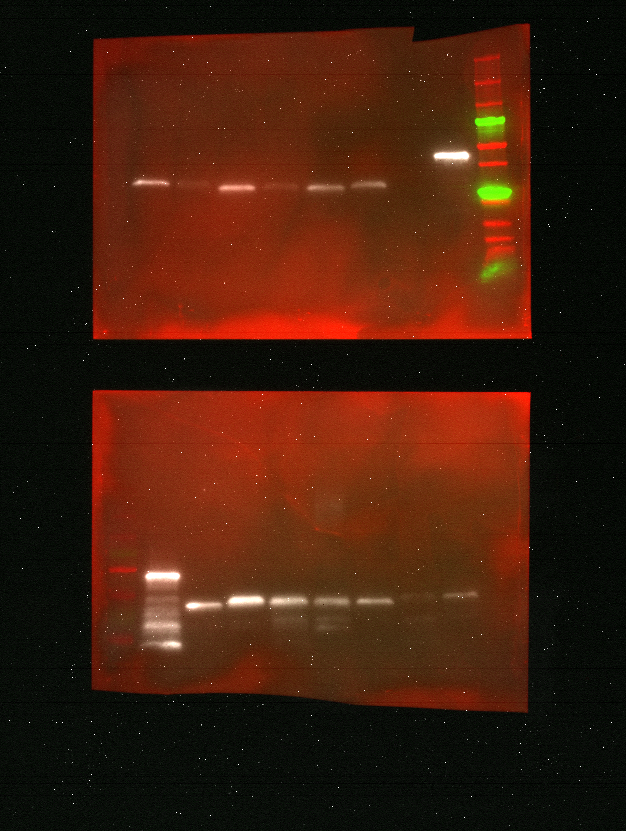

Supplement: Supplementary file 9 — Source data Fig. 2 [file 44318_2024_178_MOESM9_ESM.zip › Figure 2/2F/G, H/Anti-FLAG/2021-12-29_13-09-44_8bit.tif]

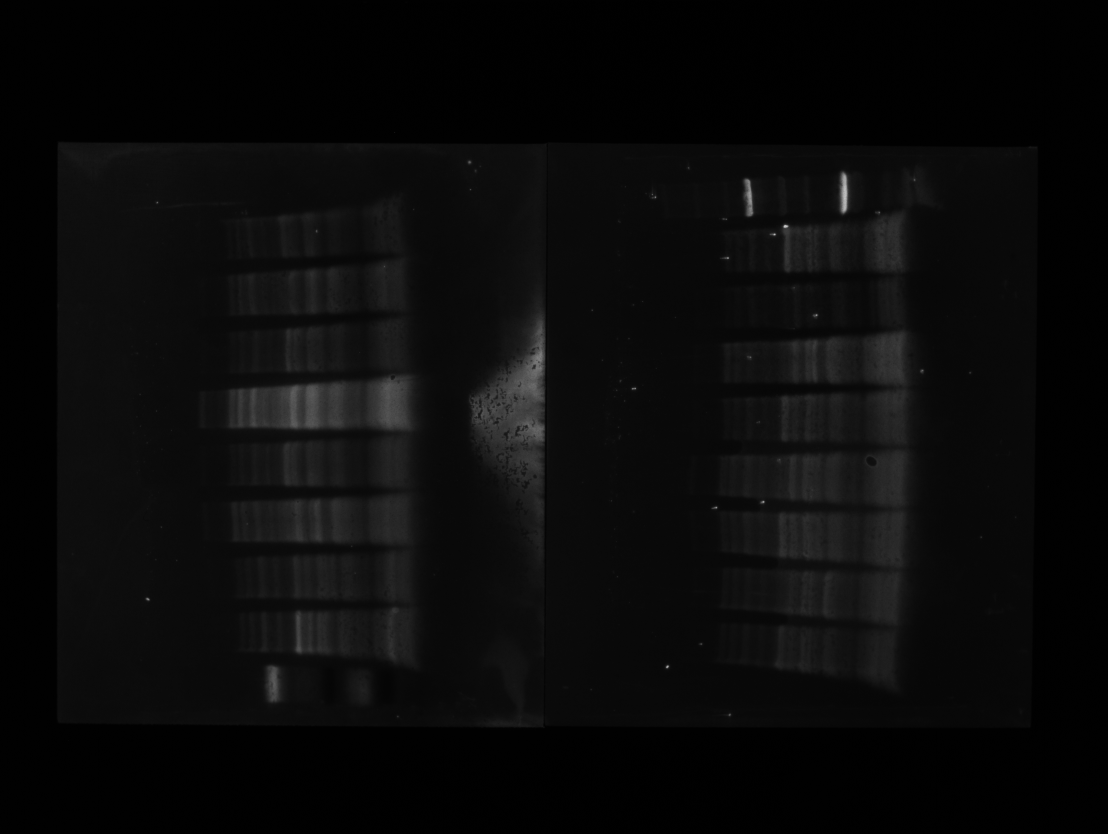

Supplement: Supplementary file 9 — Source data Fig. 2 [file 44318_2024_178_MOESM9_ESM.zip › Figure 2/2F/G, H/Total protein/2021-12-28_15-07-55_1_16bit.png]

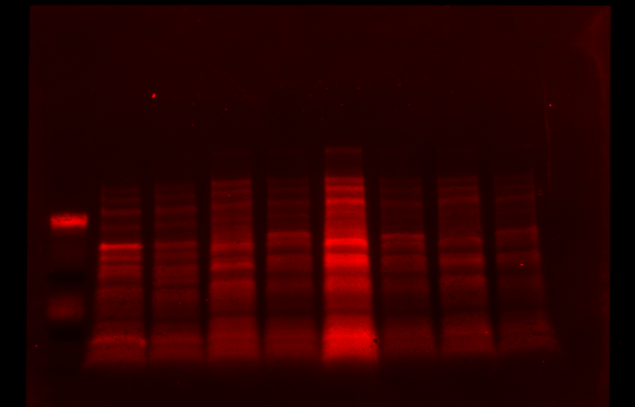

Supplement: Supplementary file 9 — Source data Fig. 2 [file 44318_2024_178_MOESM9_ESM.zip › Figure 2/2F/G, H/Total protein/2021-12-28_15-07-55_8bit-1 t24.png]

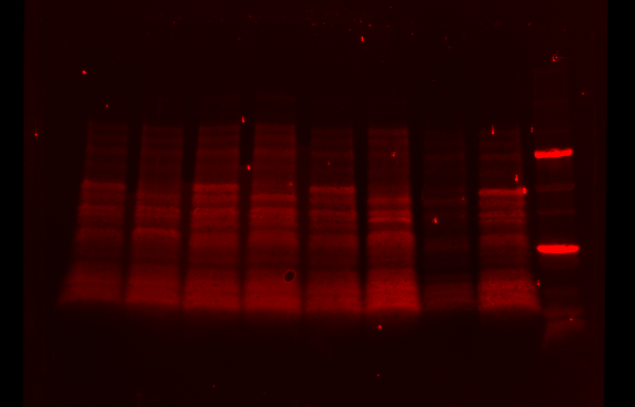

Supplement: Supplementary file 9 — Source data Fig. 2 [file 44318_2024_178_MOESM9_ESM.zip › Figure 2/2F/G, H/Total protein/2021-12-28_15-07-55_8bit-1 t4.png]

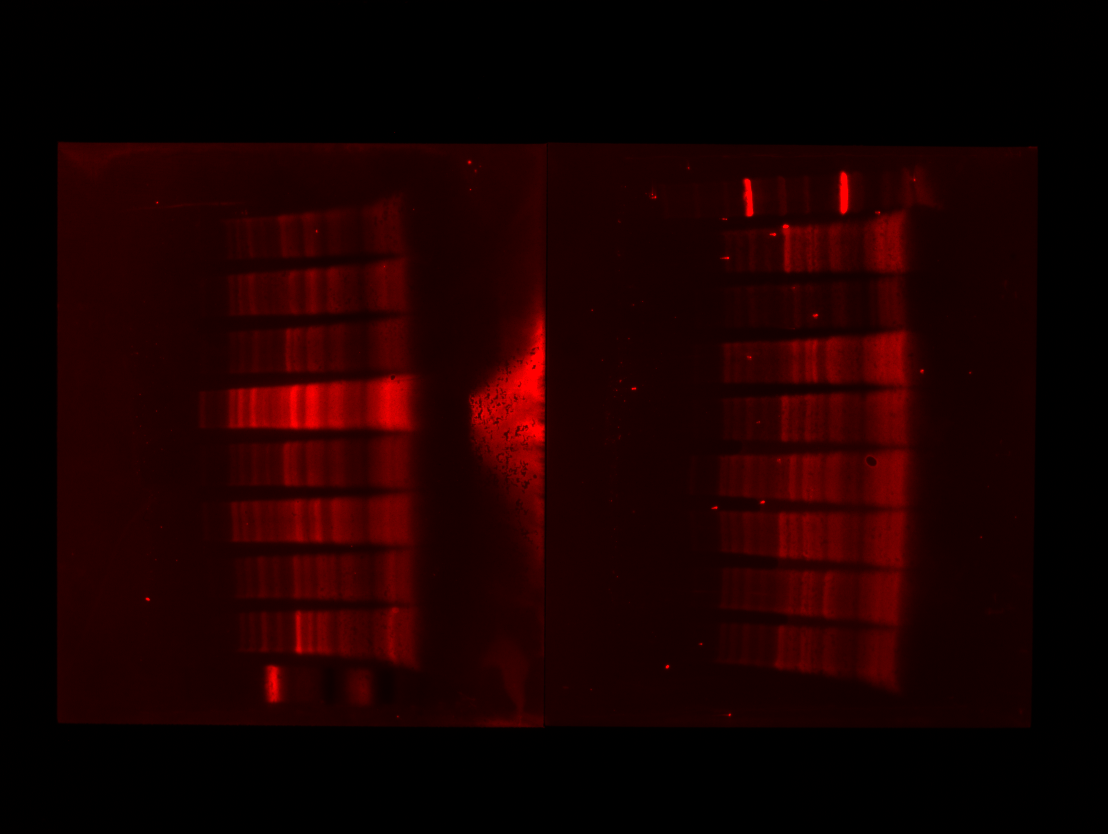

Supplement: Supplementary file 9 — Source data Fig. 2 [file 44318_2024_178_MOESM9_ESM.zip › Figure 2/2F/G, H/Total protein/2021-12-28_15-07-55_8bit.png]

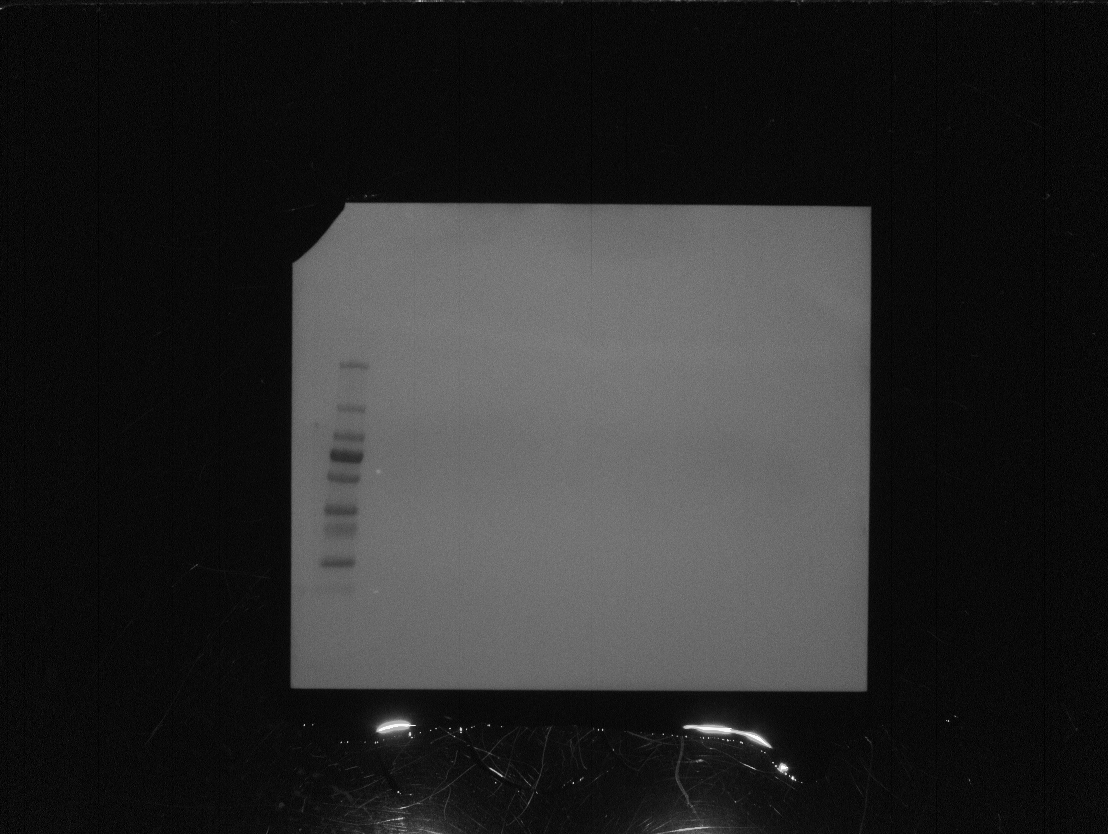

Supplement: Supplementary file 9 — Source data Fig. 2 [file 44318_2024_178_MOESM9_ESM.zip › Figure 2/2F/I/Anti-FLAG/2021-10-09_15-27-44_1_16bit.png]

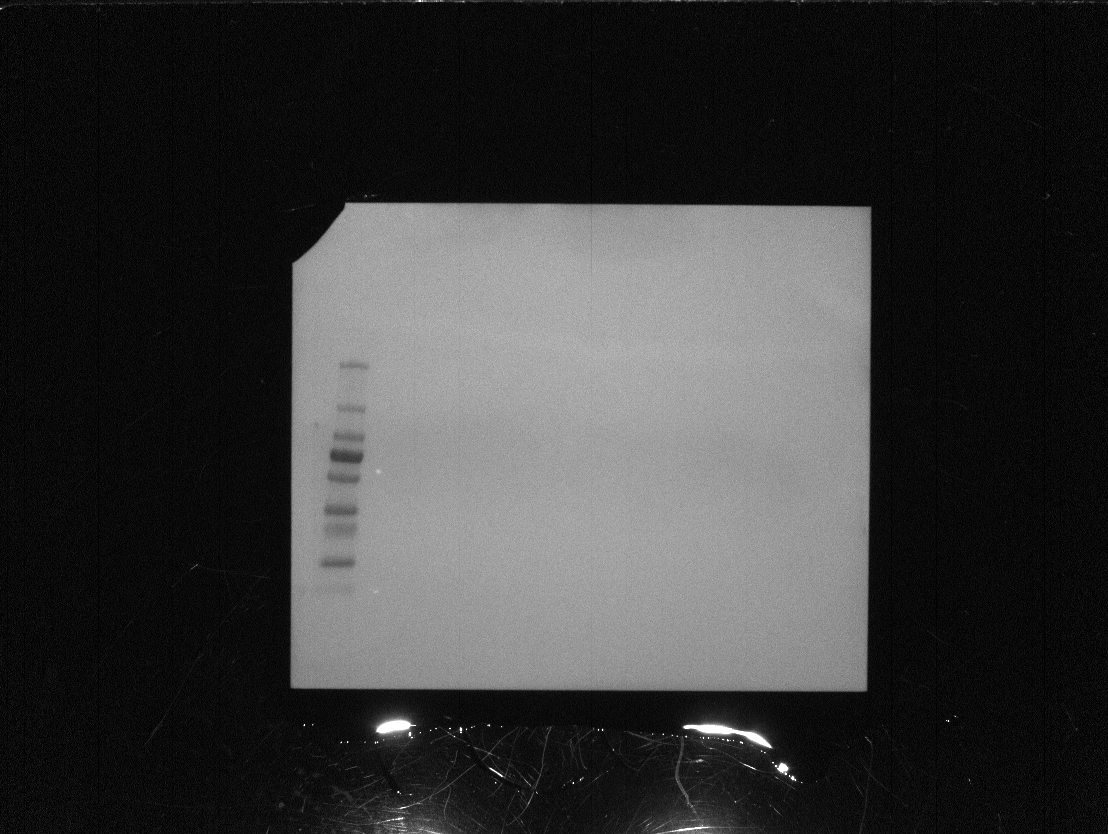

Supplement: Supplementary file 9 — Source data Fig. 2 [file 44318_2024_178_MOESM9_ESM.zip › Figure 2/2F/I/Anti-FLAG/2021-10-09_15-27-44_8bit.png]

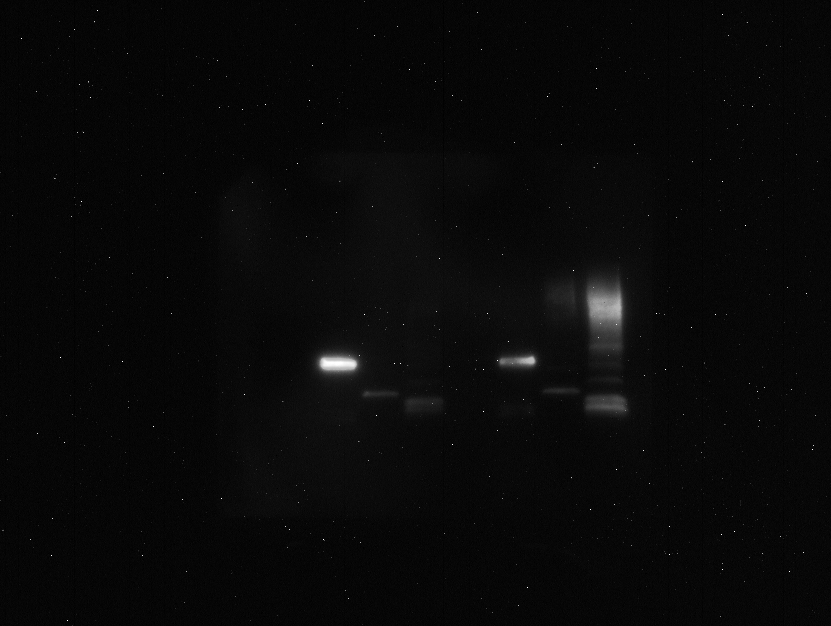

Supplement: Supplementary file 9 — Source data Fig. 2 [file 44318_2024_178_MOESM9_ESM.zip › Figure 2/2F/I/Anti-FLAG/2021-10-09_15-29-07_1_16bit.png]

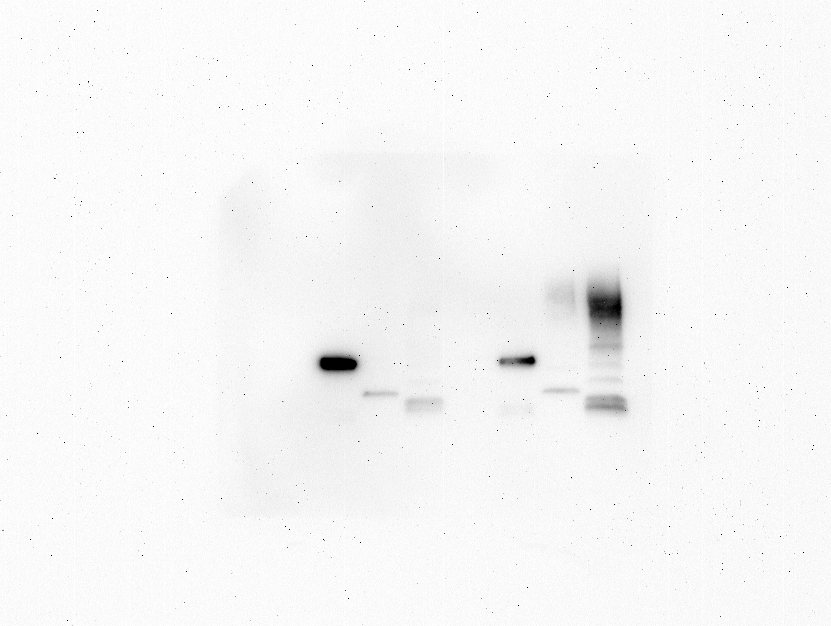

Supplement: Supplementary file 9 — Source data Fig. 2 [file 44318_2024_178_MOESM9_ESM.zip › Figure 2/2F/I/Anti-FLAG/2021-10-09_15-29-07_8bit.png]

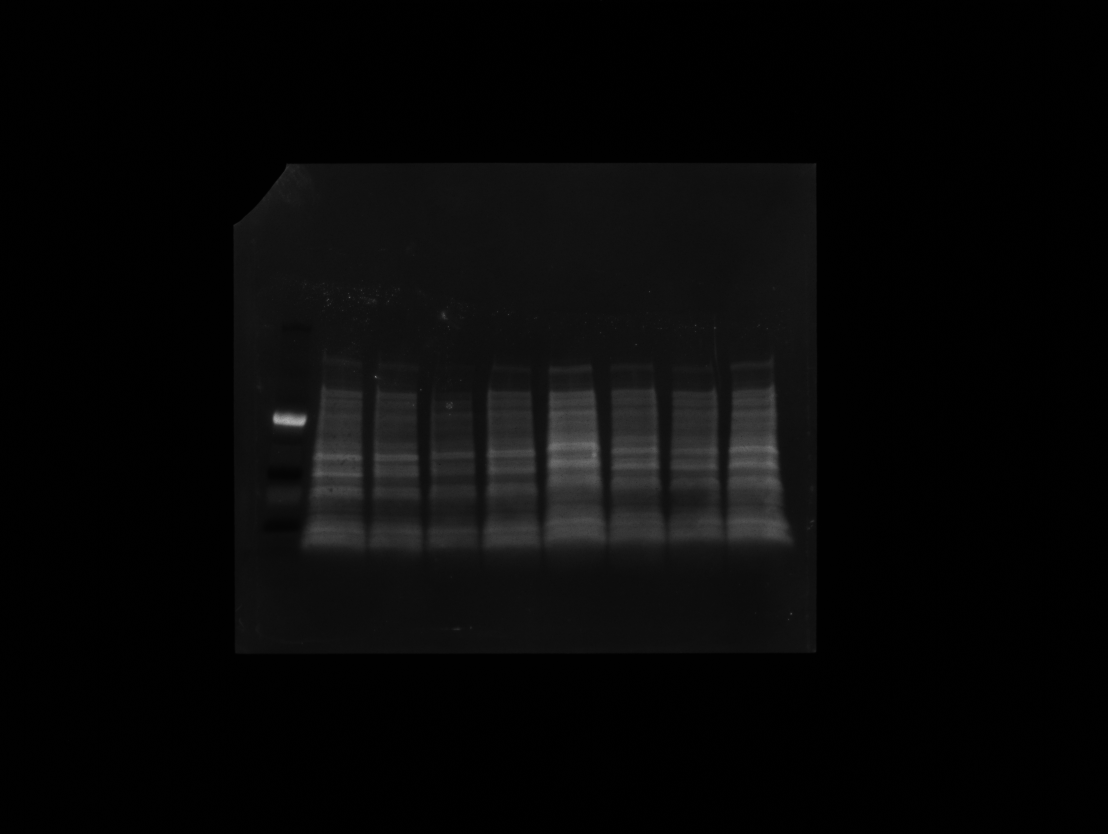

Supplement: Supplementary file 9 — Source data Fig. 2 [file 44318_2024_178_MOESM9_ESM.zip › Figure 2/2F/I/Total Protein/2021-10-08_13-21-49_1_16bit.png]

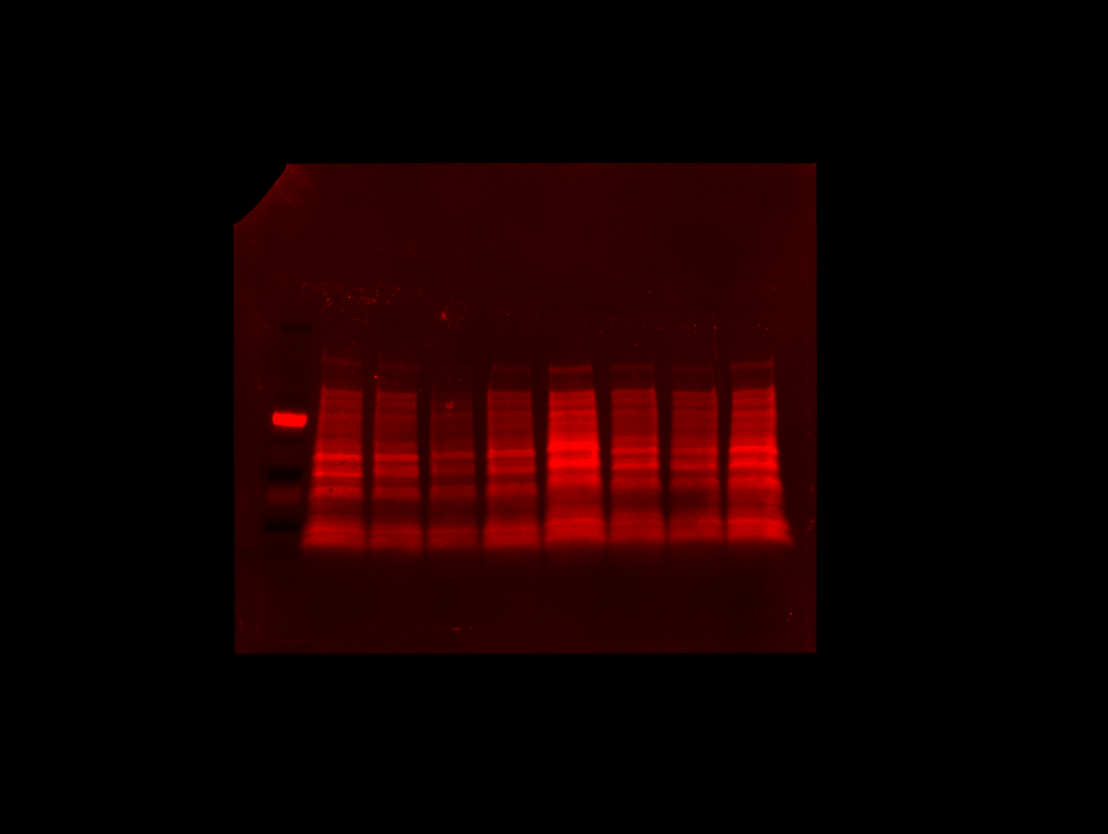

Supplement: Supplementary file 9 — Source data Fig. 2 [file 44318_2024_178_MOESM9_ESM.zip › Figure 2/2F/I/Total Protein/2021-10-08_13-21-49_8bit.png]

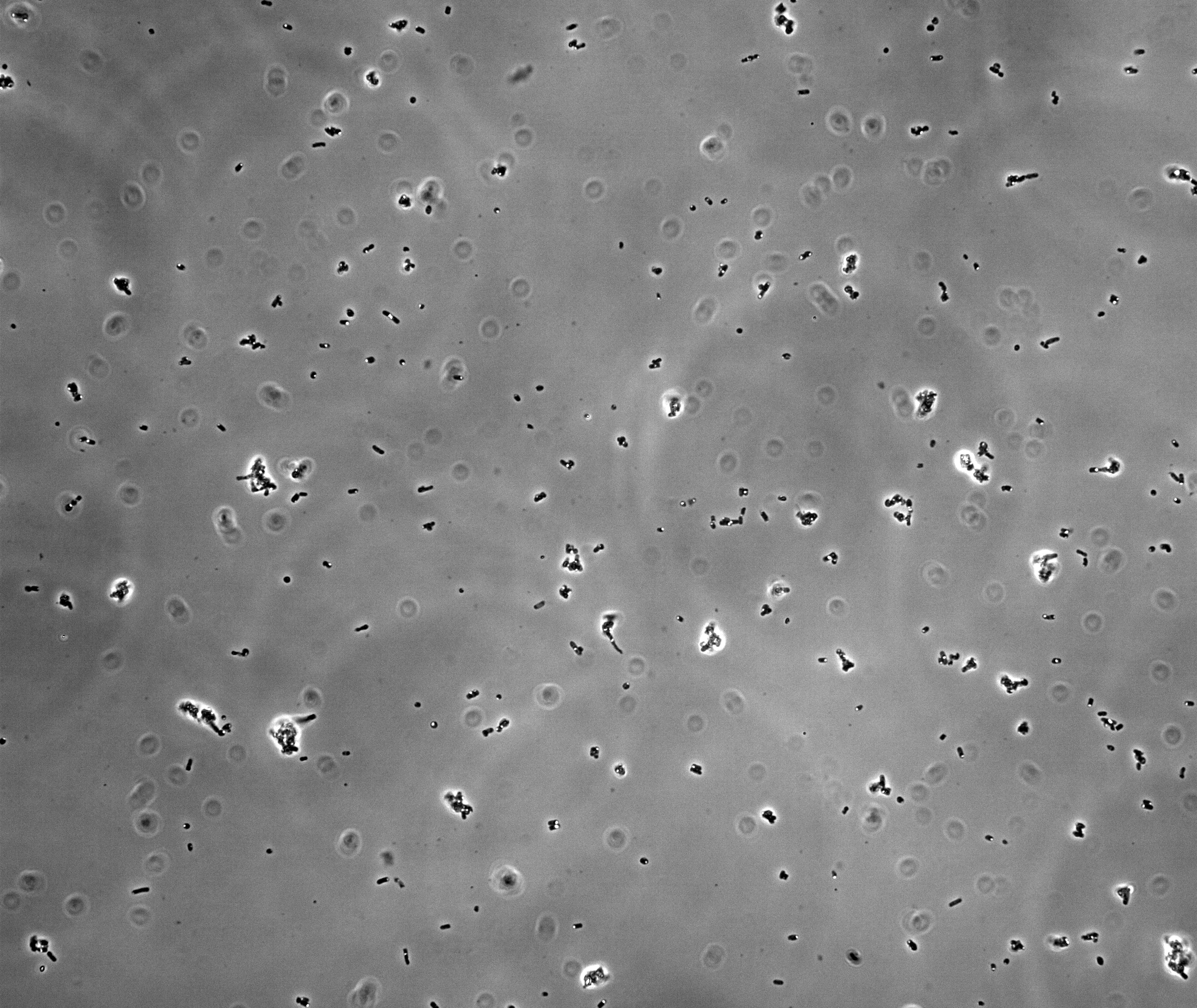

Supplement: Supplementary file 9 — Source data Fig. 2 [file 44318_2024_178_MOESM9_ESM.zip › Figure 2/2G/LGS SSM 1.nd2 - C=0-1.tif]

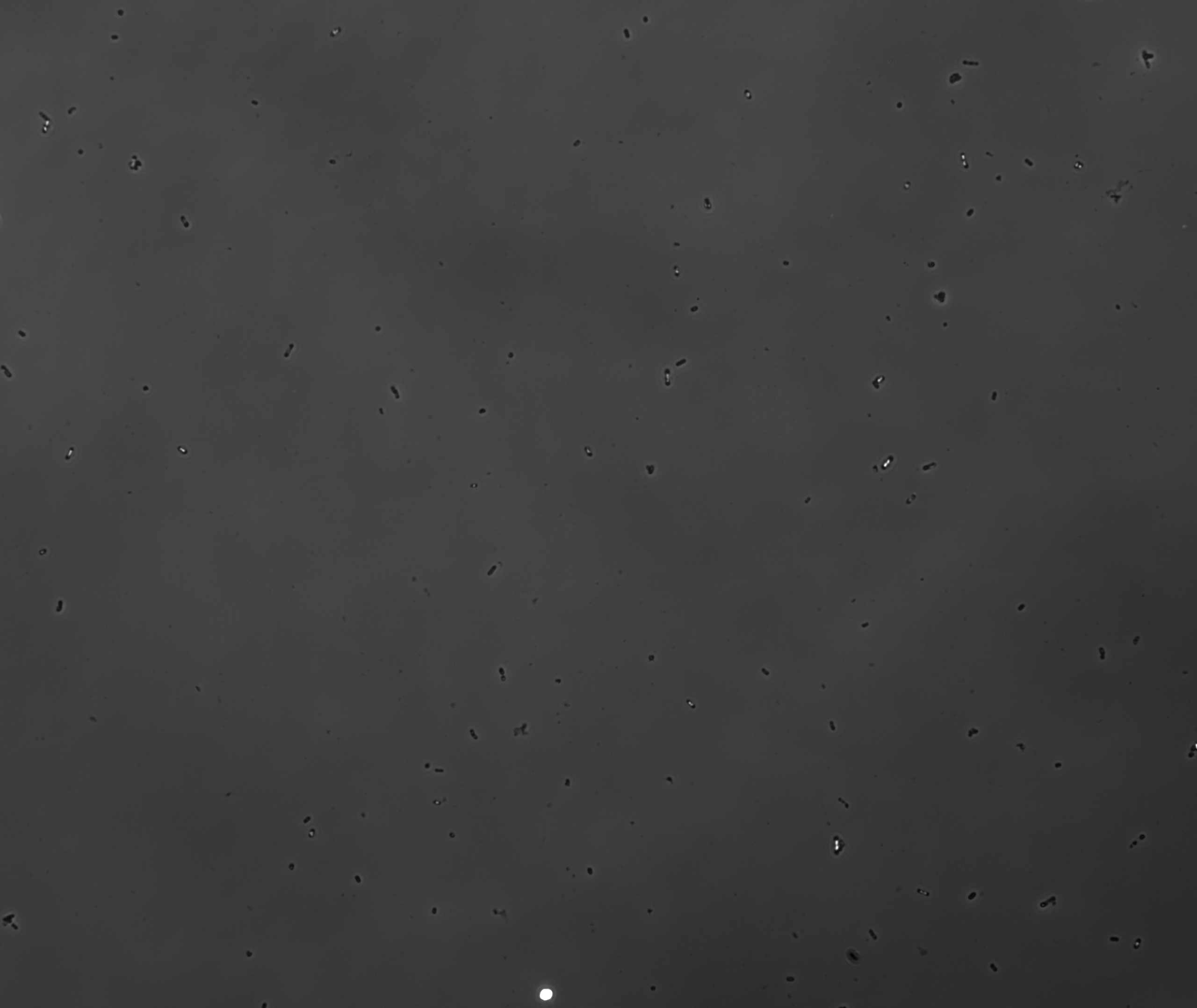

Supplement: Supplementary file 13 — Source data Fig. 6 [file 44318_2024_178_MOESM13_ESM.zip › Figure 6/6B/LGF FSM deltaF.tif]

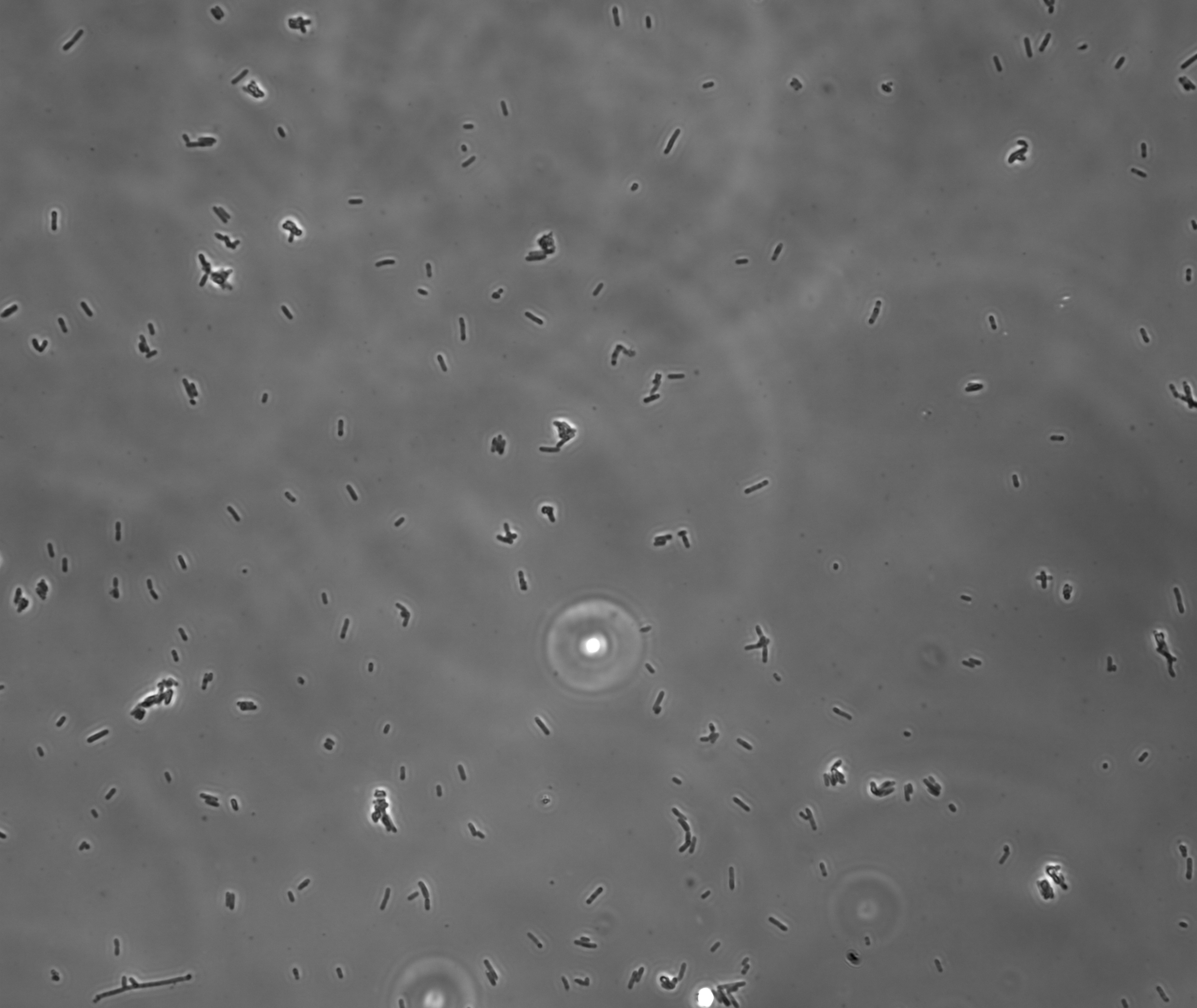

Supplement: Supplementary file 13 — Source data Fig. 6 [file 44318_2024_178_MOESM13_ESM.zip › Figure 6/6B/LGG SML deltaGL.tif]

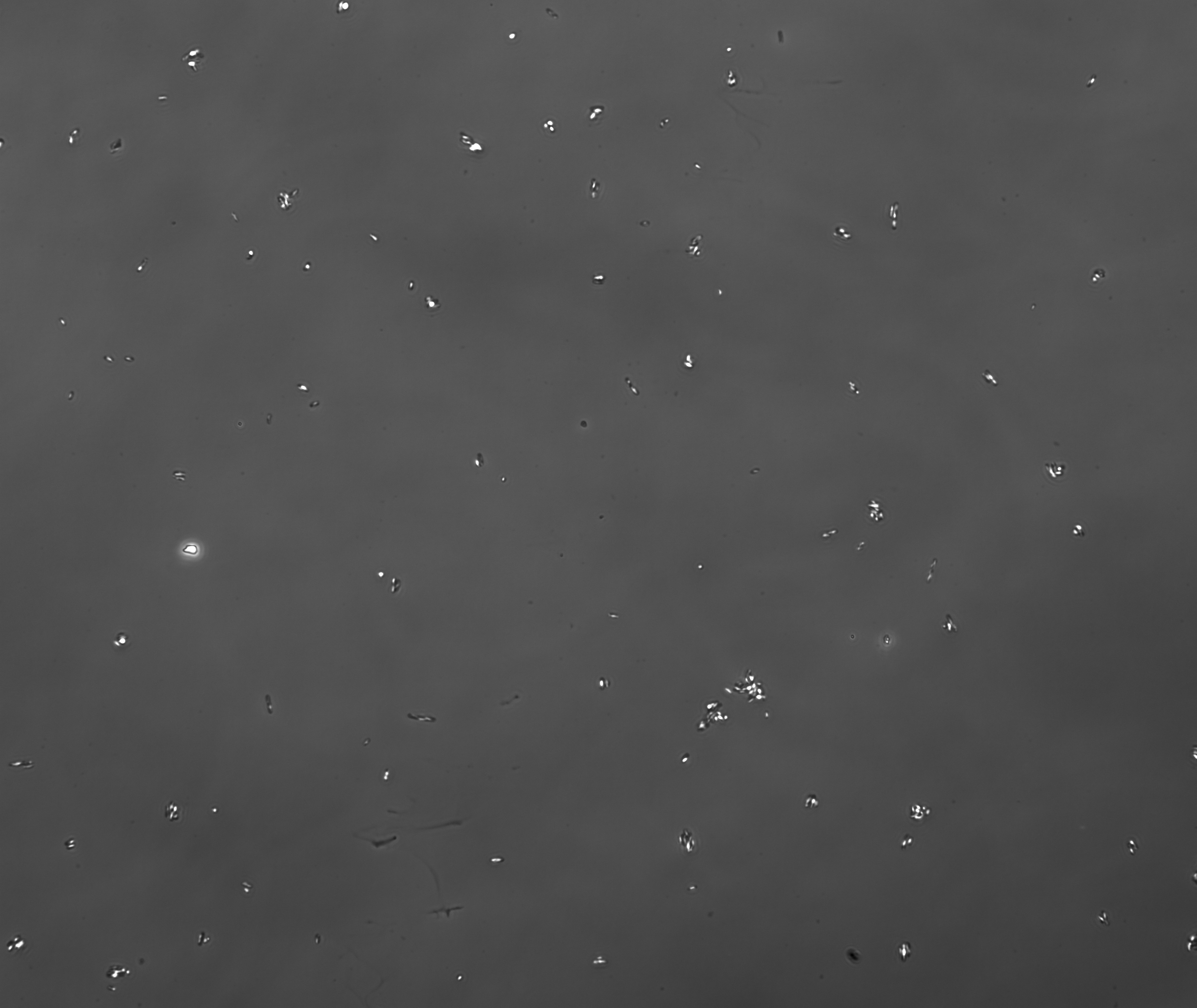

Supplement: Supplementary file 13 — Source data Fig. 6 [file 44318_2024_178_MOESM13_ESM.zip › Figure 6/6B/wt GVs.tif]

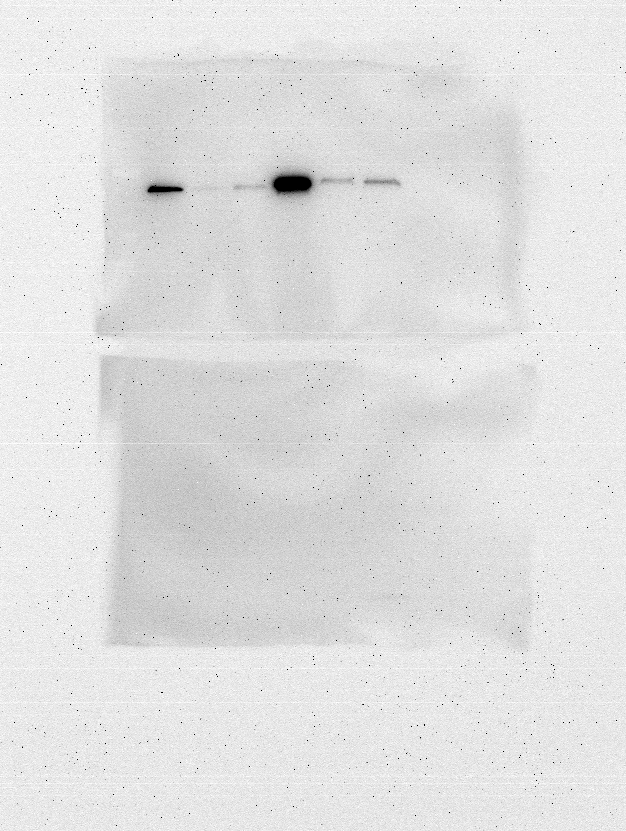

Supplement: Supplementary file 13 — Source data Fig. 6 [file 44318_2024_178_MOESM13_ESM.zip › Figure 6/6D-F/C/Anti-FLAG/2022-04-22_12-02-54_1_16bit-1.tif]

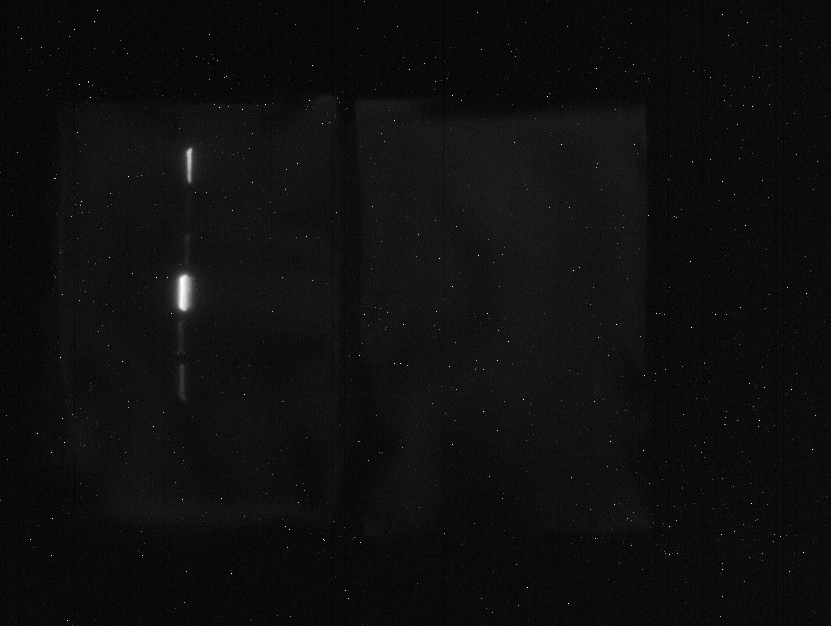

Supplement: Supplementary file 13 — Source data Fig. 6 [file 44318_2024_178_MOESM13_ESM.zip › Figure 6/6D-F/C/Anti-FLAG/2022-04-22_12-02-54_1_16bit.png]

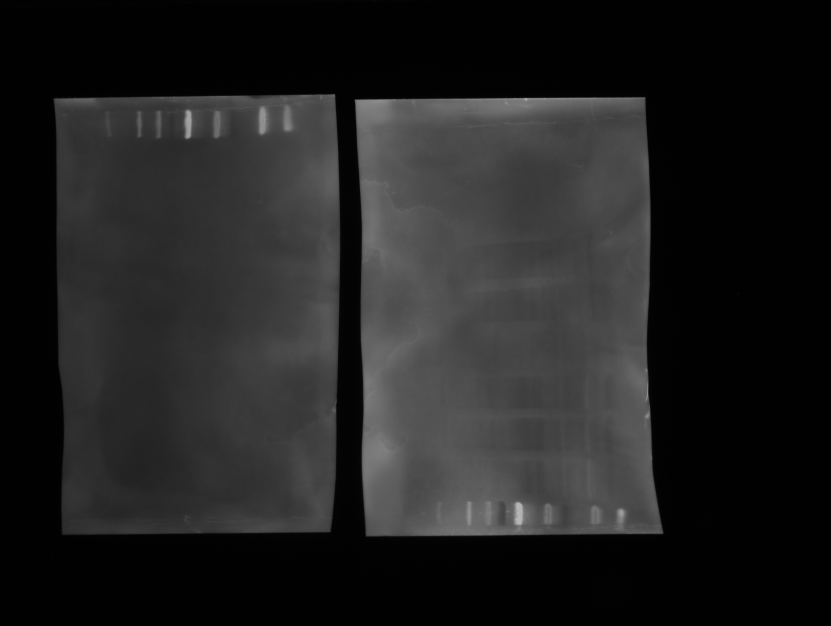

Supplement: Supplementary file 13 — Source data Fig. 6 [file 44318_2024_178_MOESM13_ESM.zip › Figure 6/6D-F/C/Anti-FLAG/2022-04-22_12-02-54_2_16bit.png]

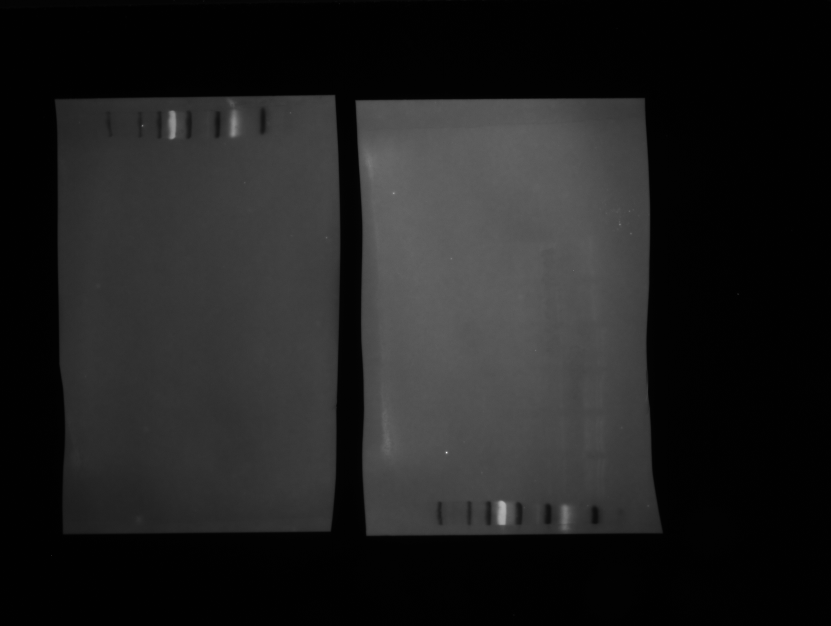

Supplement: Supplementary file 13 — Source data Fig. 6 [file 44318_2024_178_MOESM13_ESM.zip › Figure 6/6D-F/C/Anti-FLAG/2022-04-22_12-02-54_3_16bit.png]

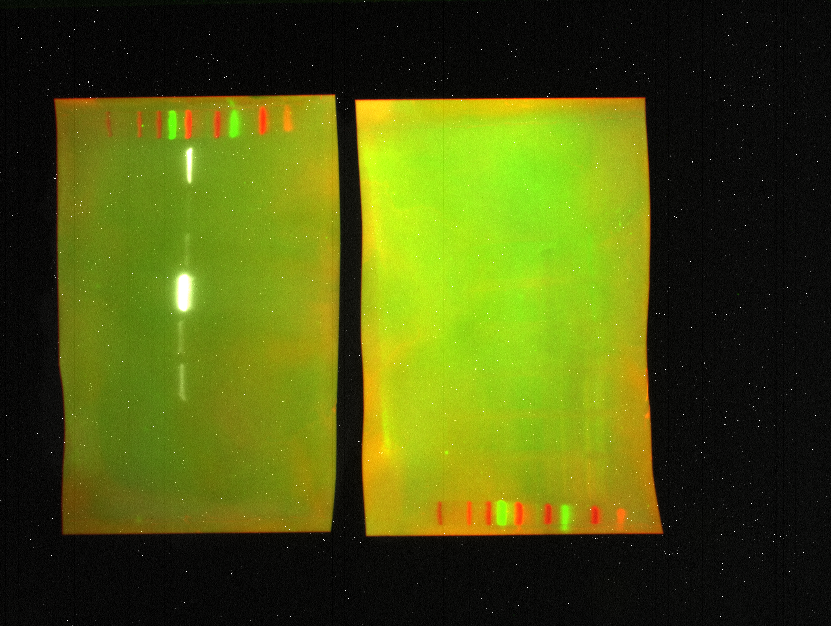

Supplement: Supplementary file 13 — Source data Fig. 6 [file 44318_2024_178_MOESM13_ESM.zip › Figure 6/6D-F/C/Anti-FLAG/2022-04-22_12-02-54_8bit.png]

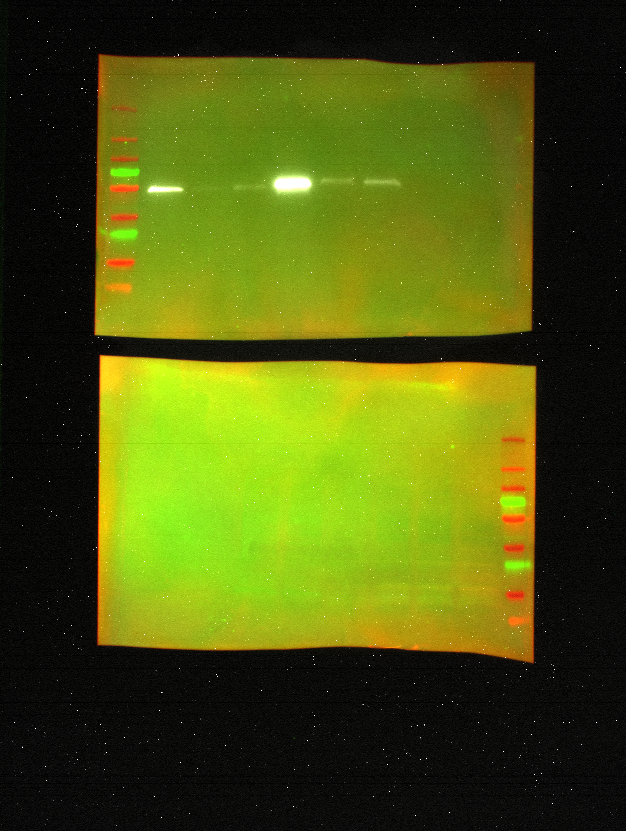

Supplement: Supplementary file 13 — Source data Fig. 6 [file 44318_2024_178_MOESM13_ESM.zip › Figure 6/6D-F/C/Anti-FLAG/2022-04-22_12-02-54_8bit.tif]

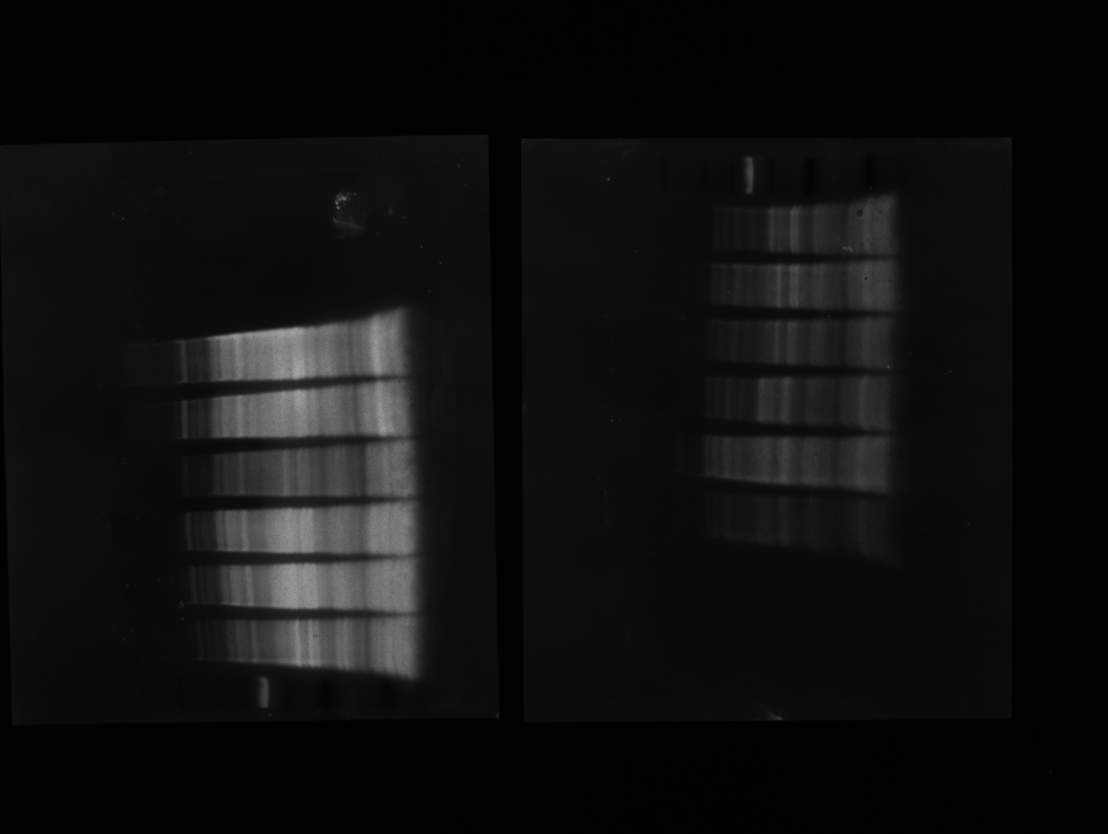

Supplement: Supplementary file 13 — Source data Fig. 6 [file 44318_2024_178_MOESM13_ESM.zip › Figure 6/6D-F/C/Total protein/2022-04-21_13-38-56_1_16bit.png]

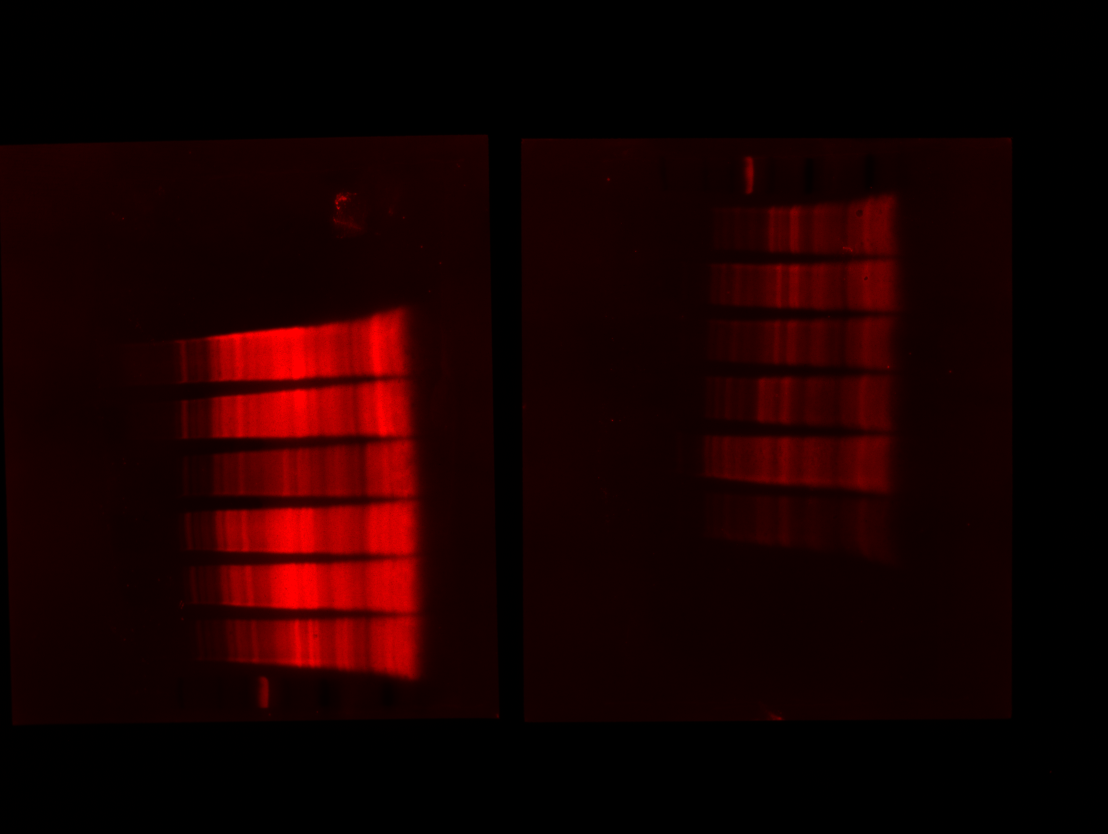

Supplement: Supplementary file 13 — Source data Fig. 6 [file 44318_2024_178_MOESM13_ESM.zip › Figure 6/6D-F/C/Total protein/2022-04-21_13-38-56_8bit.png]

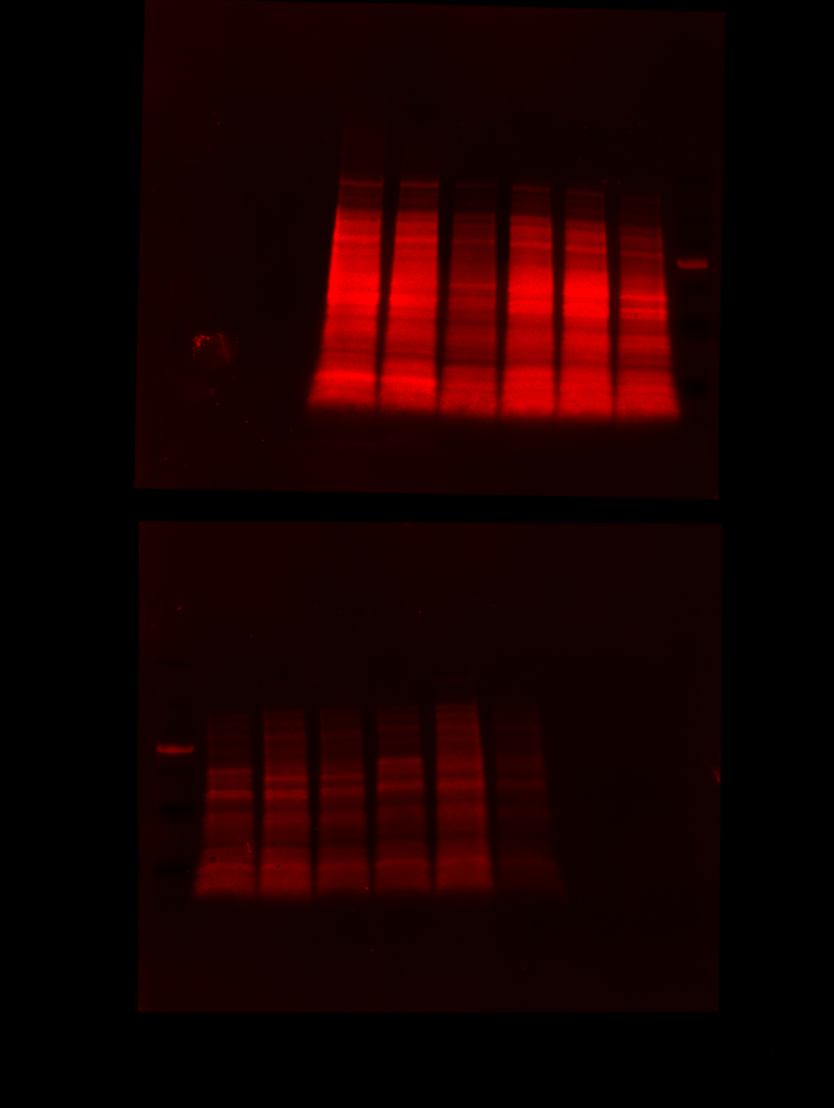

Supplement: Supplementary file 13 — Source data Fig. 6 [file 44318_2024_178_MOESM13_ESM.zip › Figure 6/6D-F/C/Total protein/2022-04-21_13-38-56_8bit.tif]

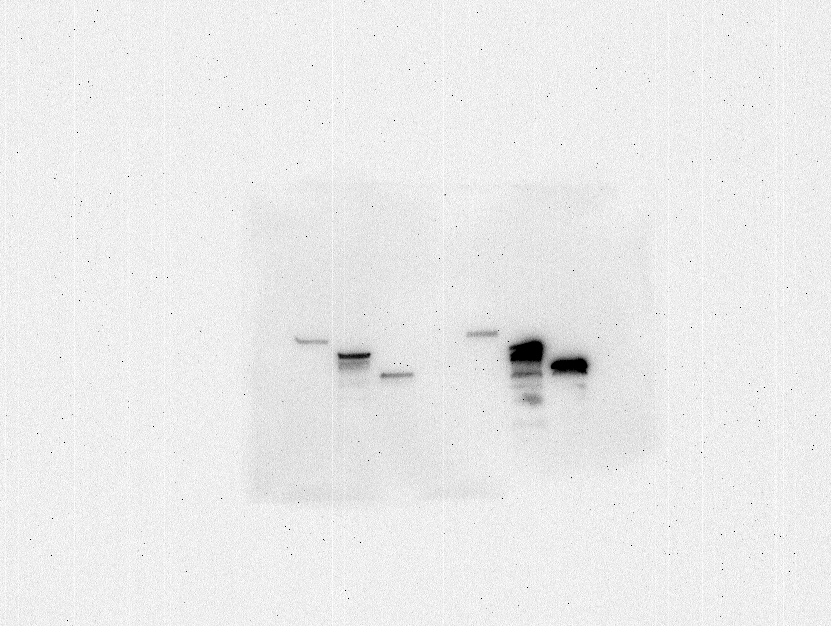

Supplement: Supplementary file 13 — Source data Fig. 6 [file 44318_2024_178_MOESM13_ESM.zip › Figure 6/6D-F/F/Anti-FLAG/2022-03-15_11-27-32_1_16bit-1.tif]

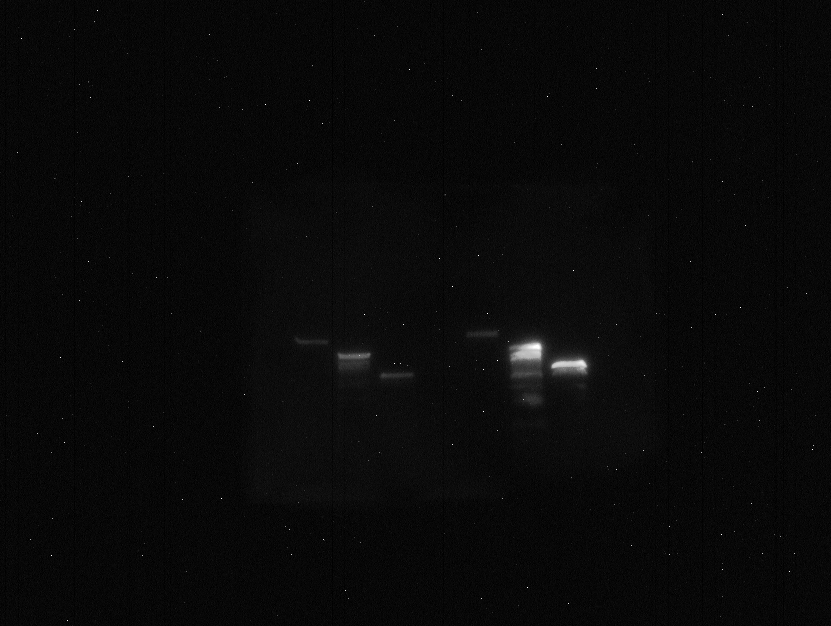

Supplement: Supplementary file 13 — Source data Fig. 6 [file 44318_2024_178_MOESM13_ESM.zip › Figure 6/6D-F/F/Anti-FLAG/2022-03-15_11-27-32_1_16bit.png]

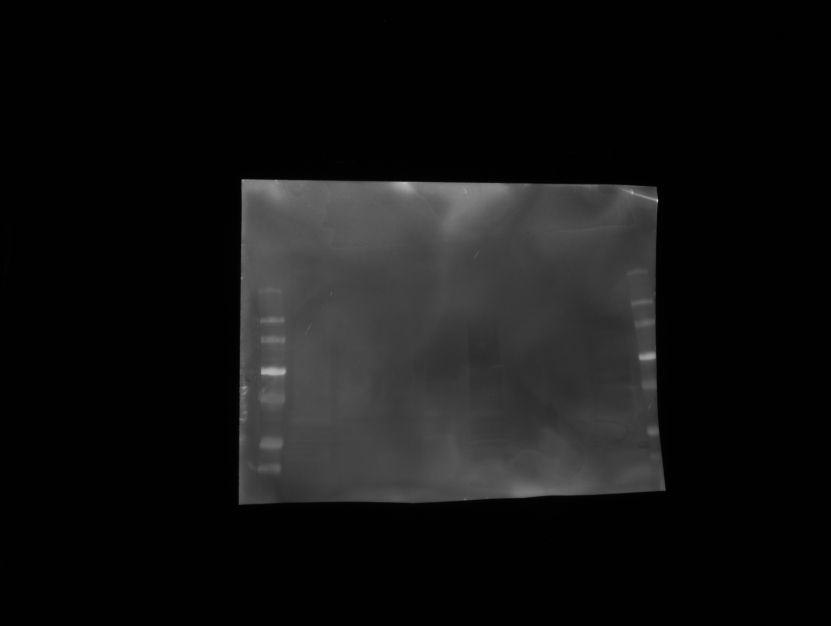

Supplement: Supplementary file 13 — Source data Fig. 6 [file 44318_2024_178_MOESM13_ESM.zip › Figure 6/6D-F/F/Anti-FLAG/2022-03-15_11-27-32_2_16bit.png]

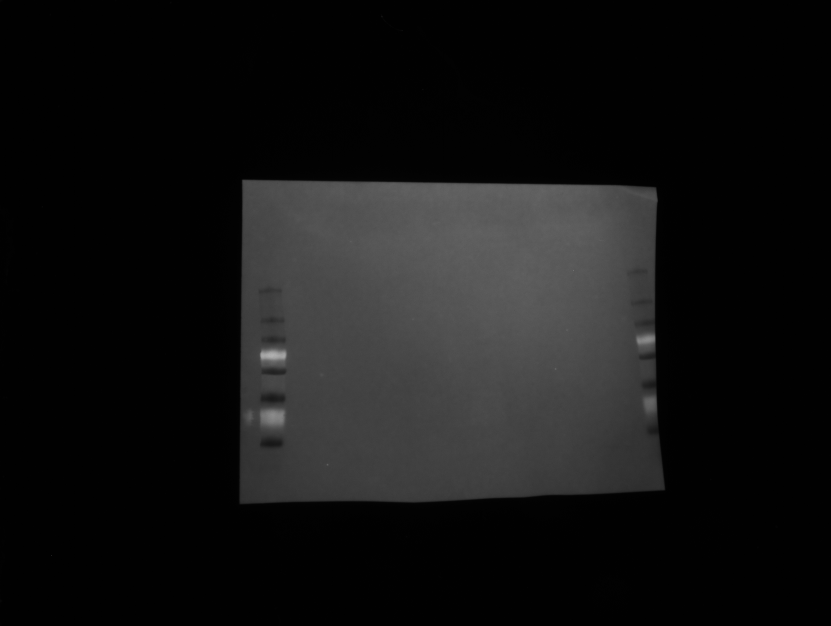

Supplement: Supplementary file 13 — Source data Fig. 6 [file 44318_2024_178_MOESM13_ESM.zip › Figure 6/6D-F/F/Anti-FLAG/2022-03-15_11-27-32_3_16bit.png]

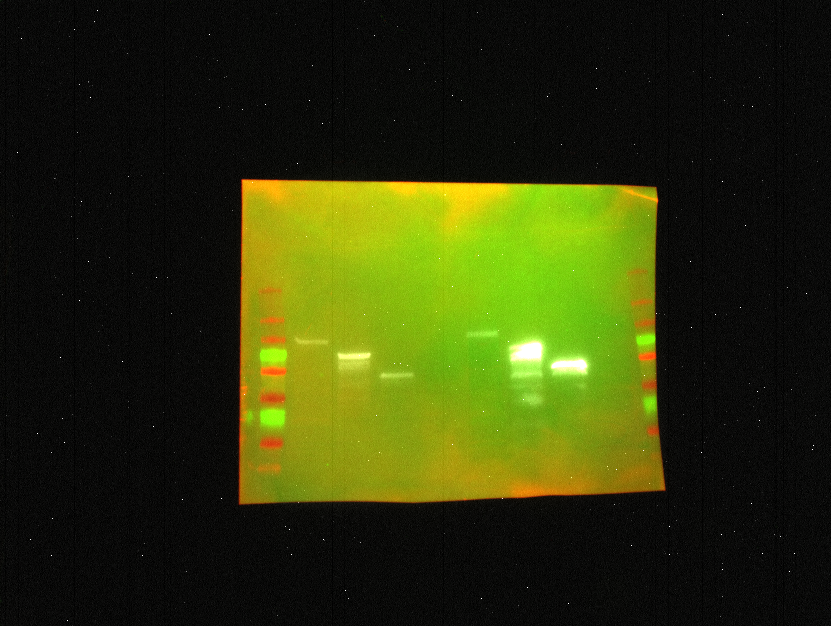

Supplement: Supplementary file 13 — Source data Fig. 6 [file 44318_2024_178_MOESM13_ESM.zip › Figure 6/6D-F/F/Anti-FLAG/2022-03-15_11-27-32_8bit.png]

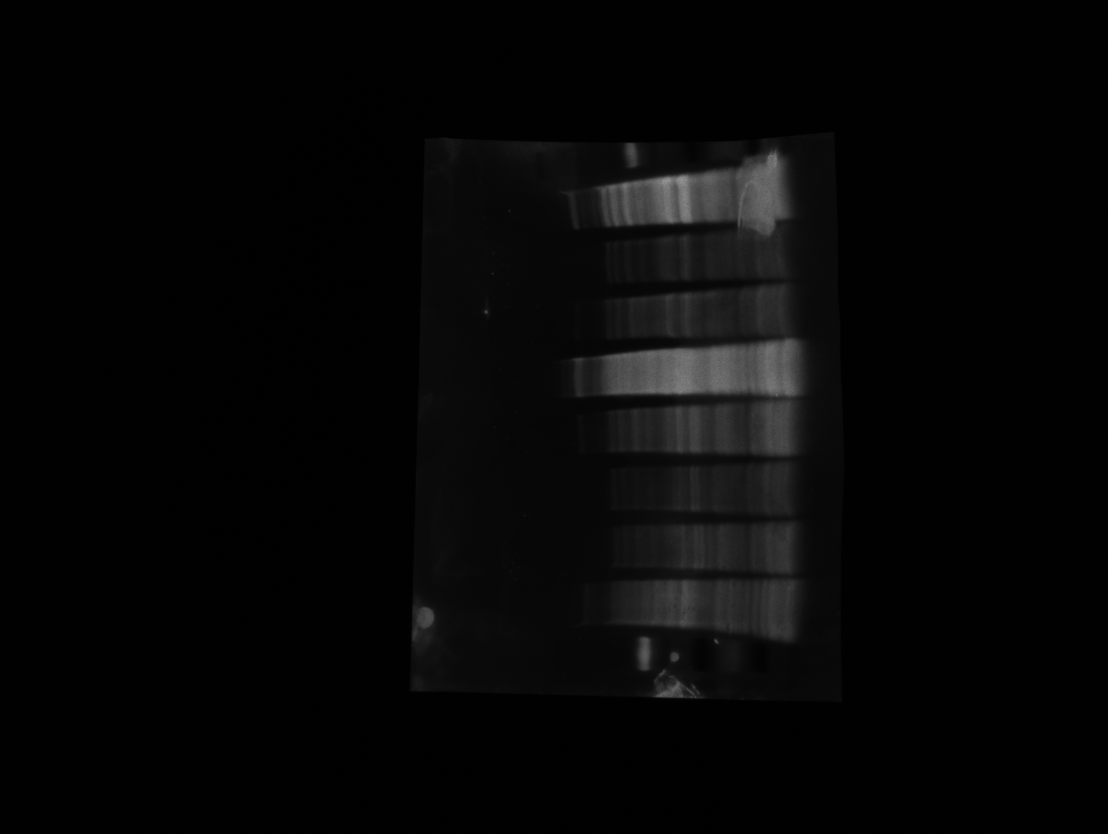

Supplement: Supplementary file 13 — Source data Fig. 6 [file 44318_2024_178_MOESM13_ESM.zip › Figure 6/6D-F/F/Total protein/2022-03-14_15-12-17_1_16bit.png]

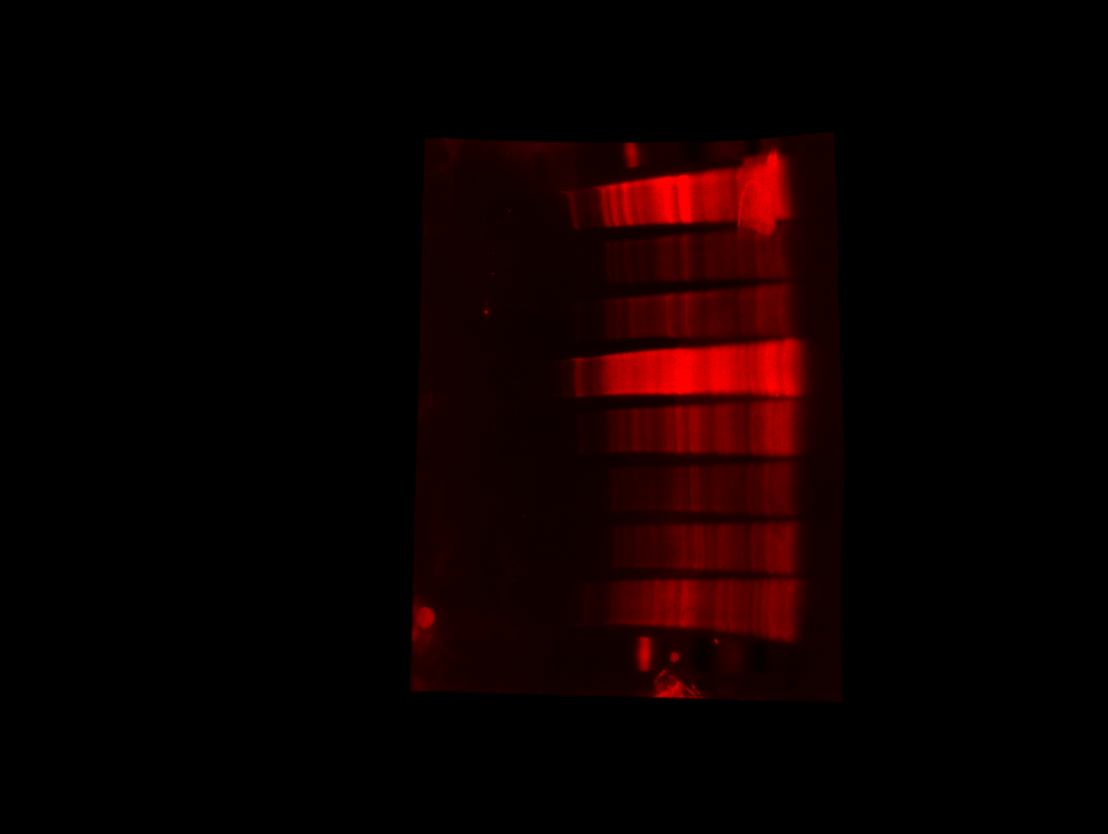

Supplement: Supplementary file 13 — Source data Fig. 6 [file 44318_2024_178_MOESM13_ESM.zip › Figure 6/6D-F/F/Total protein/2022-03-14_15-12-17_8bit.png]

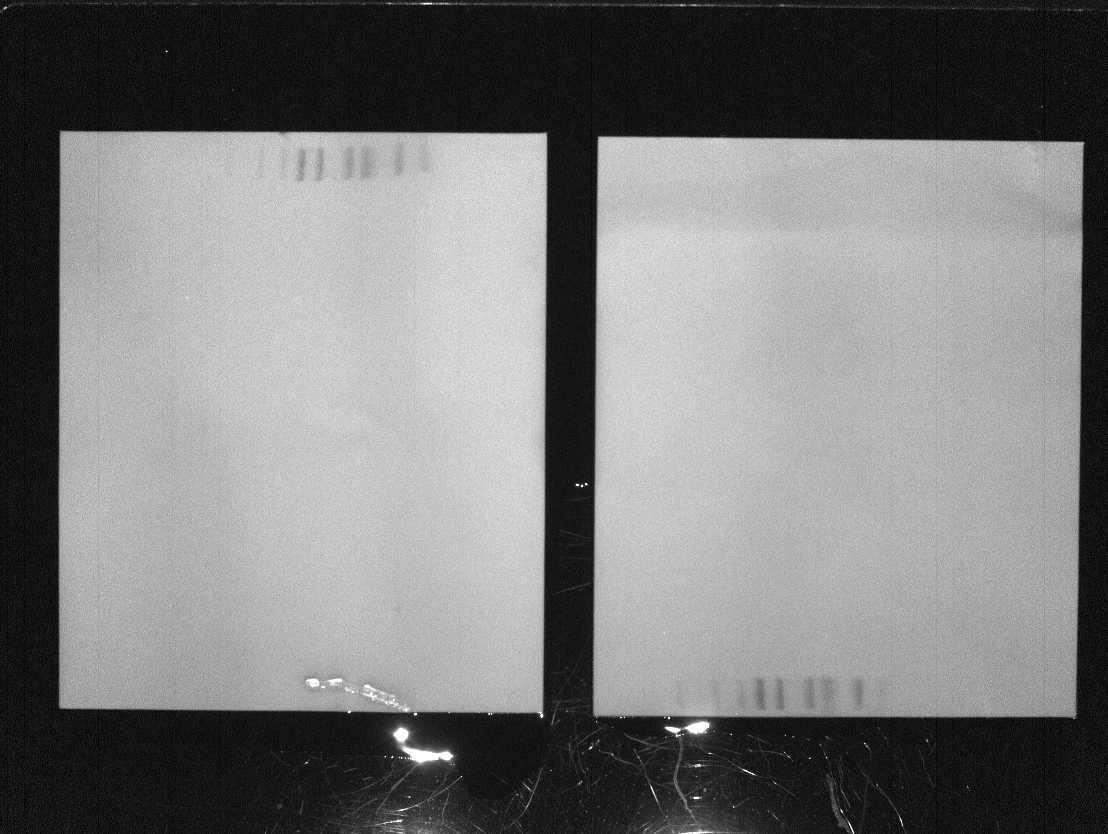

Supplement: Supplementary file 15 — Source data Fig. S4 [file 44318_2024_178_MOESM15_ESM.zip › Appendix Figure S4/A, B/Anti-FLAG/2021-10-16_17-10-40_1_16bit.png]

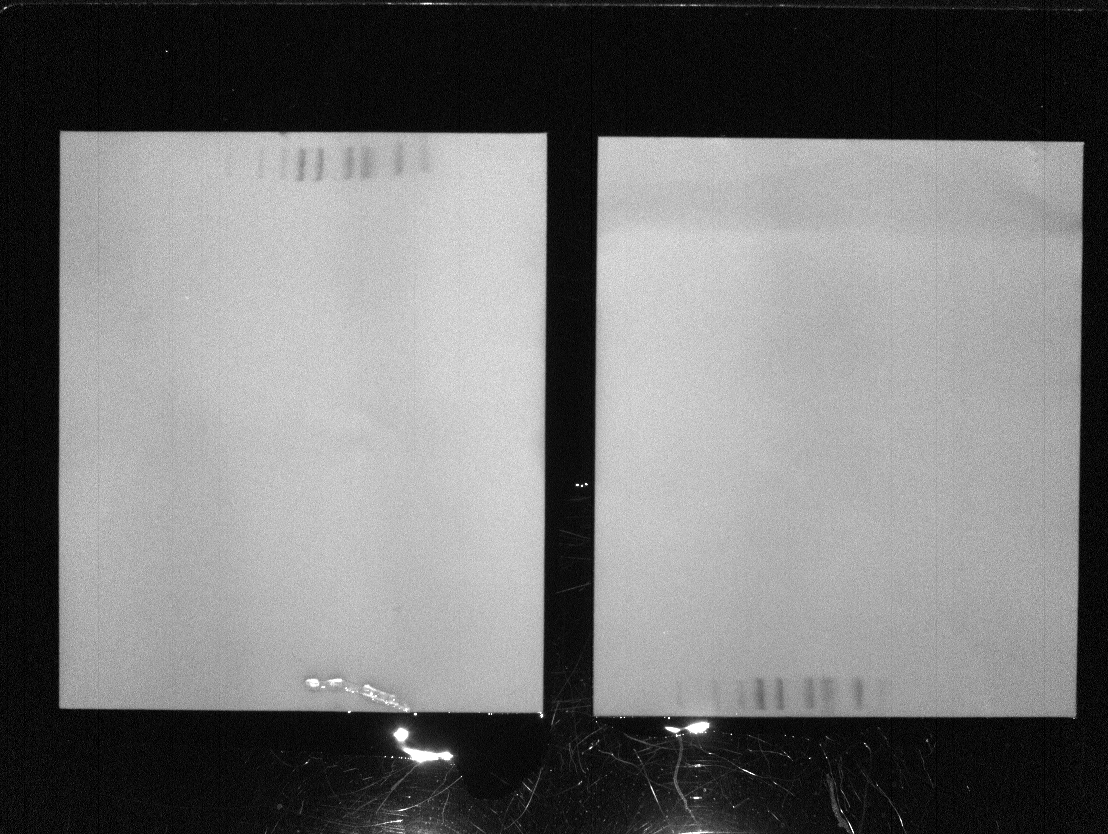

Supplement: Supplementary file 15 — Source data Fig. S4 [file 44318_2024_178_MOESM15_ESM.zip › Appendix Figure S4/A, B/Anti-FLAG/2021-10-16_17-10-40_8bit.png]

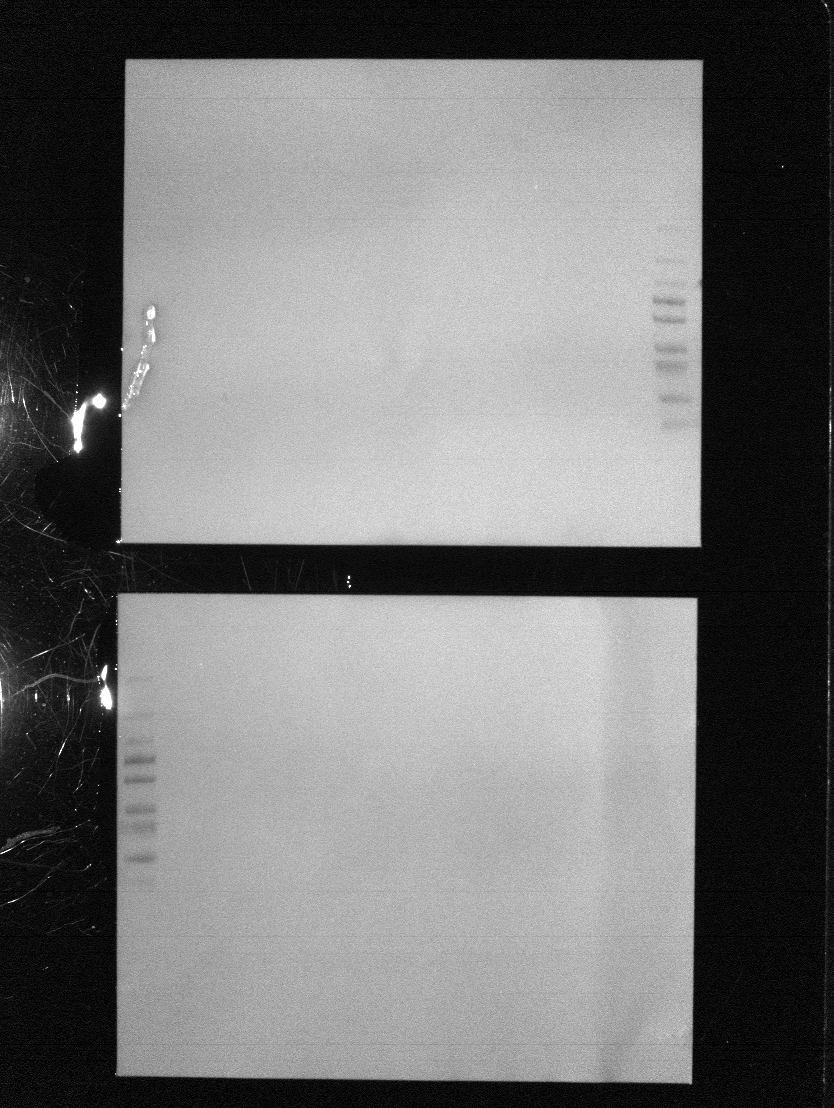

Supplement: Supplementary file 15 — Source data Fig. S4 [file 44318_2024_178_MOESM15_ESM.zip › Appendix Figure S4/A, B/Anti-FLAG/2021-10-16_17-10-40_8bit.tif]

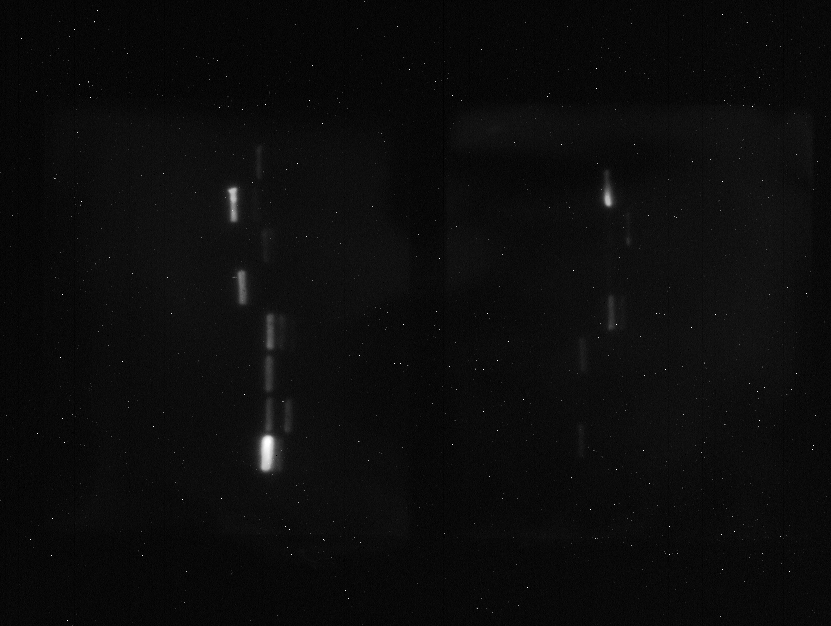

Supplement: Supplementary file 15 — Source data Fig. S4 [file 44318_2024_178_MOESM15_ESM.zip › Appendix Figure S4/A, B/Anti-FLAG/2021-10-16_17-12-11_1_16bit.png]

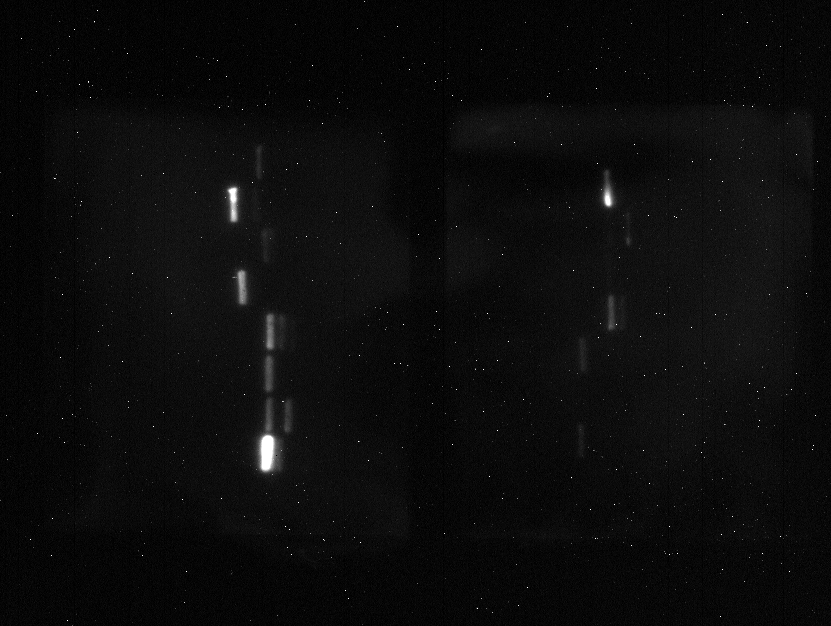

Supplement: Supplementary file 15 — Source data Fig. S4 [file 44318_2024_178_MOESM15_ESM.zip › Appendix Figure S4/A, B/Anti-FLAG/2021-10-16_17-12-11_8bit.png]

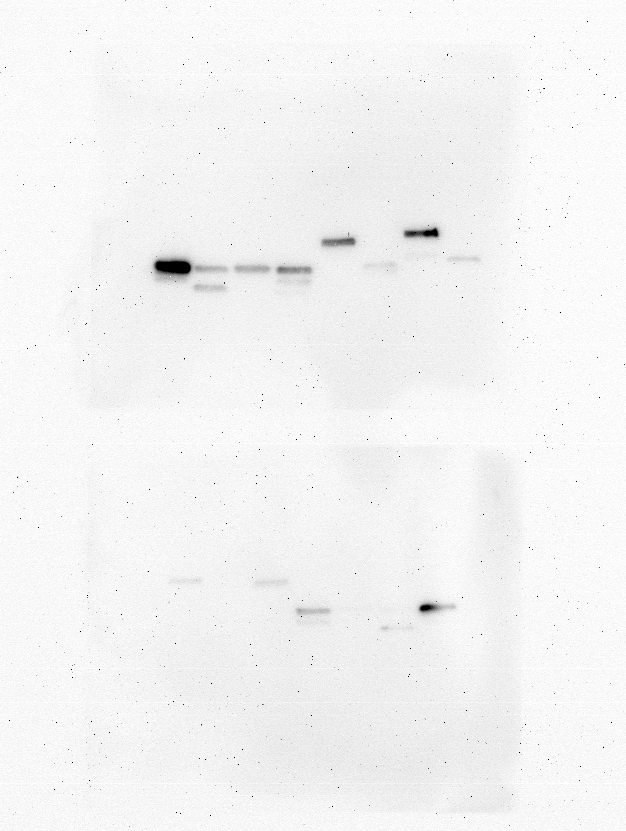

Supplement: Supplementary file 15 — Source data Fig. S4 [file 44318_2024_178_MOESM15_ESM.zip › Appendix Figure S4/A, B/Anti-FLAG/2021-10-16_17-12-11_8bit.tif]

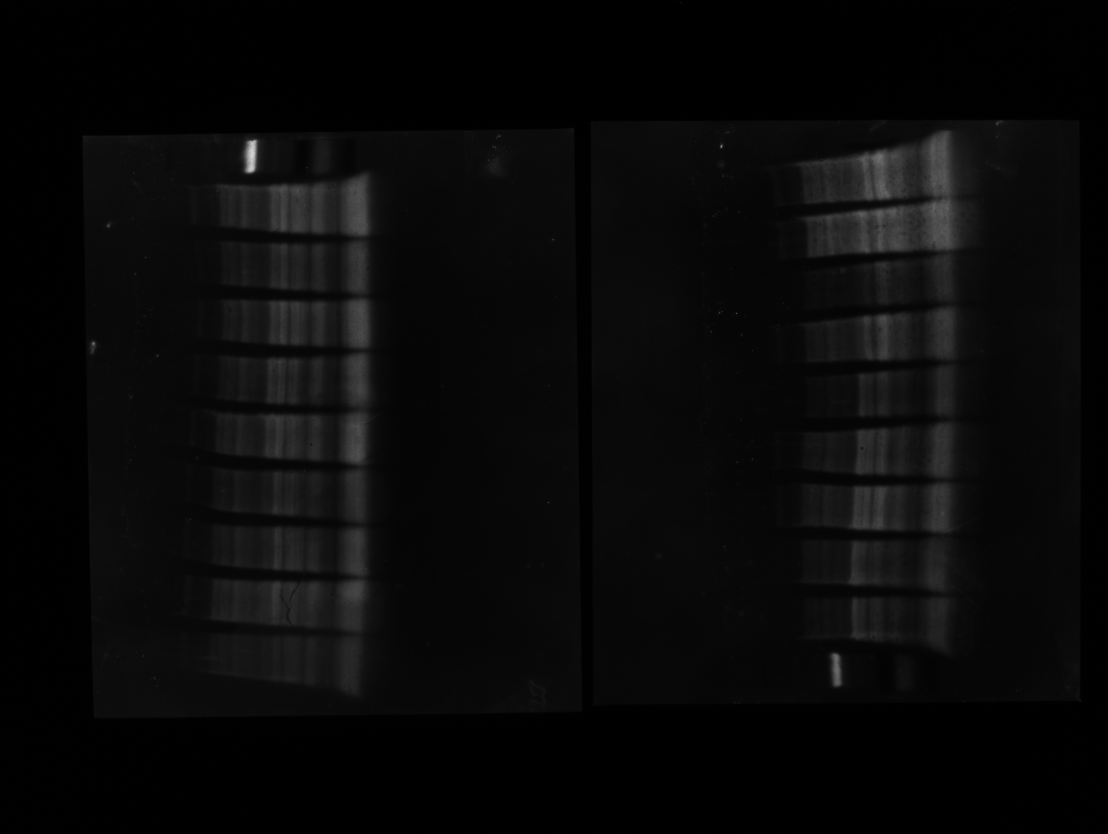

Supplement: Supplementary file 15 — Source data Fig. S4 [file 44318_2024_178_MOESM15_ESM.zip › Appendix Figure S4/A, B/Total protein/2021-10-15_18-07-16_1_16bit.png]

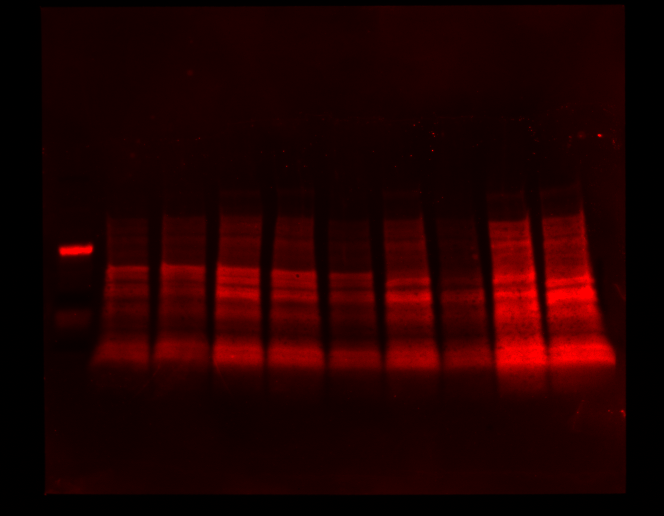

Supplement: Supplementary file 15 — Source data Fig. S4 [file 44318_2024_178_MOESM15_ESM.zip › Appendix Figure S4/A, B/Total protein/2021-10-15_18-07-16_8bit bottom.png]

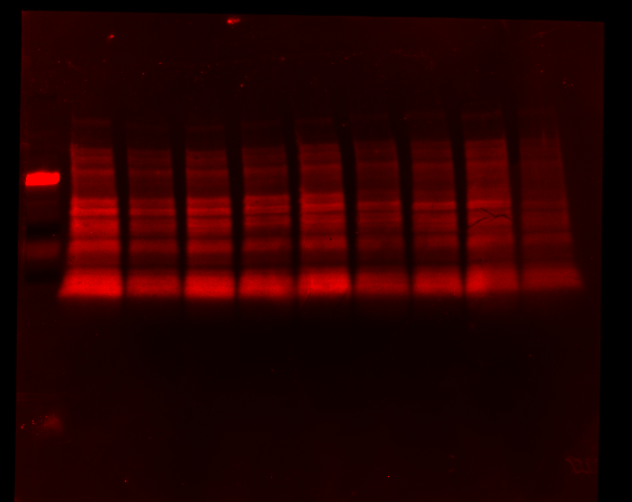

Supplement: Supplementary file 15 — Source data Fig. S4 [file 44318_2024_178_MOESM15_ESM.zip › Appendix Figure S4/A, B/Total protein/2021-10-15_18-07-16_8bit-1 top.png]

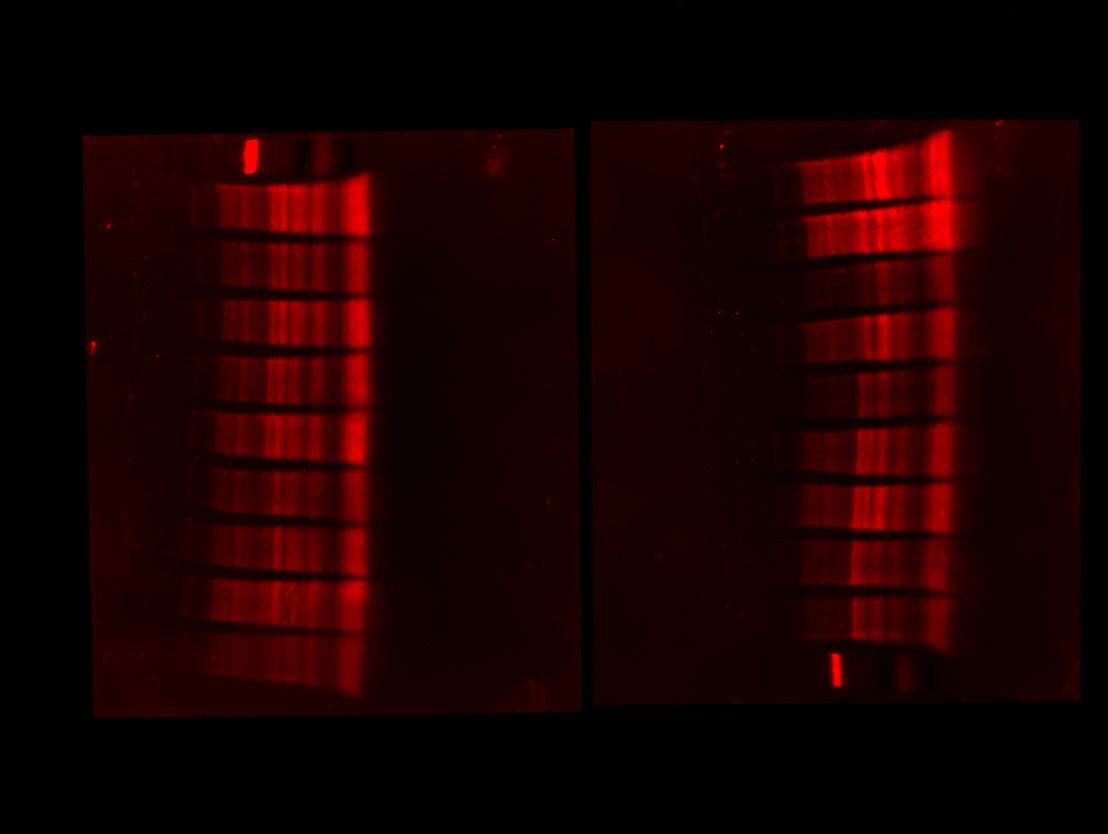

Supplement: Supplementary file 15 — Source data Fig. S4 [file 44318_2024_178_MOESM15_ESM.zip › Appendix Figure S4/A, B/Total protein/2021-10-15_18-07-16_8bit.png]

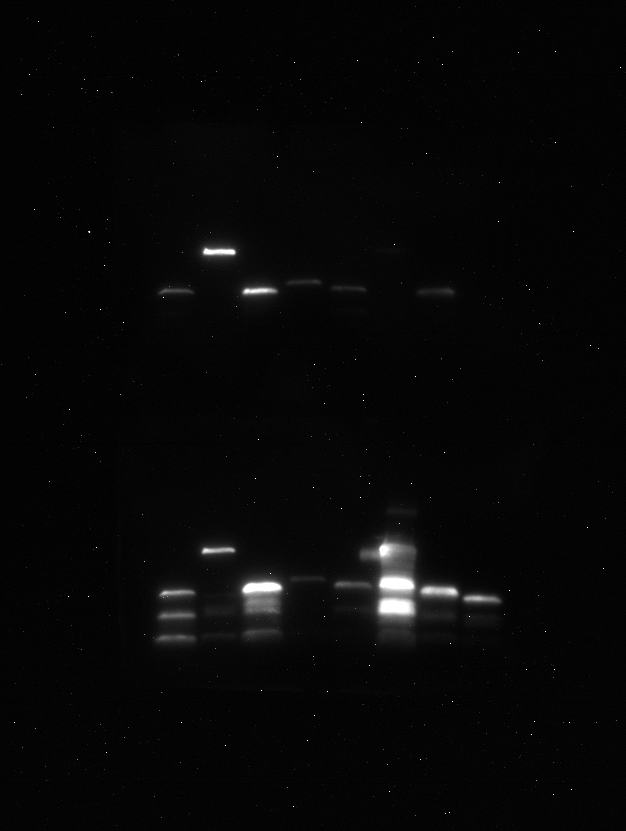

Supplement: Supplementary file 15 — Source data Fig. S4 [file 44318_2024_178_MOESM15_ESM.zip › Appendix Figure S4/D, E/Anti-FLAG/2021-12-21_11-25-45_1_16bit-1.png]

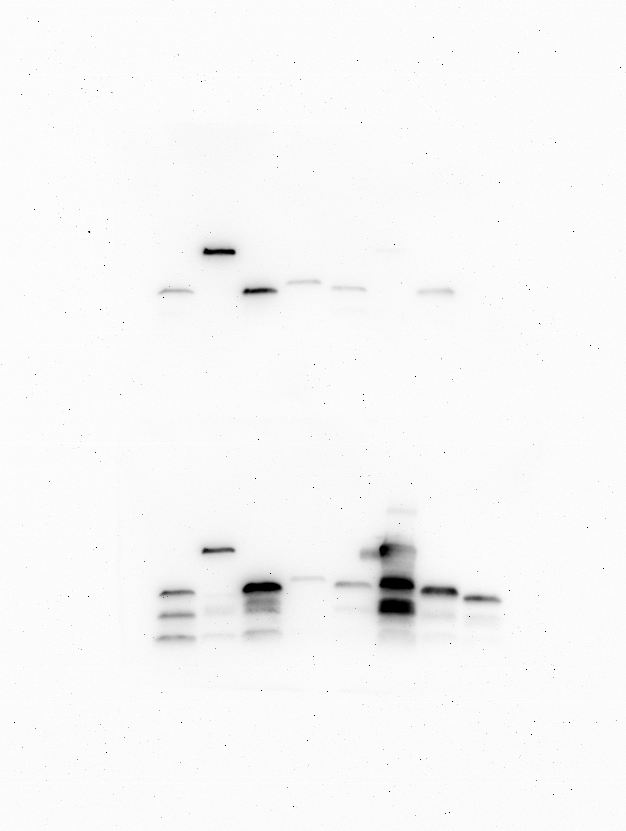

Supplement: Supplementary file 15 — Source data Fig. S4 [file 44318_2024_178_MOESM15_ESM.zip › Appendix Figure S4/D, E/Anti-FLAG/2021-12-21_11-25-45_1_16bit-1.tif]

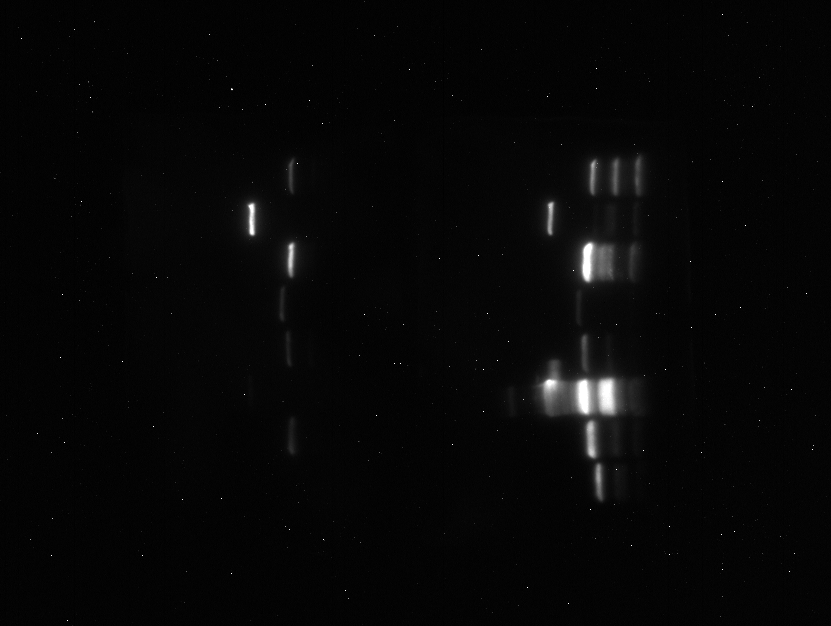

Supplement: Supplementary file 15 — Source data Fig. S4 [file 44318_2024_178_MOESM15_ESM.zip › Appendix Figure S4/D, E/Anti-FLAG/2021-12-21_11-25-45_1_16bit.png]

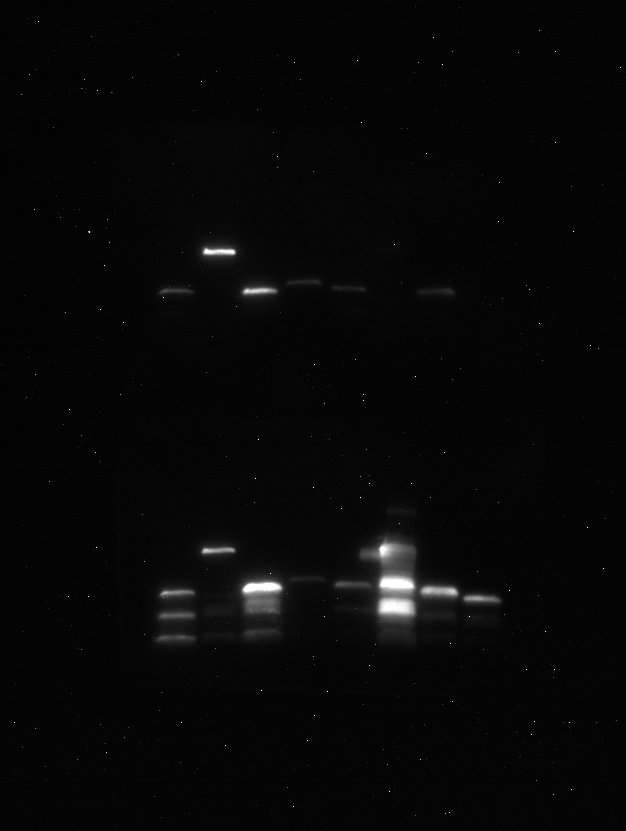

Supplement: Supplementary file 15 — Source data Fig. S4 [file 44318_2024_178_MOESM15_ESM.zip › Appendix Figure S4/D, E/Anti-FLAG/2021-12-21_11-25-45_1_16bit.tif]

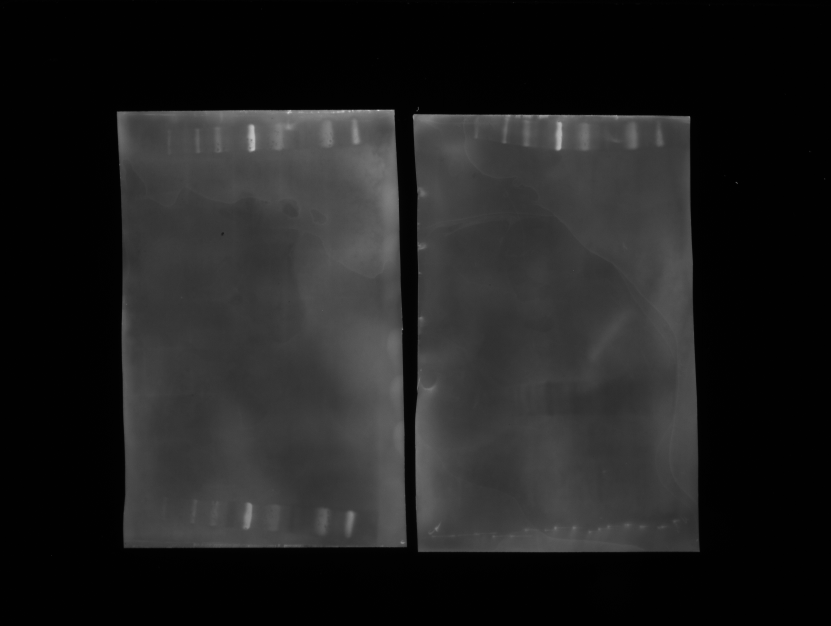

Supplement: Supplementary file 15 — Source data Fig. S4 [file 44318_2024_178_MOESM15_ESM.zip › Appendix Figure S4/D, E/Anti-FLAG/2021-12-21_11-25-45_2_16bit.png]

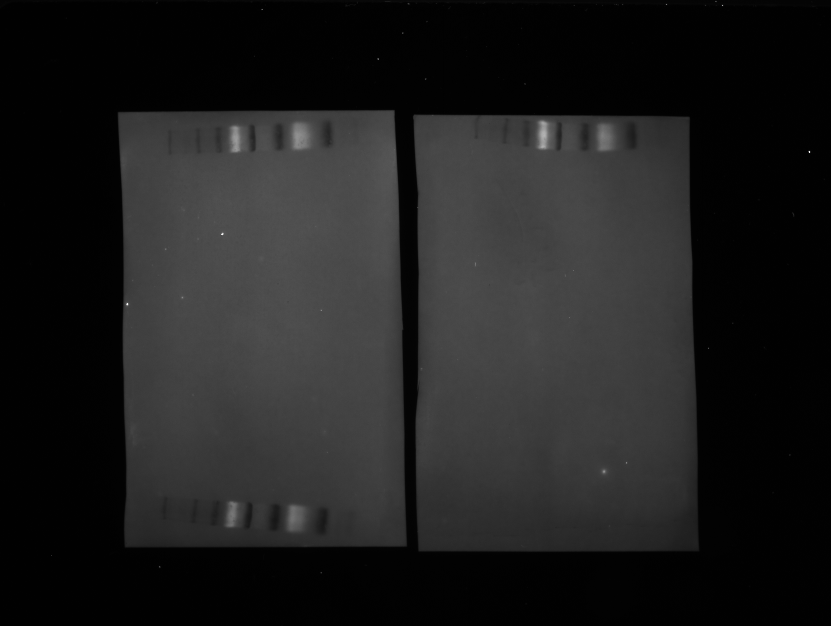

Supplement: Supplementary file 15 — Source data Fig. S4 [file 44318_2024_178_MOESM15_ESM.zip › Appendix Figure S4/D, E/Anti-FLAG/2021-12-21_11-25-45_3_16bit.png]

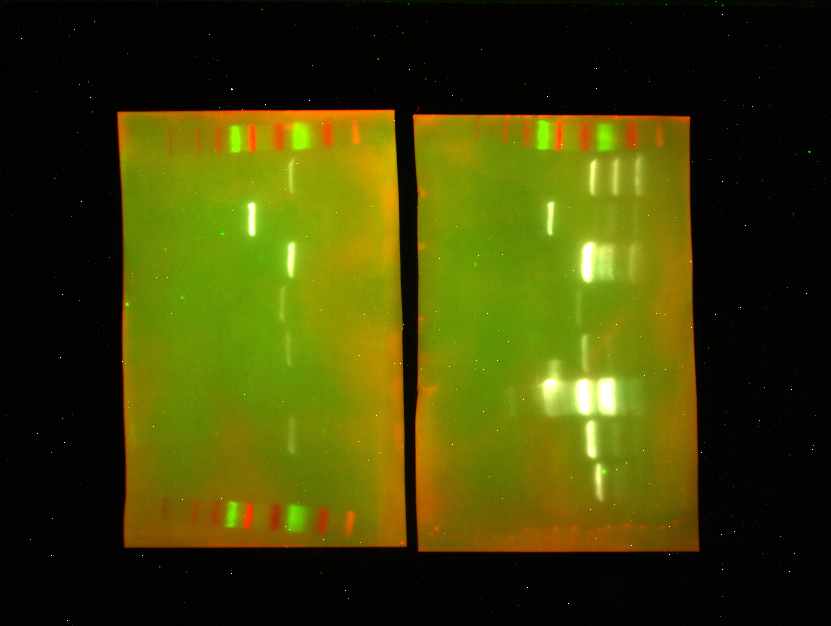

Supplement: Supplementary file 15 — Source data Fig. S4 [file 44318_2024_178_MOESM15_ESM.zip › Appendix Figure S4/D, E/Anti-FLAG/2021-12-21_11-25-45_8bit.png]

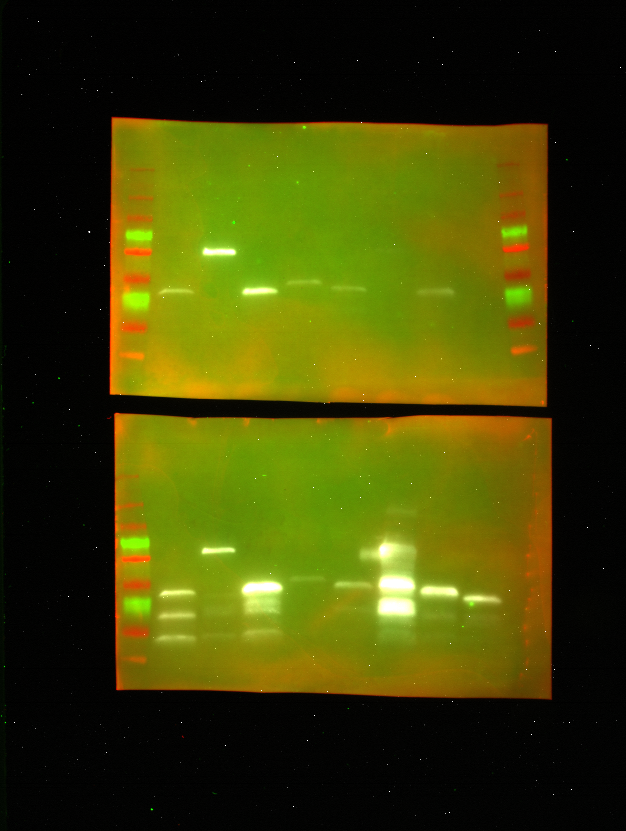

Supplement: Supplementary file 15 — Source data Fig. S4 [file 44318_2024_178_MOESM15_ESM.zip › Appendix Figure S4/D, E/Anti-FLAG/2021-12-21_11-25-45_8bit.tif]

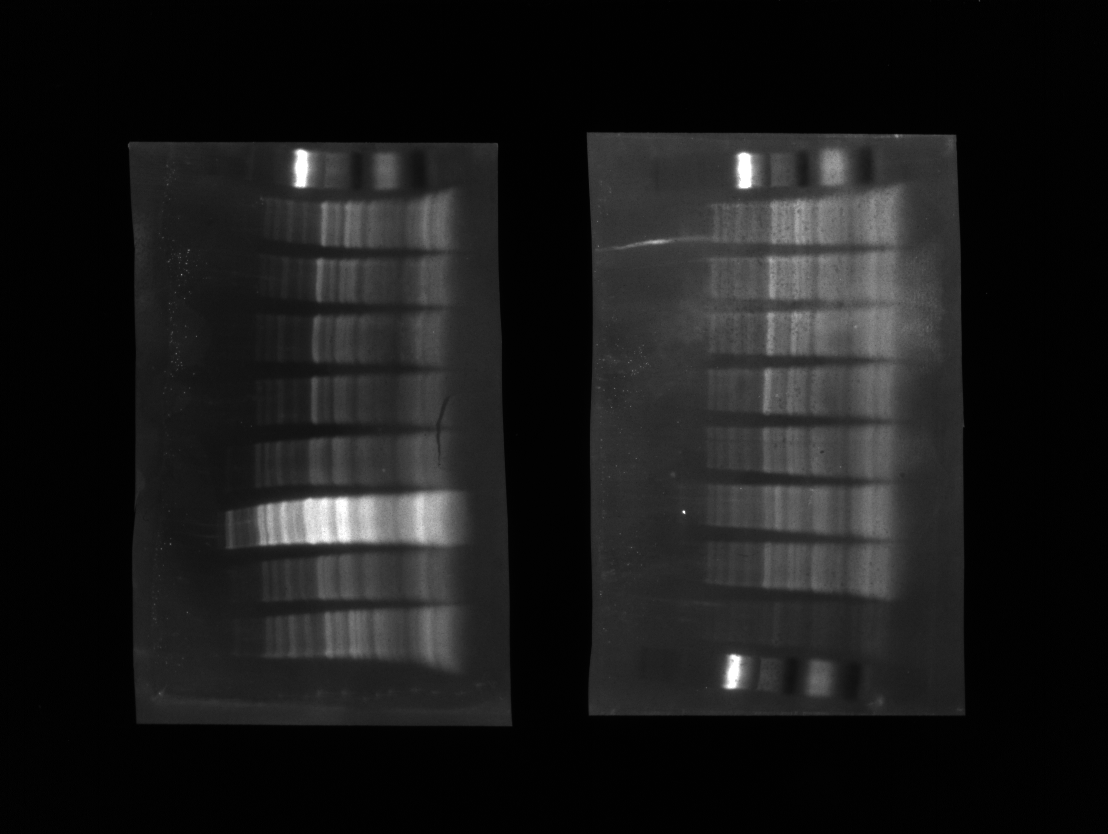

Supplement: Supplementary file 15 — Source data Fig. S4 [file 44318_2024_178_MOESM15_ESM.zip › Appendix Figure S4/D, E/Total protein/2021-12-20_13-16-30_1_16bit.png]

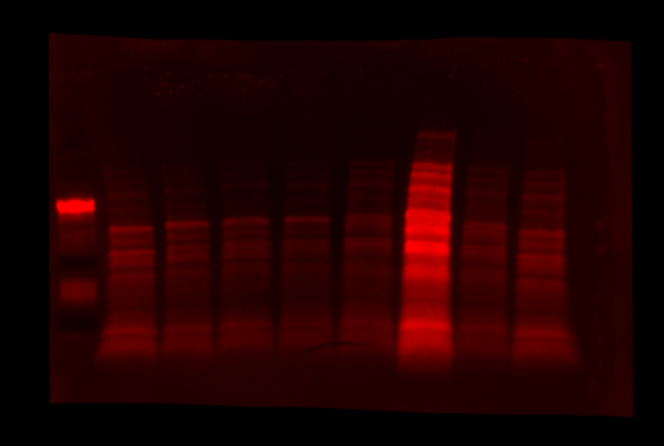

Supplement: Supplementary file 15 — Source data Fig. S4 [file 44318_2024_178_MOESM15_ESM.zip › Appendix Figure S4/D, E/Total protein/2021-12-20_13-16-30_8bit-1.png]

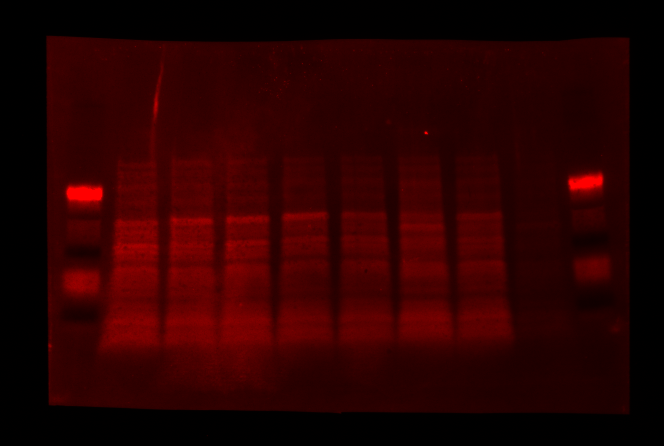

Supplement: Supplementary file 15 — Source data Fig. S4 [file 44318_2024_178_MOESM15_ESM.zip › Appendix Figure S4/D, E/Total protein/2021-12-20_13-16-30_8bit-1b.png]

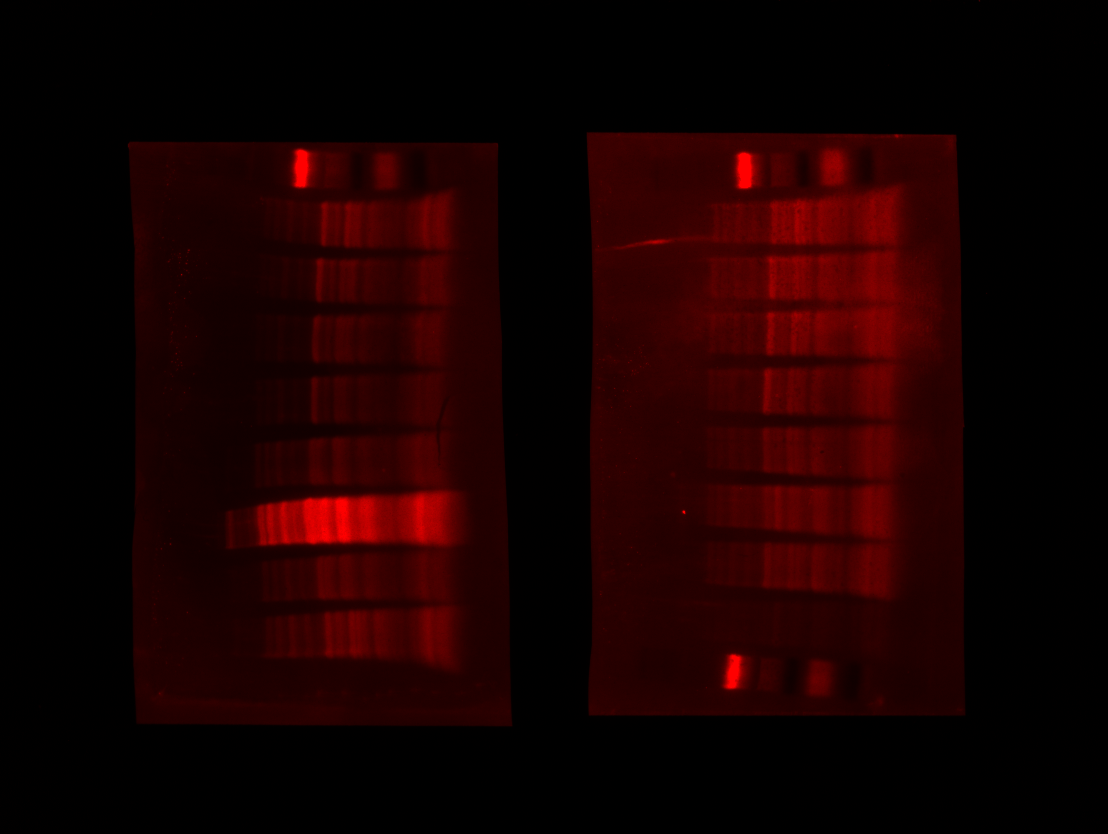

Supplement: Supplementary file 15 — Source data Fig. S4 [file 44318_2024_178_MOESM15_ESM.zip › Appendix Figure S4/D, E/Total protein/2021-12-20_13-16-30_8bit.png]

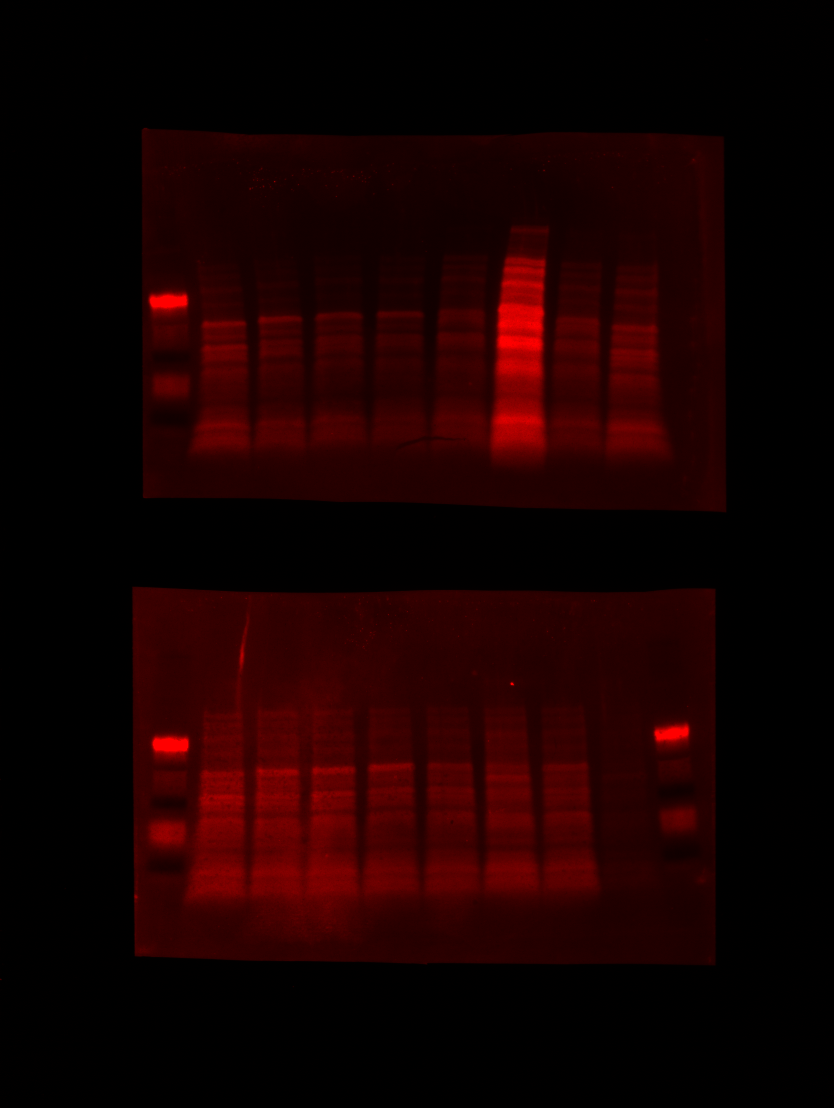

Supplement: Supplementary file 15 — Source data Fig. S4 [file 44318_2024_178_MOESM15_ESM.zip › Appendix Figure S4/D, E/Total protein/2021-12-20_13-16-30_8bit.tif]

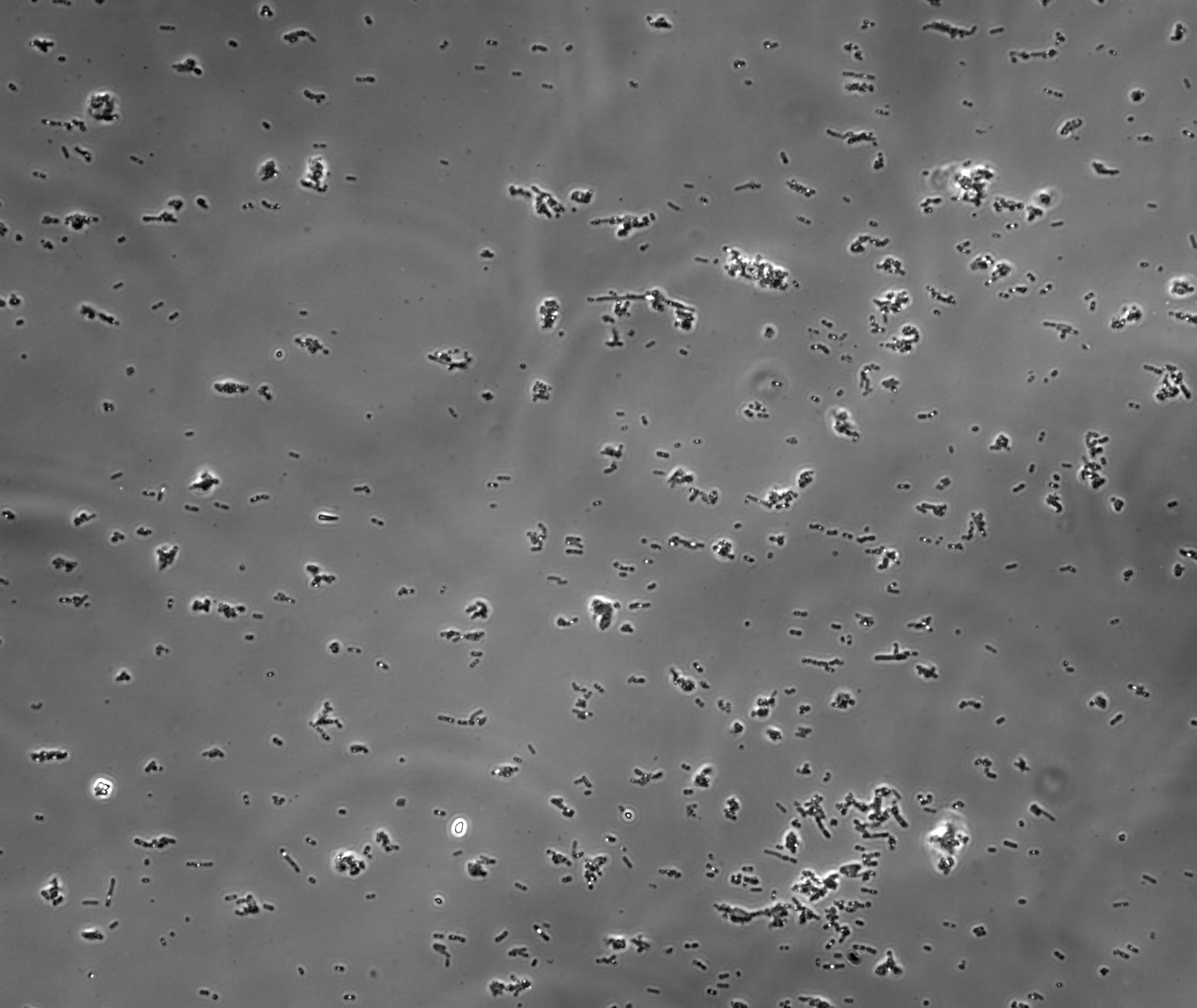

Supplement: Supplementary file 16 — EV and Appendix Figure Source Data Part 1 [file 44318_2024_178_MOESM16_ESM.zip › Appendix Figure S3/NSM TLG dNT 2.nd2 - C=0-1.tif]

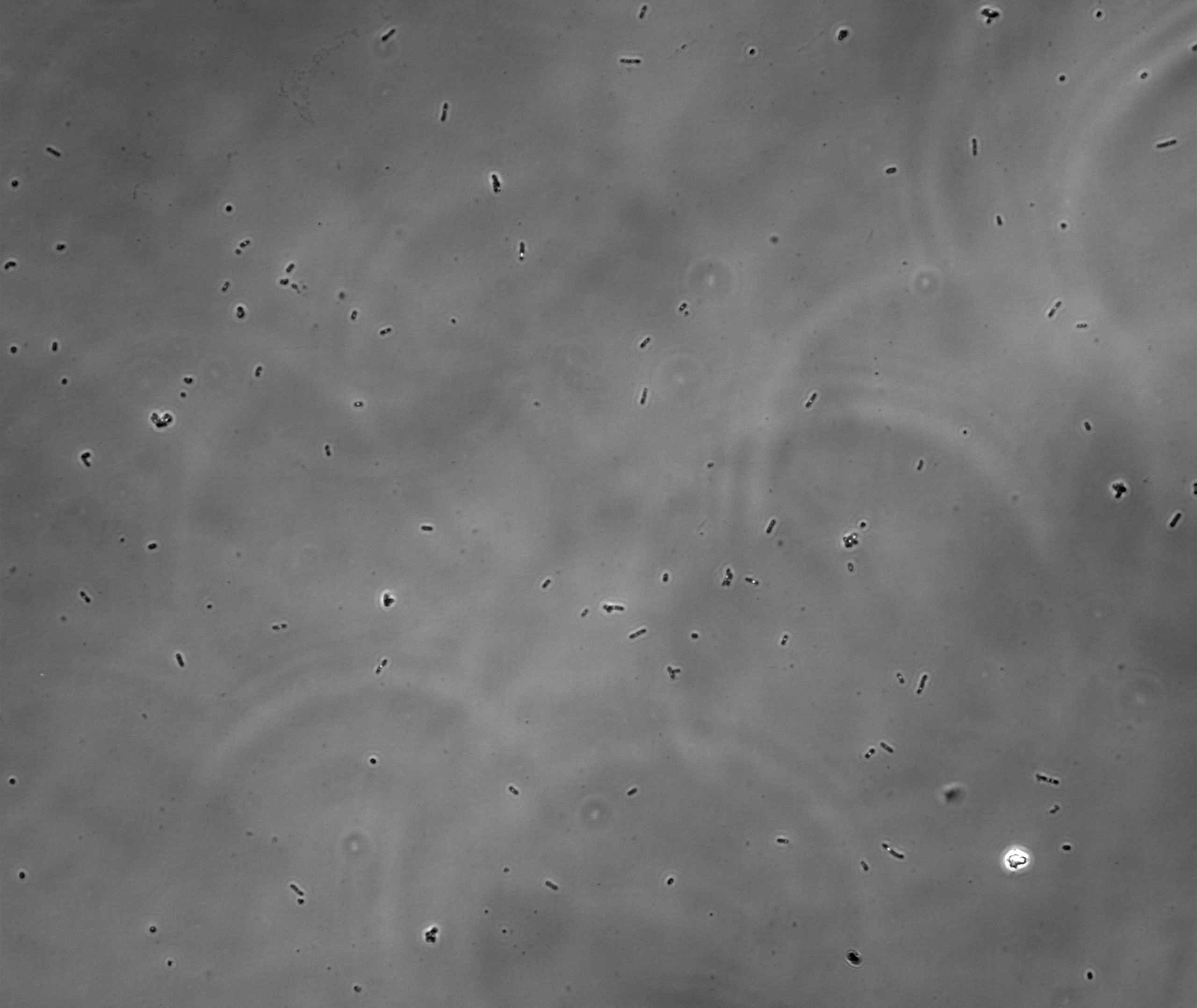

Supplement: Supplementary file 16 — EV and Appendix Figure Source Data Part 1 [file 44318_2024_178_MOESM16_ESM.zip › Appendix Figure S3/NLG TSM dNT 2.nd2 - C=0-1.tif]

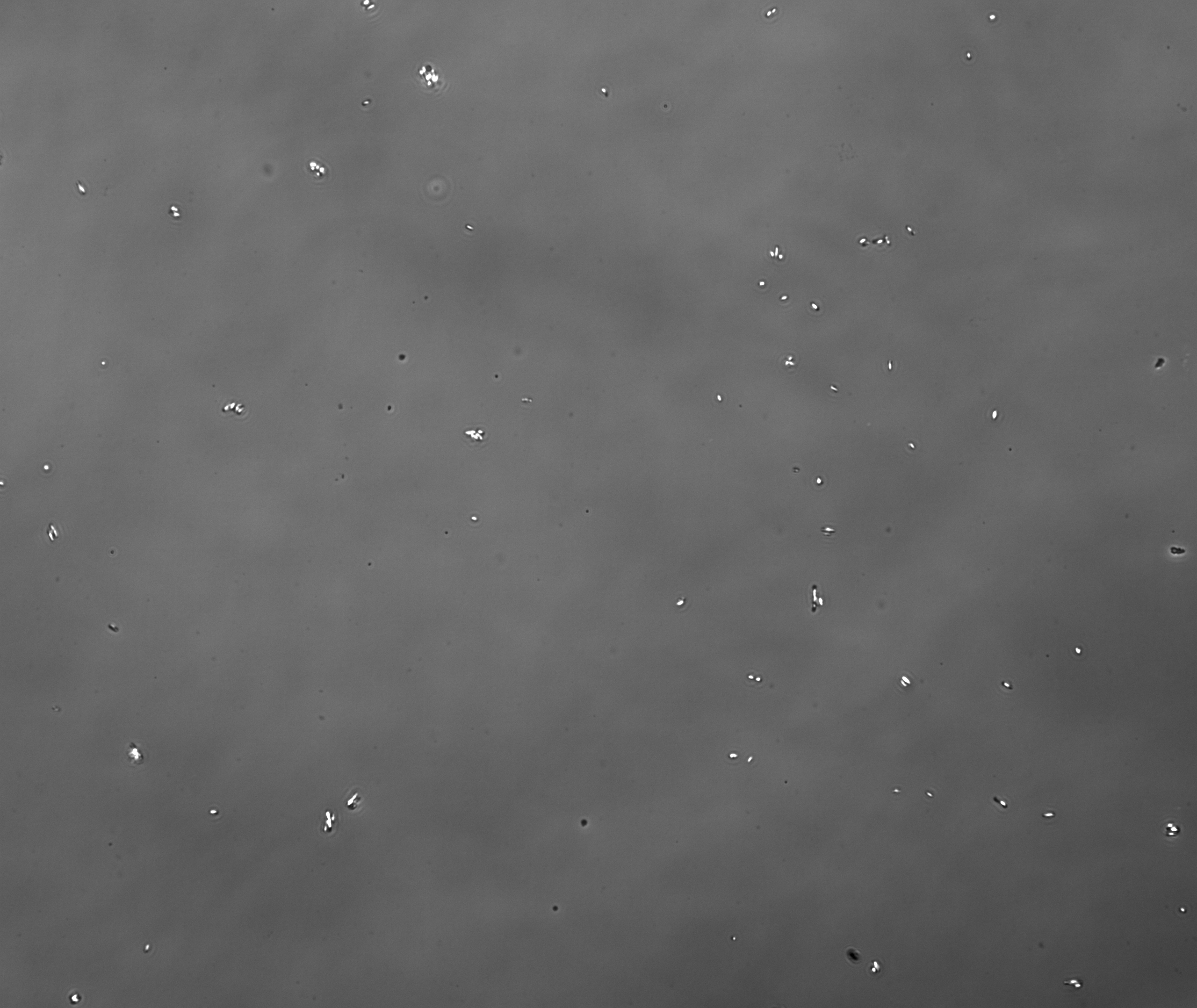

Supplement: Supplementary file 16 — EV and Appendix Figure Source Data Part 1 [file 44318_2024_178_MOESM16_ESM.zip › Appendix Figure S3/Mega GV (no fusion).nd2 - C=0-1.tif]

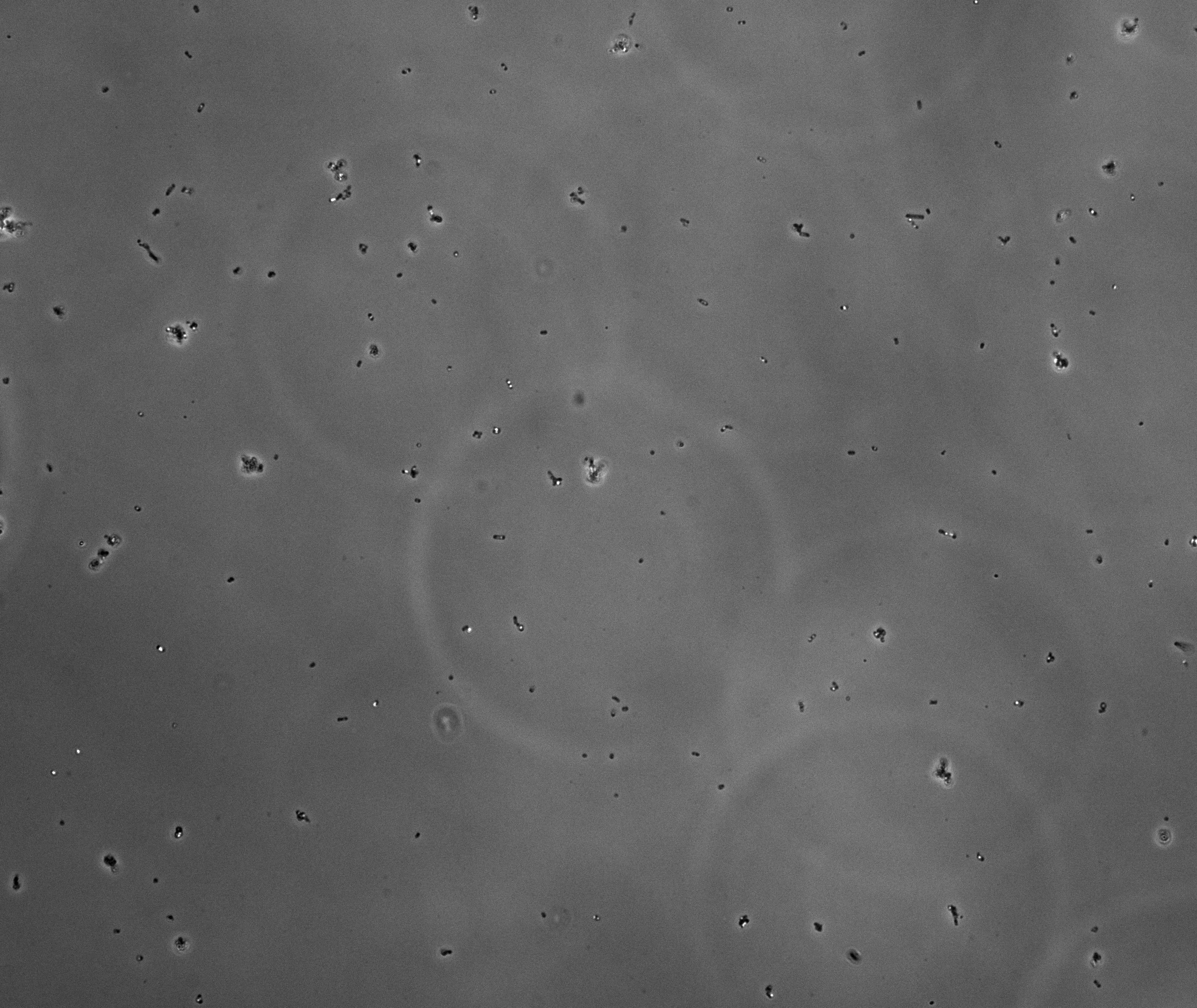

Supplement: Supplementary file 16 — EV and Appendix Figure Source Data Part 1 [file 44318_2024_178_MOESM16_ESM.zip › Appendix Figure S3/LGT NSM deltaNT.nd2 - C=0-1.tif]

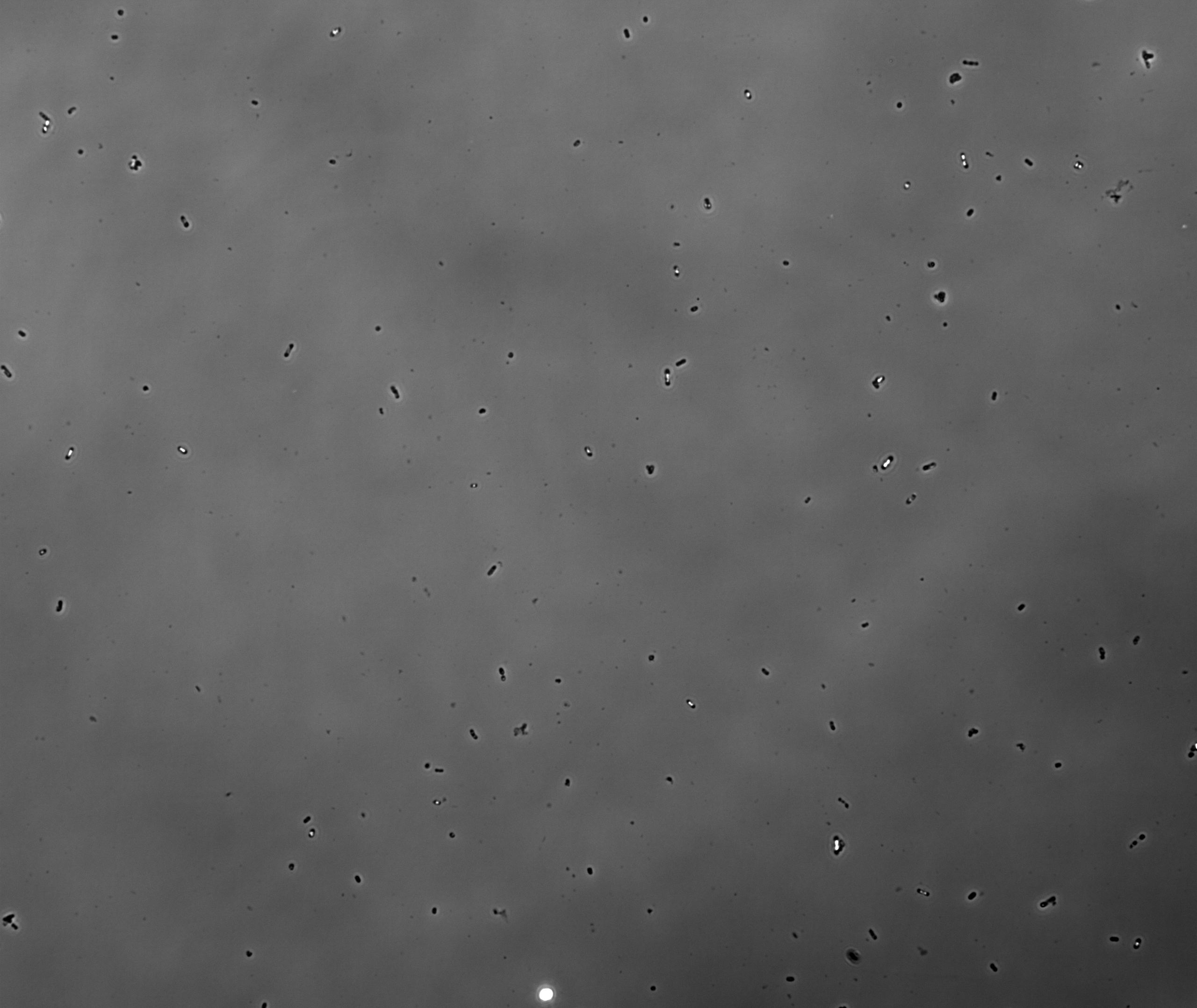

Supplement: Supplementary file 16 — EV and Appendix Figure Source Data Part 1 [file 44318_2024_178_MOESM16_ESM.zip › Appendix Figure S3/LGF FSM deltaF.nd2 - C=0-1.tif]

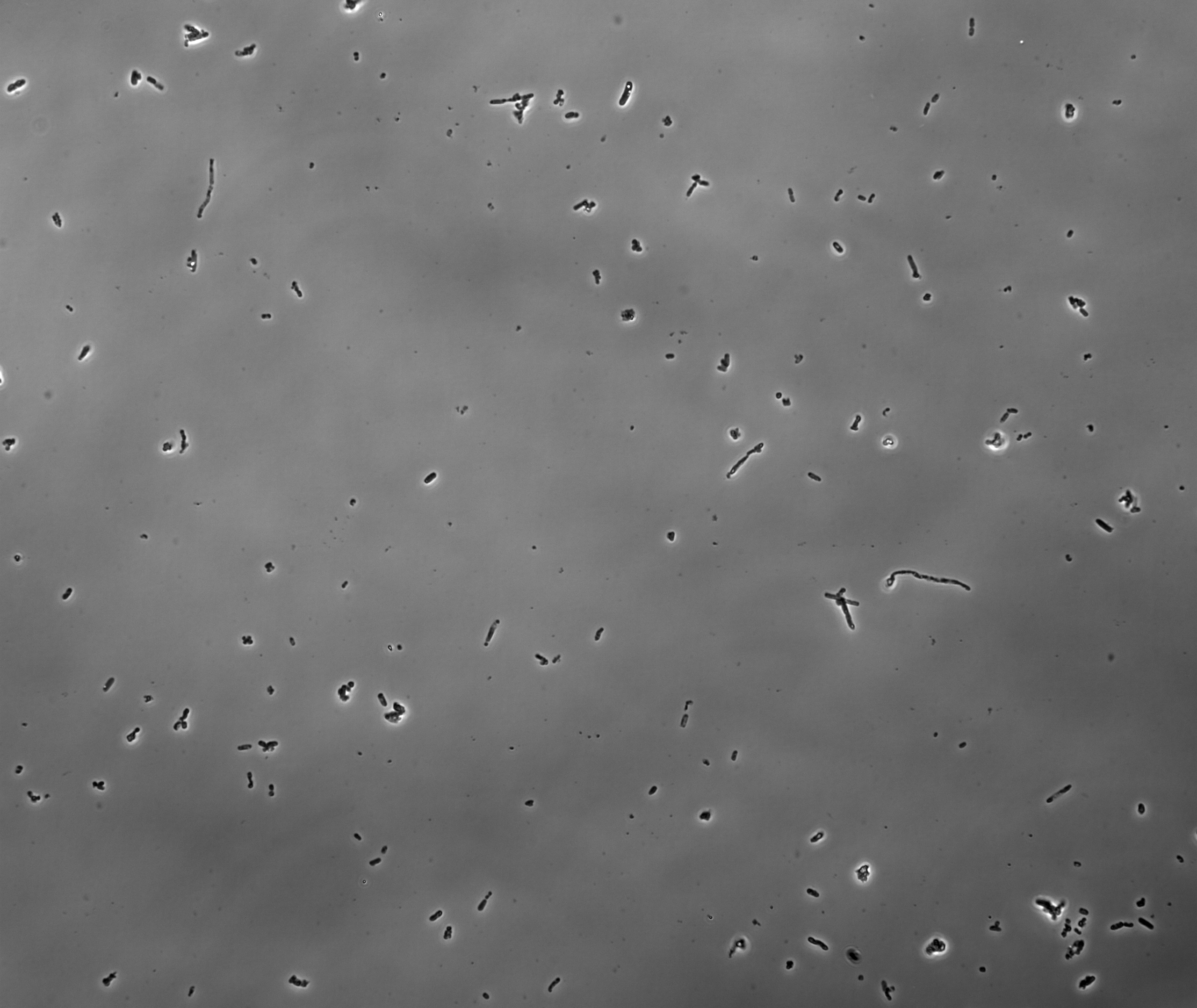

Supplement: Supplementary file 16 — EV and Appendix Figure Source Data Part 1 [file 44318_2024_178_MOESM16_ESM.zip › Appendix Figure S3/SLG KSM deltaSK.nd2 - C=0-1.tif]

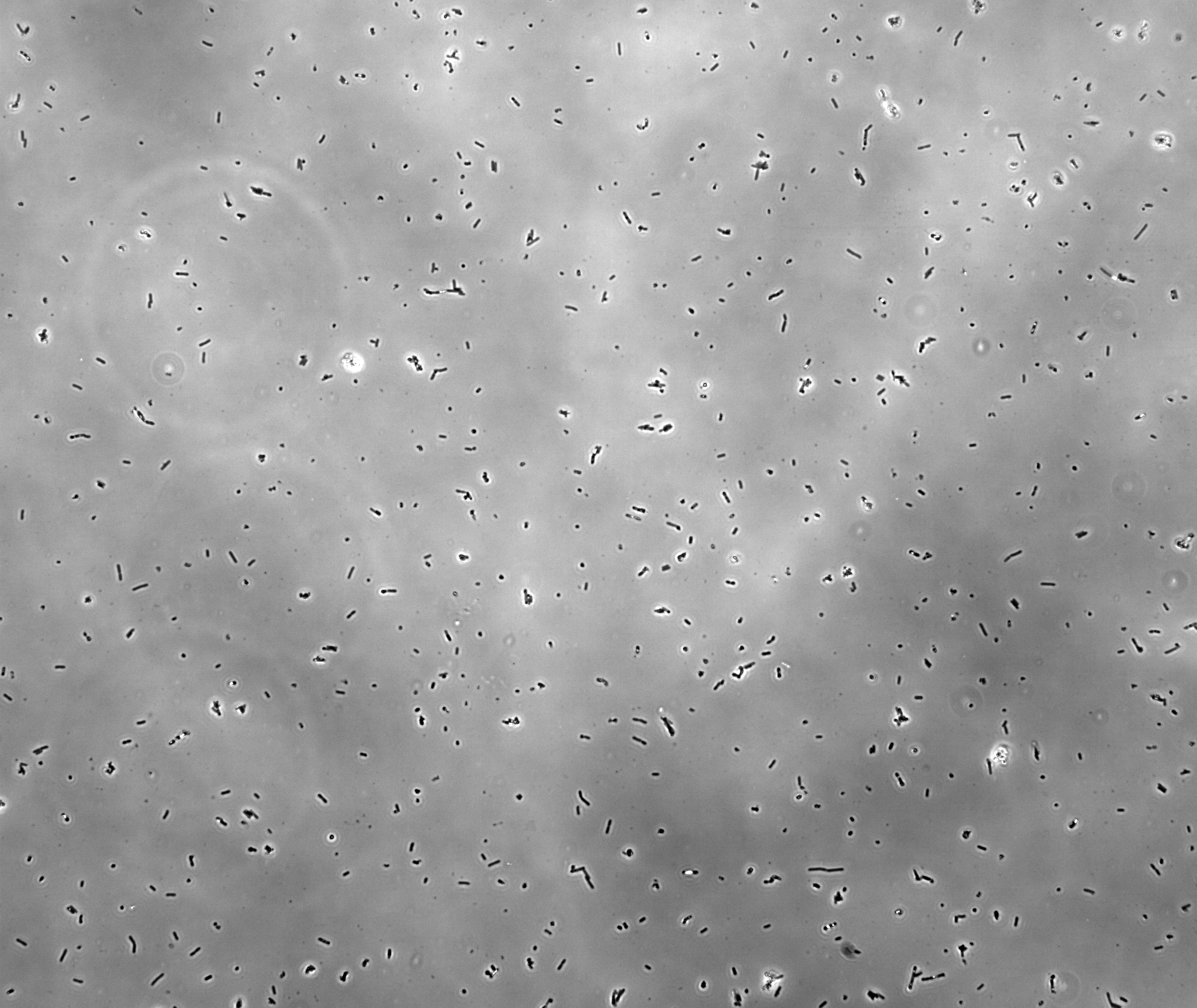

Supplement: Supplementary file 16 — EV and Appendix Figure Source Data Part 1 [file 44318_2024_178_MOESM16_ESM.zip › Appendix Figure S3/LGS SMT deltaST.nd2 - C=0.tif]

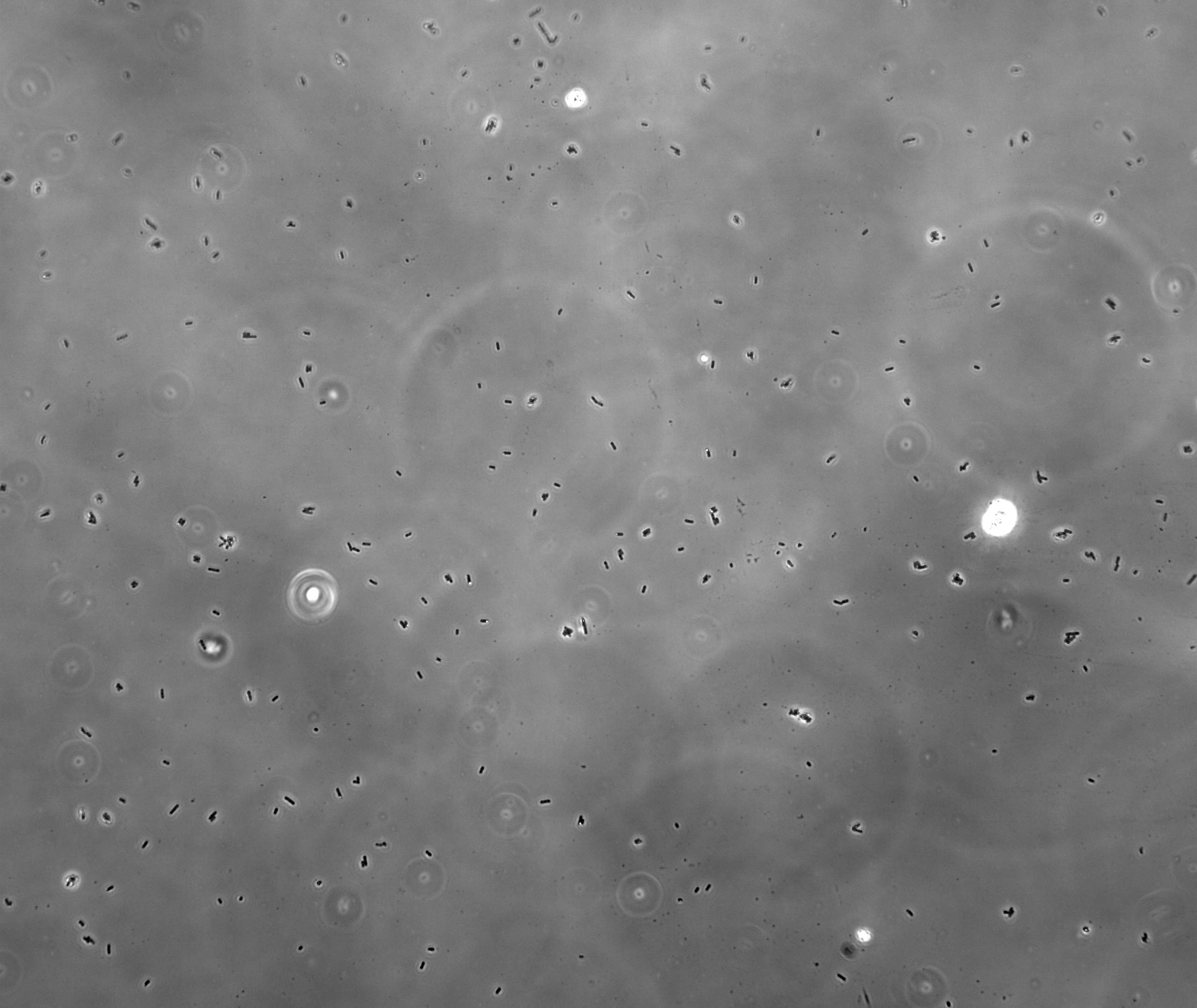

Supplement: Supplementary file 16 — EV and Appendix Figure Source Data Part 1 [file 44318_2024_178_MOESM16_ESM.zip › Appendix Figure S3/LGU SMS deltaSU.nd2 - C=0-1.tif]

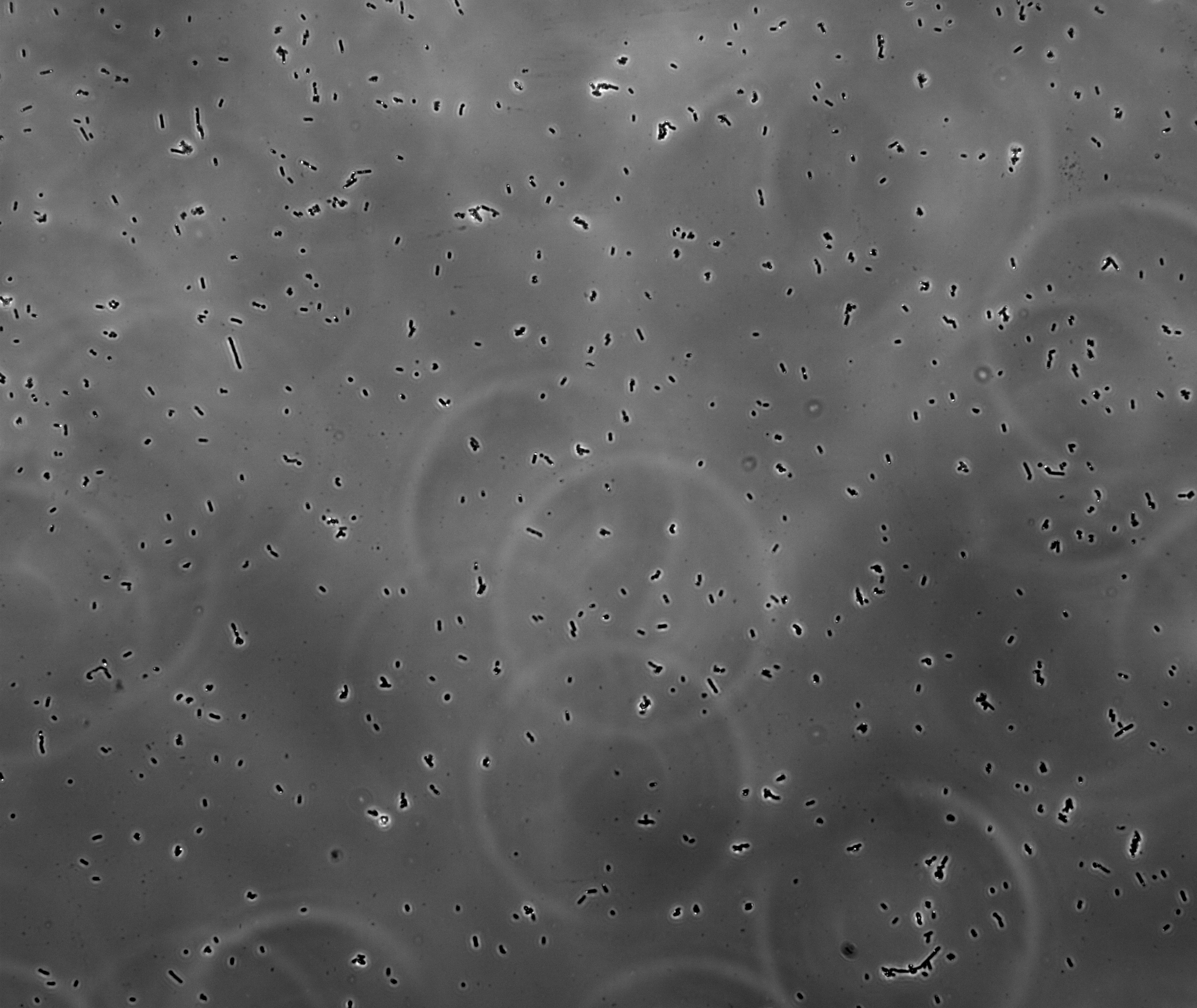

Supplement: Supplementary file 16 — EV and Appendix Figure Source Data Part 1 [file 44318_2024_178_MOESM16_ESM.zip › Appendix Figure S3/LGU SMK deltaKU.nd2 - C=0-1.tif]

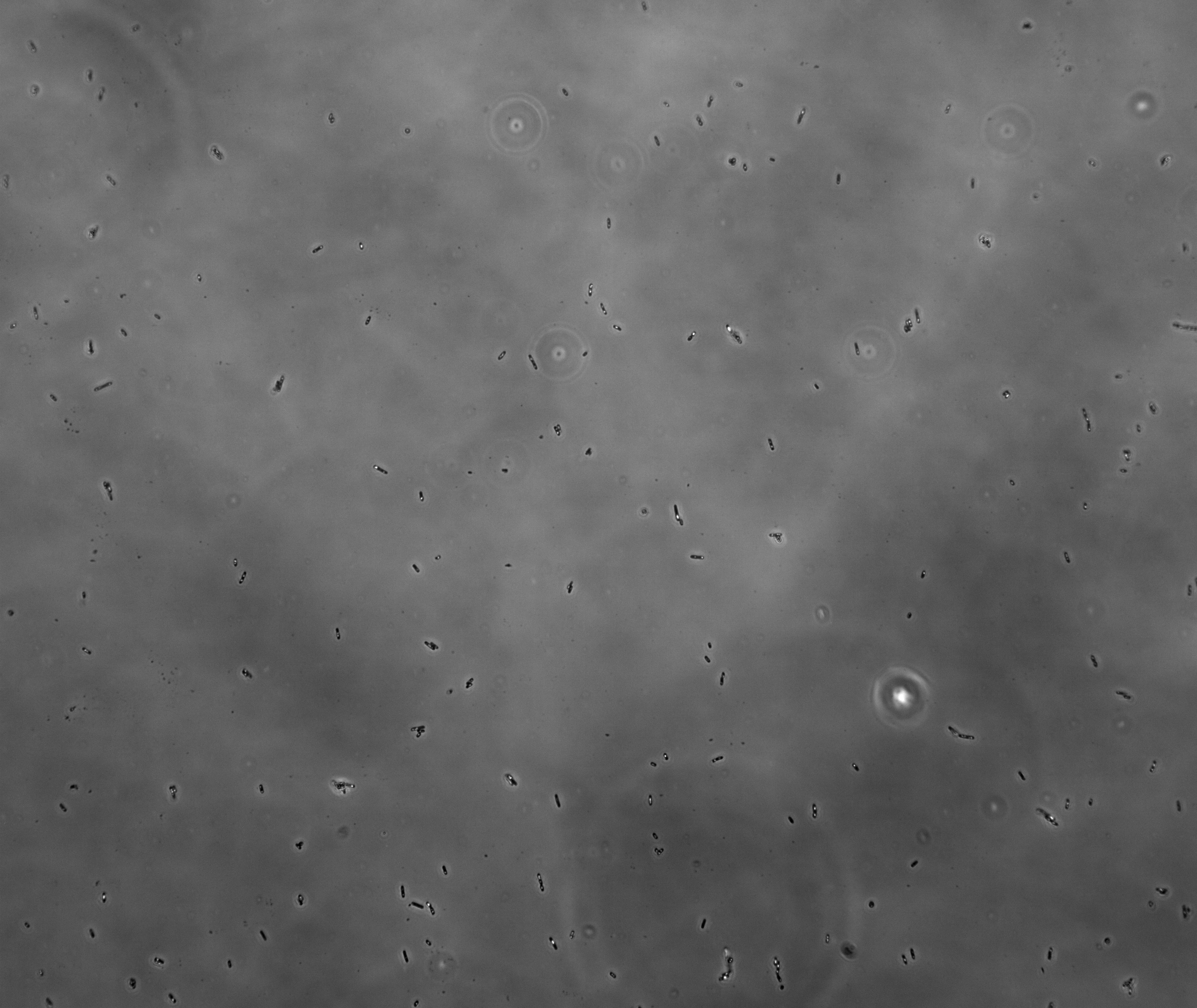

Supplement: Supplementary file 16 — EV and Appendix Figure Source Data Part 1 [file 44318_2024_178_MOESM16_ESM.zip › Appendix Figure S3/LGK SMT deltaKT.nd2 - C=0-1.tif]

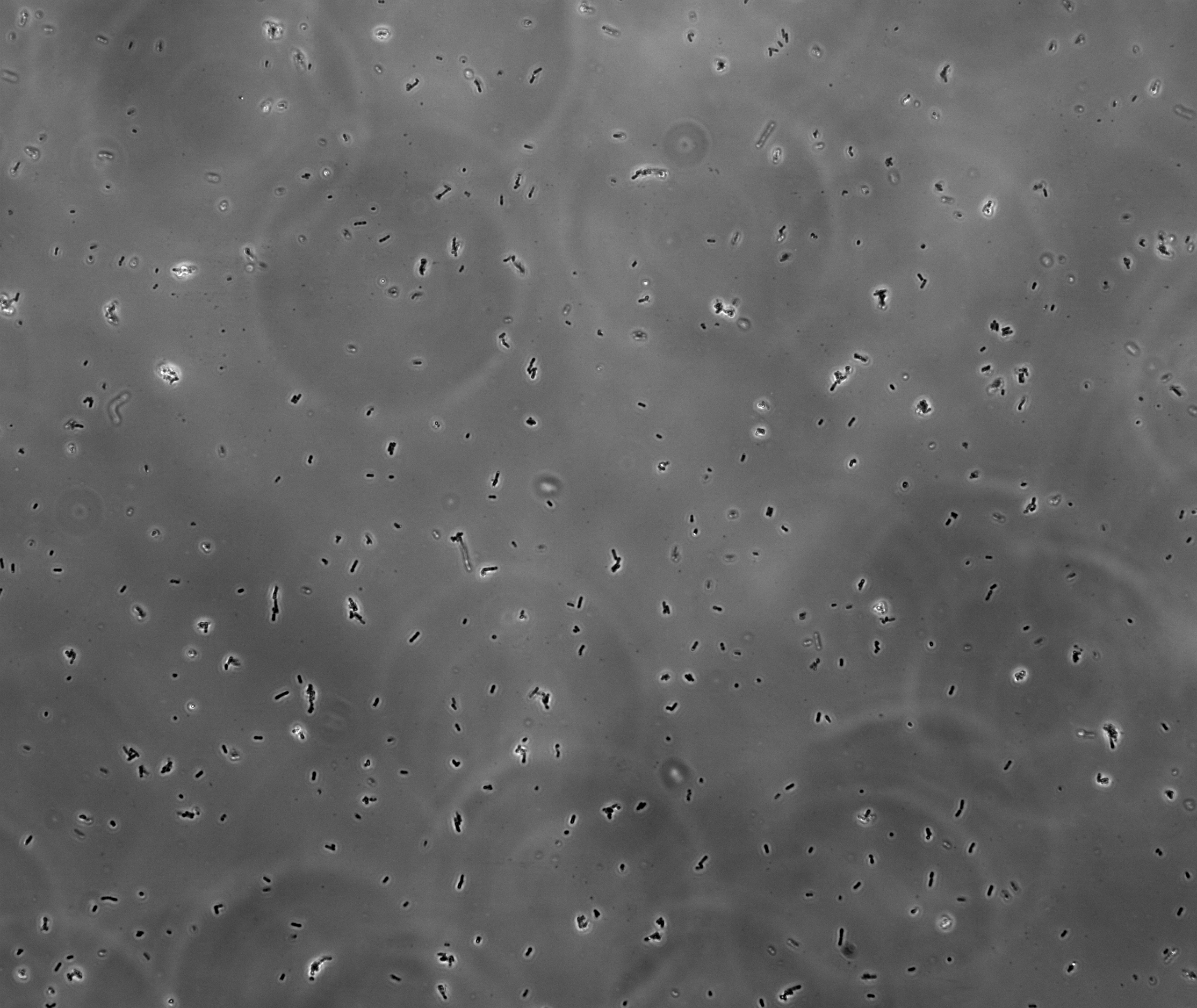

Supplement: Supplementary file 16 — EV and Appendix Figure Source Data Part 1 [file 44318_2024_178_MOESM16_ESM.zip › Appendix Figure S3/LGS SMU deltaSU.nd2 - C=0-1.tif]

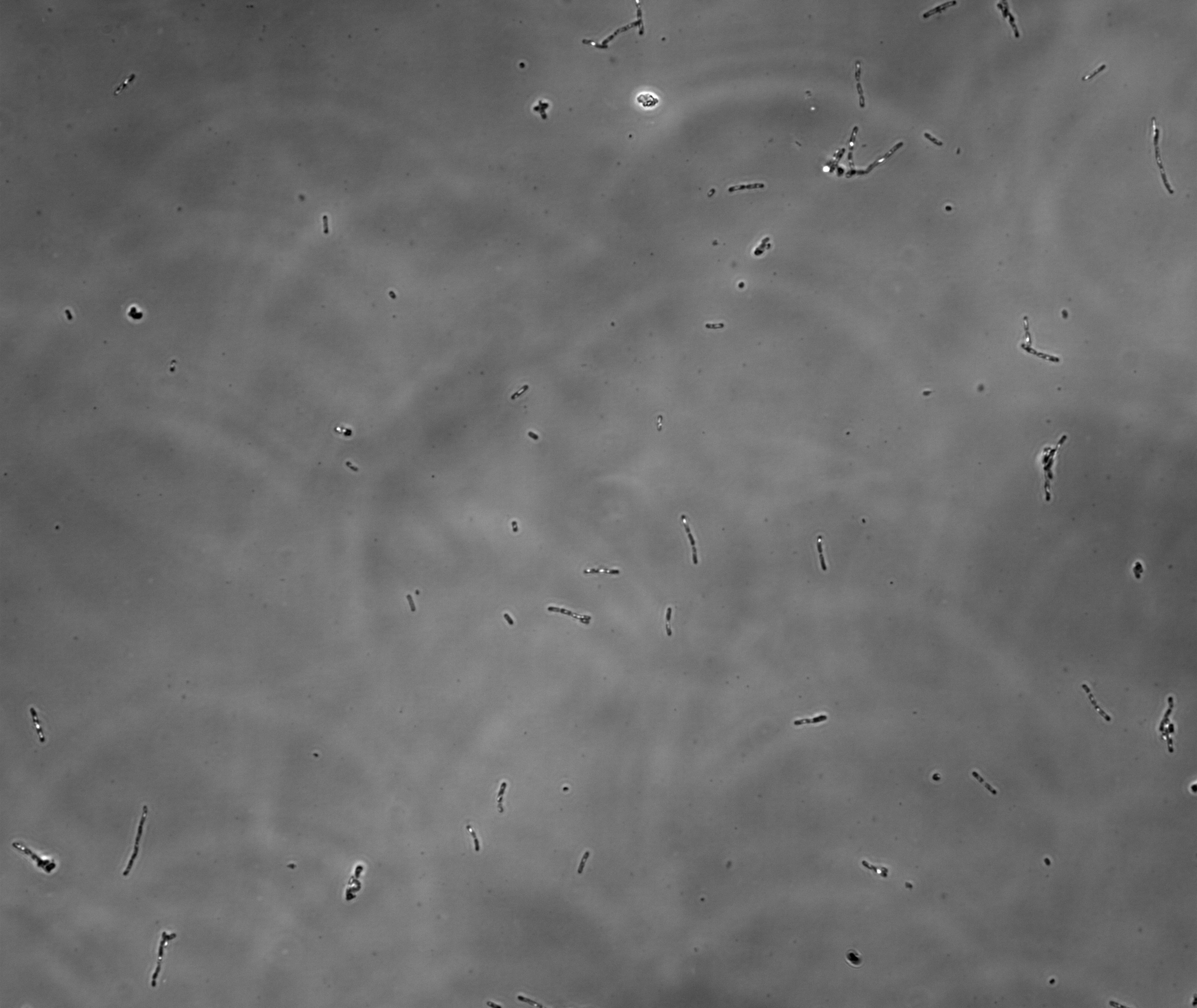

Supplement: Supplementary file 16 — EV and Appendix Figure Source Data Part 1 [file 44318_2024_178_MOESM16_ESM.zip › Appendix Figure S3/RLG SSM deltaRS.nd2 - C=0-1.tif]

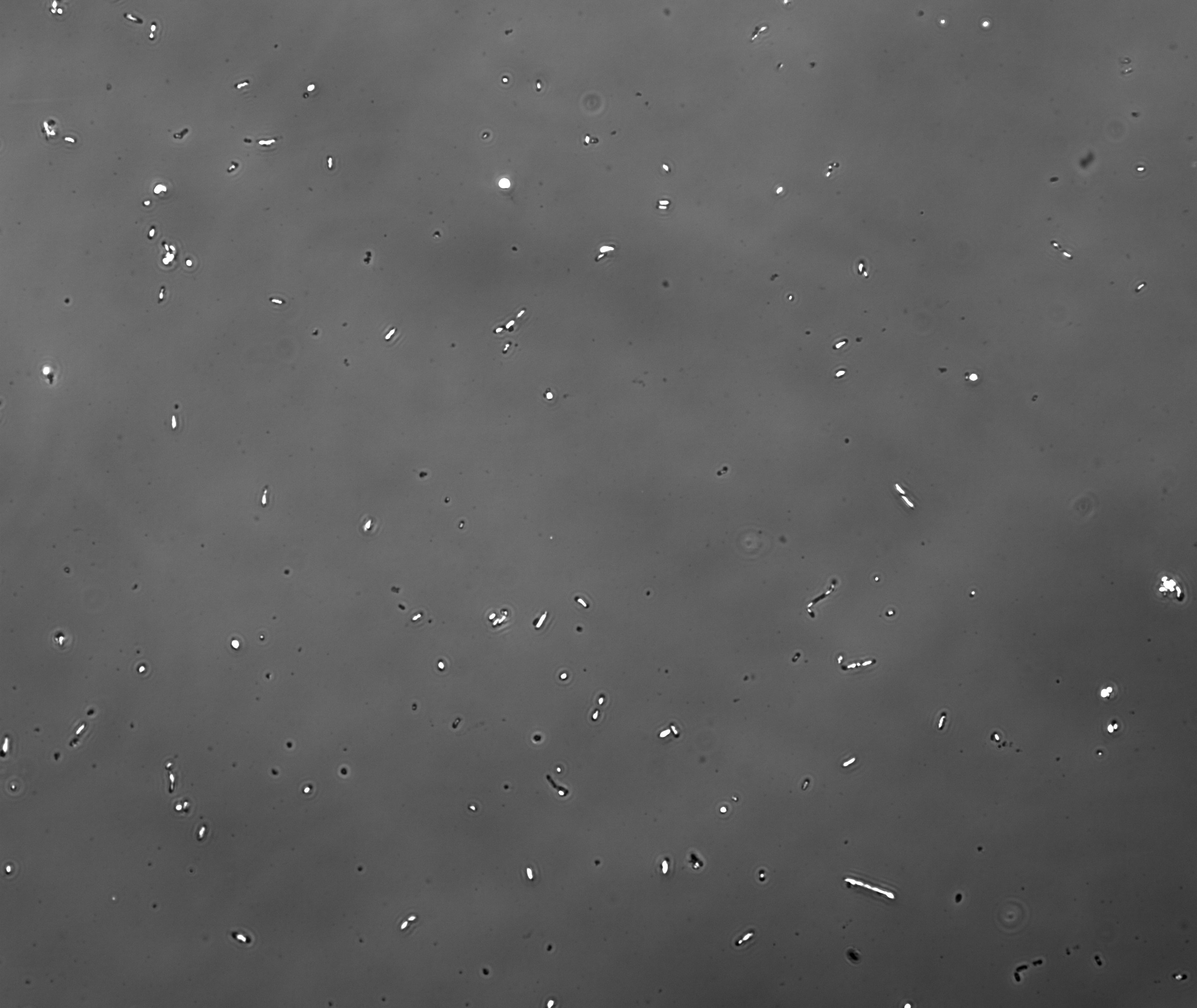

Supplement: Supplementary file 17 — EV and Appendix Figure Source Data Part 2 [file 44318_2024_178_MOESM17_ESM.zip › Table EV3/SLG SSM 3.nd2 - C=0-1.tif]

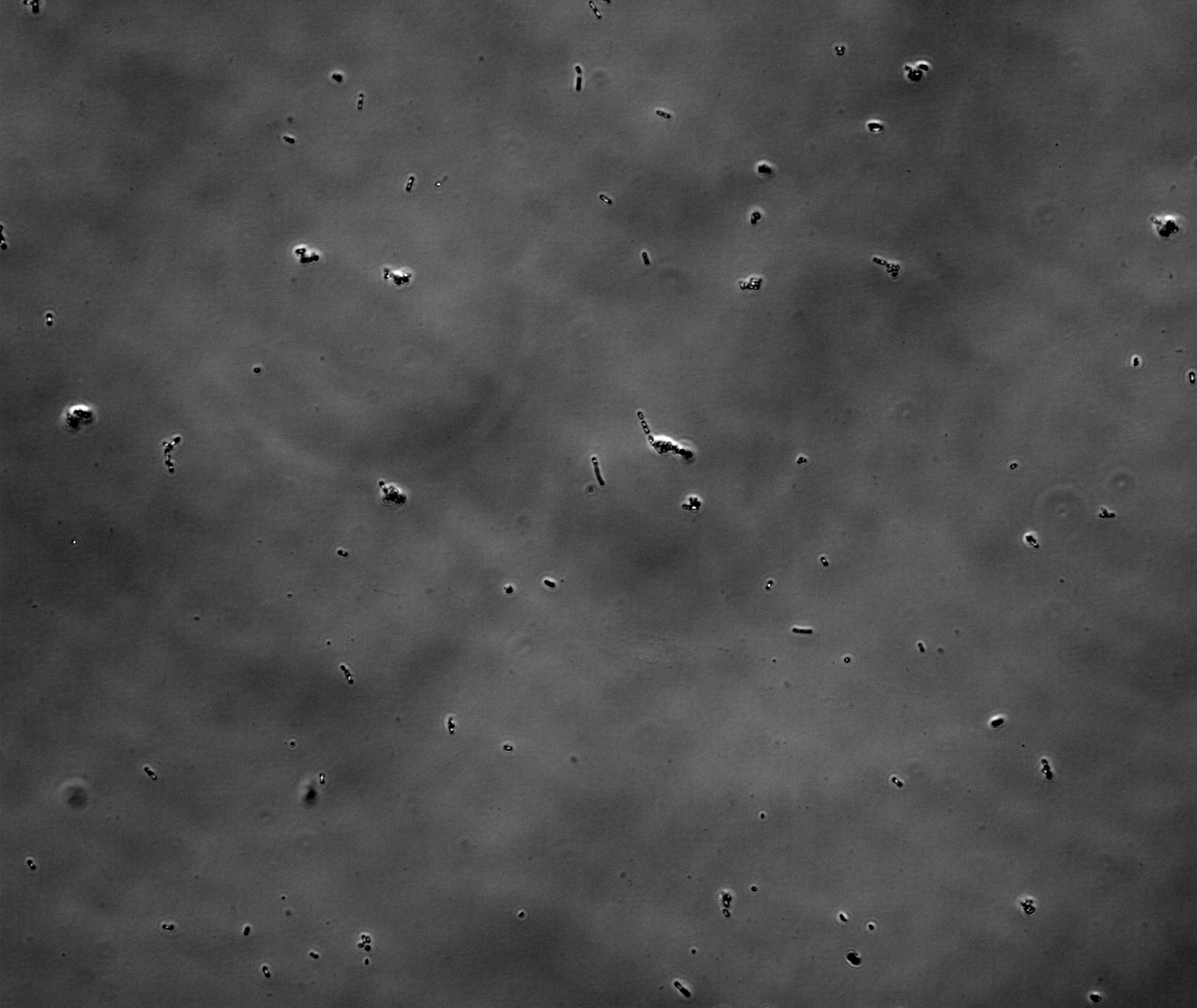

Supplement: Supplementary file 17 — EV and Appendix Figure Source Data Part 2 [file 44318_2024_178_MOESM17_ESM.zip › Table EV3/LGT SMT 2.nd2 - C=0-1.tif]

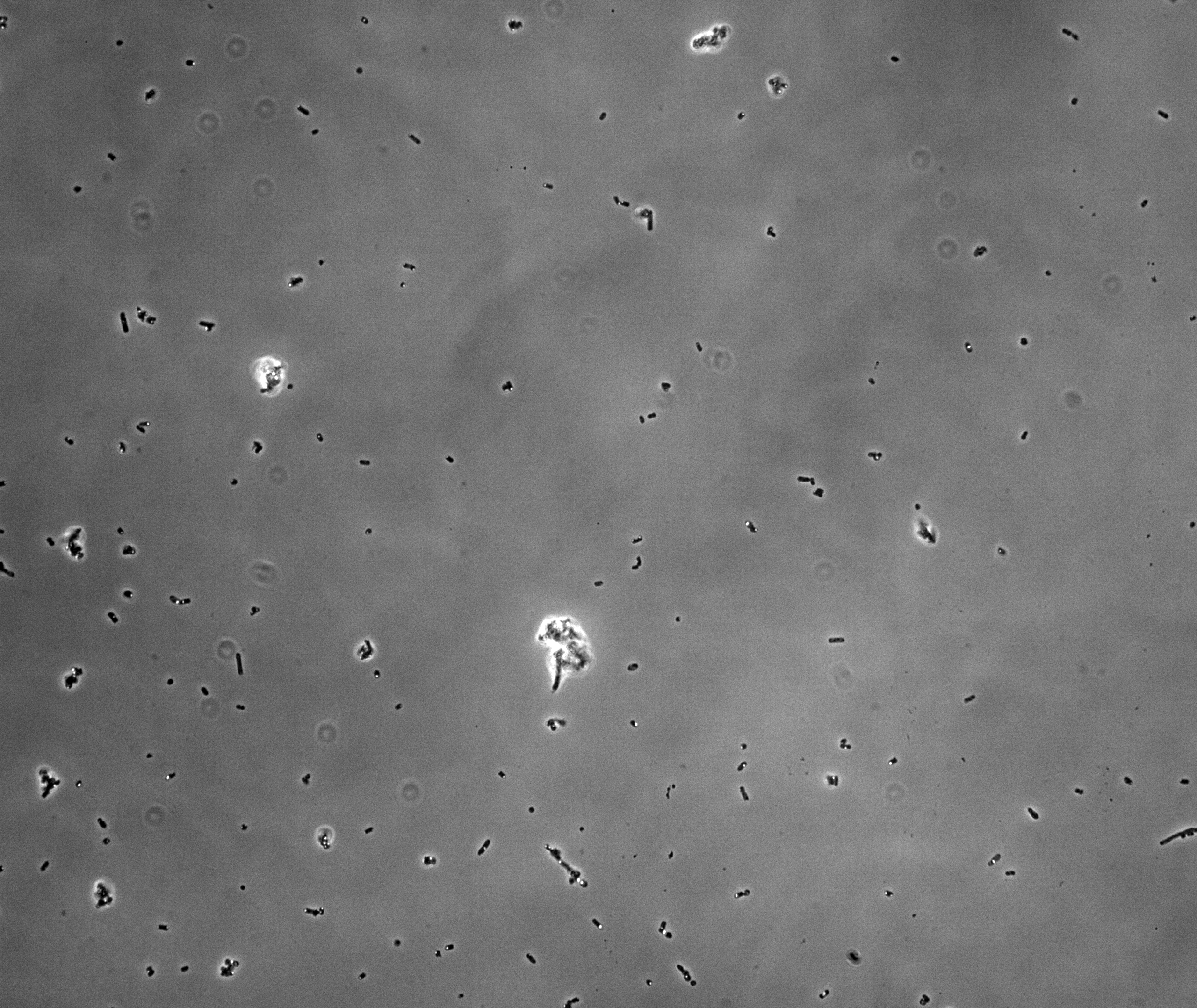

Supplement: Supplementary file 17 — EV and Appendix Figure Source Data Part 2 [file 44318_2024_178_MOESM17_ESM.zip › Table EV3/LGS SSM 2.nd2 - C=0-1.tif]

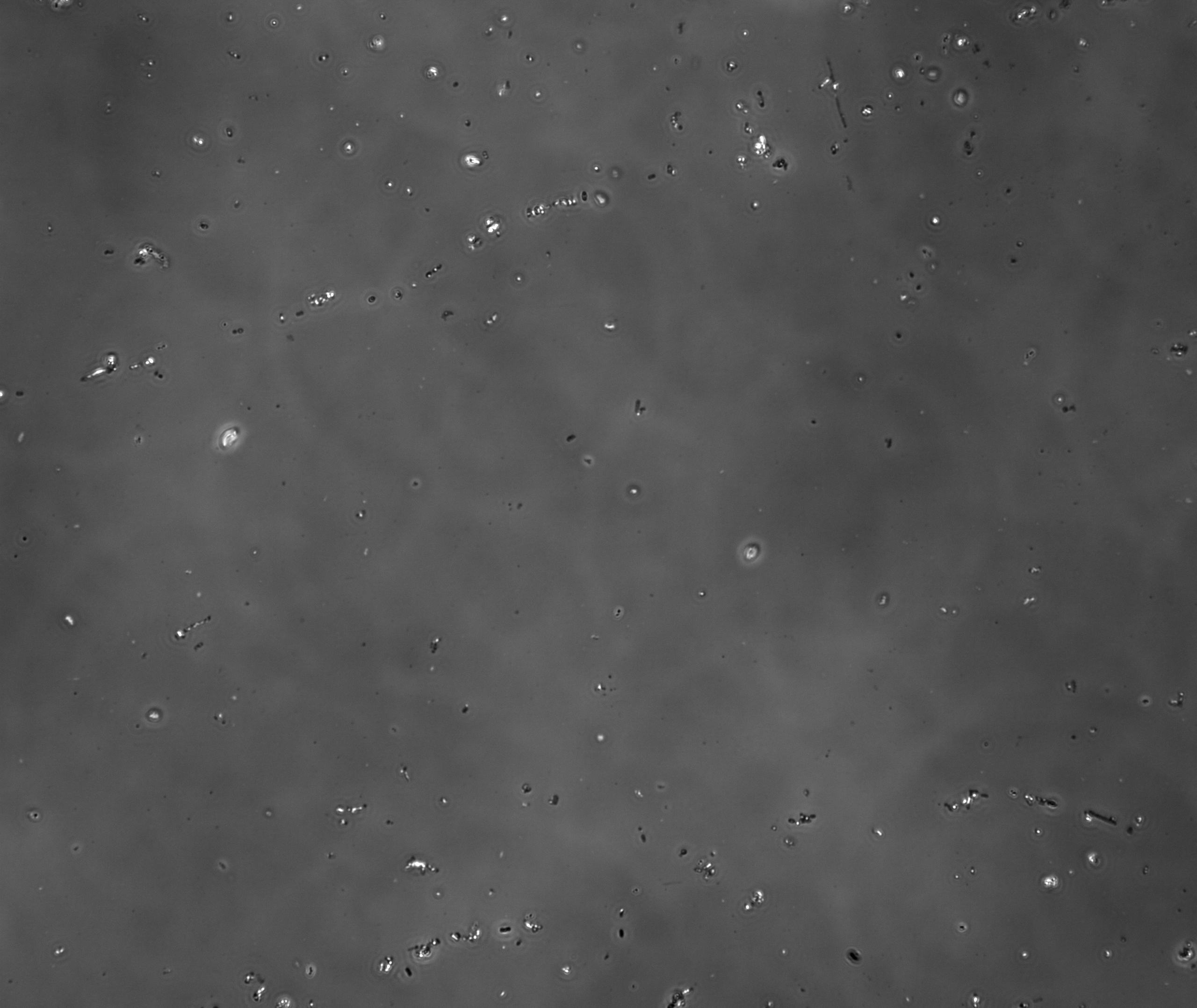

Supplement: Supplementary file 17 — EV and Appendix Figure Source Data Part 2 [file 44318_2024_178_MOESM17_ESM.zip › Table EV3/LGT TSM 2.nd2 - C=0-1.tif]

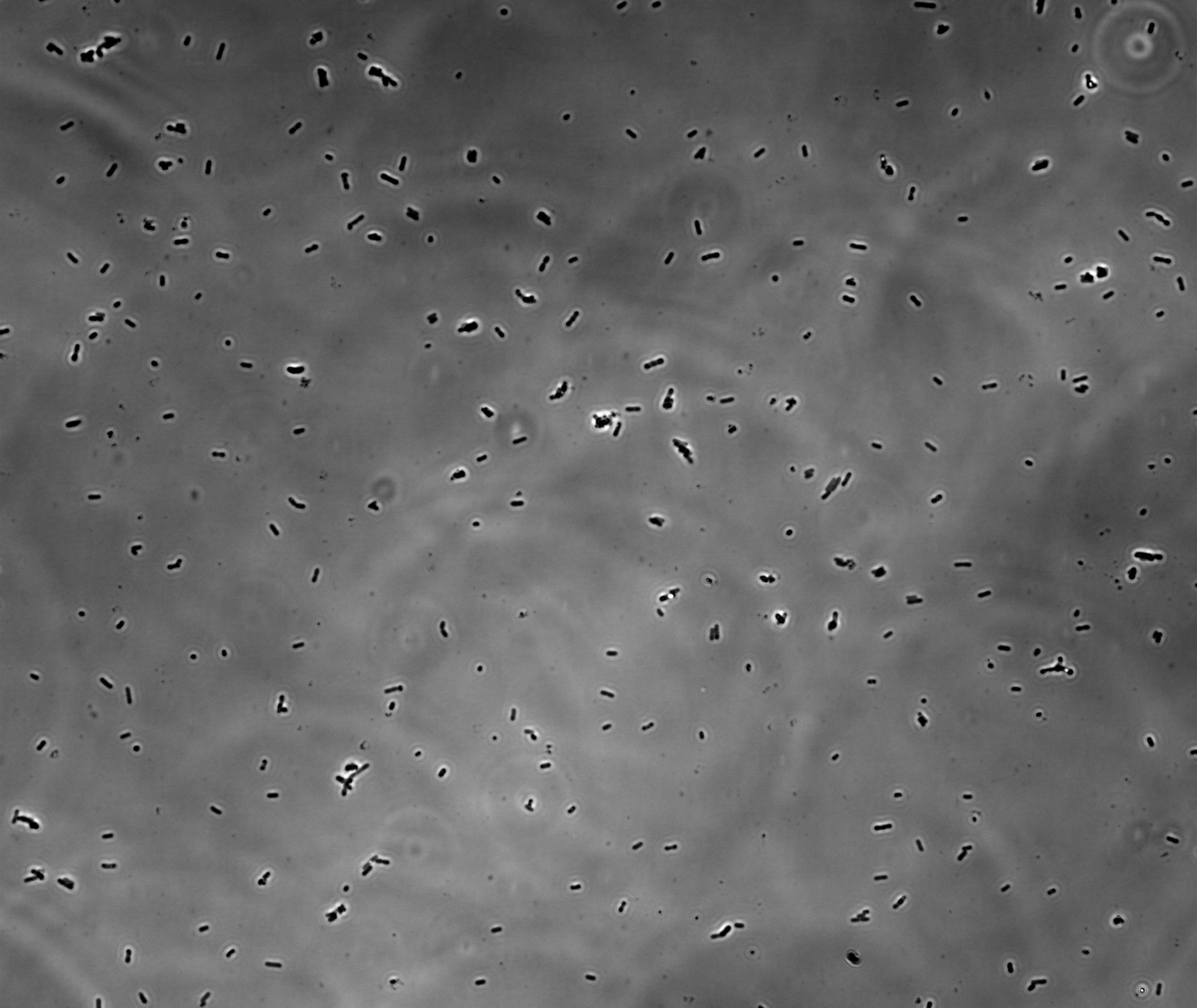

Supplement: Supplementary file 17 — EV and Appendix Figure Source Data Part 2 [file 44318_2024_178_MOESM17_ESM.zip › Table EV3/LGF SMN 4.nd2 - C=0-1.tif]

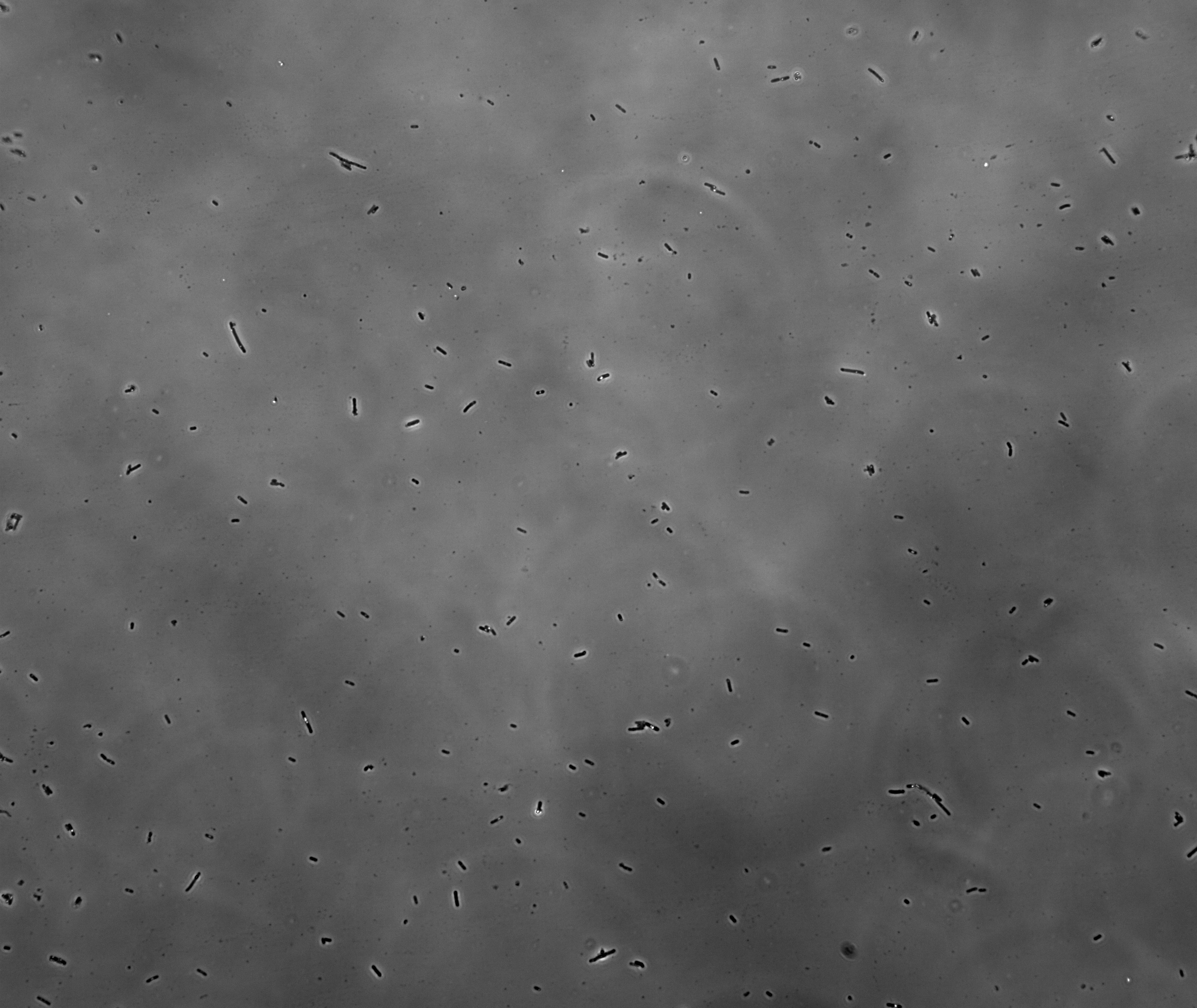

Supplement: Supplementary file 17 — EV and Appendix Figure Source Data Part 2 [file 44318_2024_178_MOESM17_ESM.zip › Table EV3/RLG KSM 3.nd2 - C=0-1.tif]

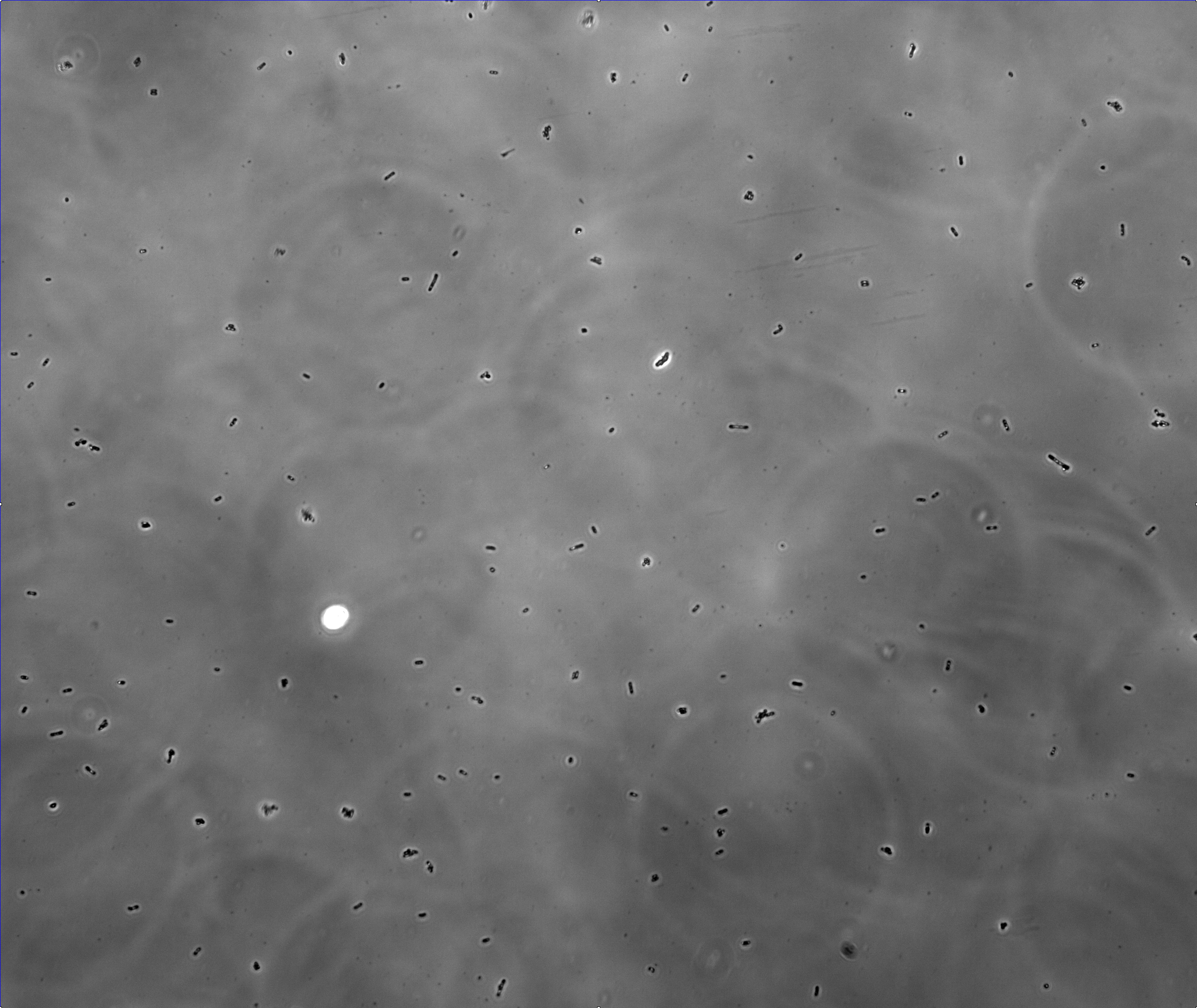

Supplement: Supplementary file 17 — EV and Appendix Figure Source Data Part 2 [file 44318_2024_178_MOESM17_ESM.zip › Table EV3/KLG SMK 1.nd2 - C=0-1.tif]

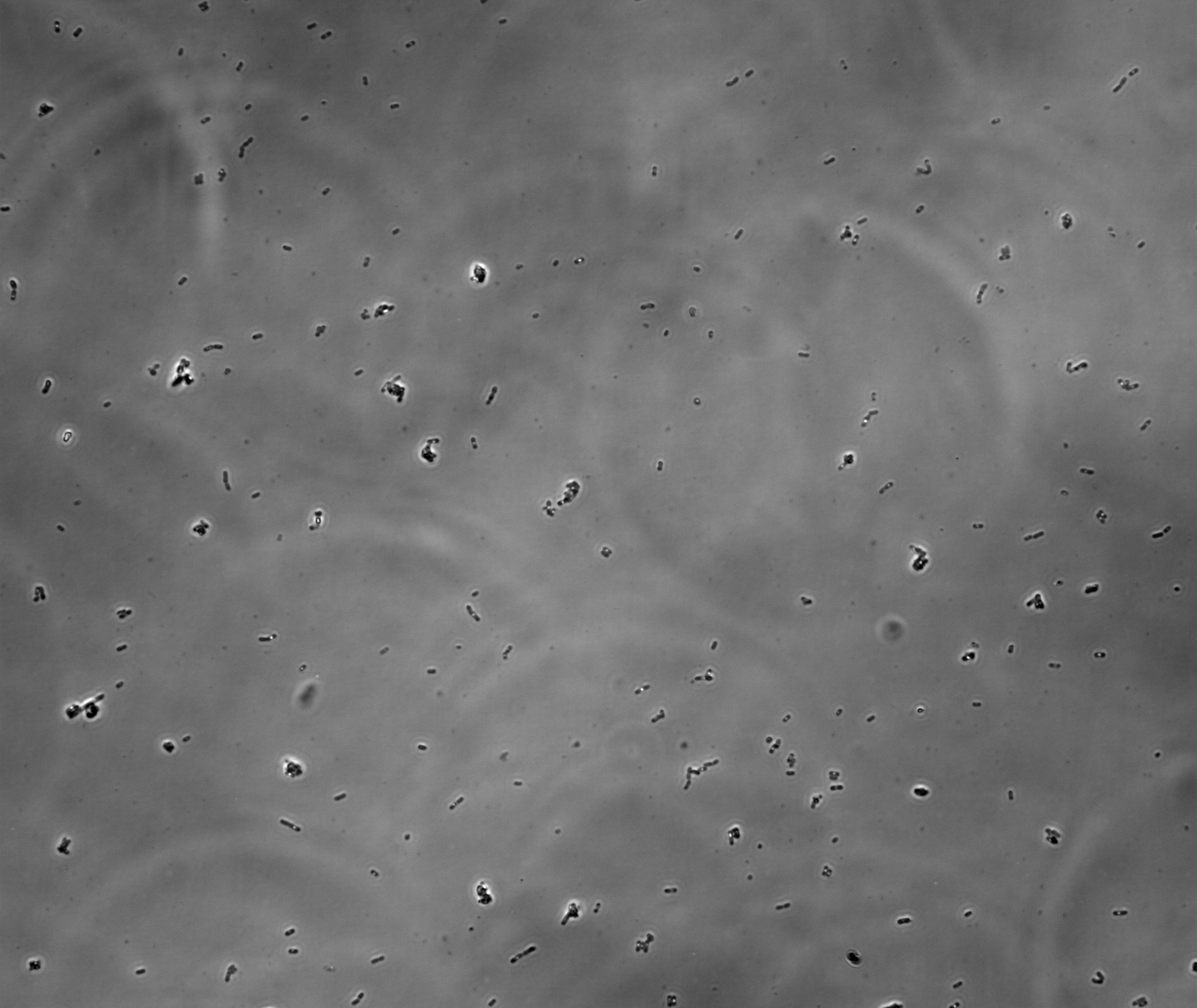

Supplement: Supplementary file 17 — EV and Appendix Figure Source Data Part 2 [file 44318_2024_178_MOESM17_ESM.zip › Table EV3/NLG NSM dN.nd2 - C=0-1.tif]
